# Discovering master regulators in hepatocellular carcinoma: one novel MR, SEC14L2 inhibits cancer cells

**DOI:** 10.18632/aging.102579

**Published:** 2019-12-18

**Authors:** Zhihui Li, Yi Lou, Guoyan Tian, Jianyue Wu, Anqian Lu, Jin Chen, Beibei Xu, Junping Shi, Jin Yang

**Affiliations:** 1 Translational Medicine Center, The Affiliated Hospital of Hangzhou Normal University, Hangzhou, Zhejiang 310015, P.R. China; 2 Department of Occupational Medicine, Zhejiang Provincial Integrated Chinese and Western Medicine Hospital, Hangzhou, Zhejiang 310003, P.R. China

**Keywords:** liver cancer, master regulator, transcriptional network, *SEC14L2*

## Abstract

Identification of master regulator (MR) genes offers a relatively rapid and efficient way to characterize disease-specific molecular programs. Since strong consensus regarding commonly altered MRs in hepatocellular carcinoma (HCC) is lacking, we generated a compendium of HCC datasets from 21 studies and identified a comprehensive signature consisting of 483 genes commonly deregulated in HCC. We then used reverse engineering of transcriptional networks to identify the MRs that underpin the development and progression of HCC. After cross-validation in different HCC datasets, systematic assessment using patient-derived data confirmed prognostic predictive capacities for most HCC MRs and their corresponding regulons. Our HCC signature covered well-established liver cancer hallmarks, and network analyses revealed coordinated interaction between several MRs. One novel MR, *SEC14L2*, exerted an anti-proliferative effect in HCC cells and strongly suppressed tumor growth in a mouse model. This study advances our knowledge of transcriptional MRs potentially involved in HCC development and progression that may be targeted by specific interventions.

## INTRODUCTION

Hepatocellular carcinoma (HCC) is one of the five leading causes of cancer-related death worldwide. Recent developments in the treatment of HCC proved insufficient to cure unresectable disease and to substantially prevent HCC progression. The limitations of current therapeutics primarily reflect incomplete understanding of the complex molecular signaling processes contributing to the heterogeneous nature of the disease [1].

Liver carcinogenesis is a multistep, long process associated with multiple risk factors, e.g. viral hepatitis, alcohol abuse, metabolic disorders, and obesity [2]. Over the last two decades, studies based on genome-wide gene expression and functional profiling have revealed the great diversity of transcriptional alterations occurring in liver carcinogenesis. However, translating these findings into individualized treatments has proved to be difficult [2].

Transcription factors (TFs) drive gene expression programs that shape specific phenotypes [3], and are frequently dysregulated in cancer [4]. Correspondingly, most cancer signaling pathways seem to converge on one or more TFs, termed “master regulators” (MRs) [4], which direct tumor development, progression, and metastasis through hierarchical control of gene expression patterns. Thus, MRs comprise typically a small number of TF-encoding genes (and their products) that control a disproportionate level of gene expression, giving rise to distinct molecular phenotypes associated with a particular disease. Therefore, identification and functional characterization of MRs is critical to understand associated disease processes and design effective therapeutic options [5]. For instance, a recent study indicated that *SOX4* acted as a MR of epithelial-to-mesenchymal transition (EMT) in HCC [6]. However, studies evaluating the presence of MRs in HCC are sparse and have yielded limited insight into cancer risk.

Since the expression of genes defining discrete phenotypes is highly coordinated, application of reverse engineering algorithms to transcriptome datasets allows interpreting transcriptional networks by defining MRs and their associated regulons and gene circuits. Focusing on computational and statistical aspects of MR discovery, the ARACNe-MRA (Algorithm for the Reconstruction of Accurate Cellular Networks-Master Regulator Analysis) method has shown competent performance in this regard [4]. Using ARACNe-MRA, researchers have successfully identified prognostically relevant MRs in glioma, ovarian, breast, and prostate cancer [4, 7].

In the present study, we model liver cancer through a TF-centered regulatory network derived from HCC transcriptional datasets, followed by MRA analysis of changes in transcriptional regulatory programs related to tumor phenotype. Our analysis provides insights into the gene regulatory circuits operating in HCC and has implications for the identification of novel therapeutic targets.

## RESULTS

### Generation of a compendium of gene expression profiles in human HCC

**Figure 1:**
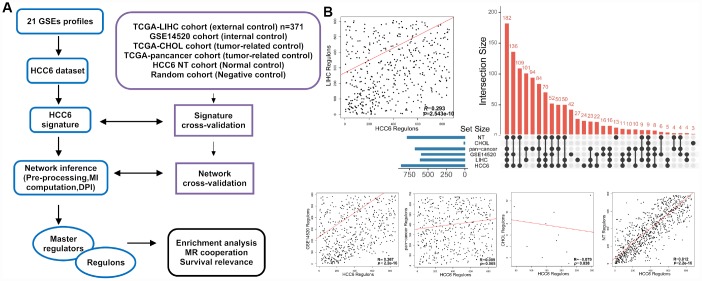
**Schematic representation of MR discovery and validation in HCC datasets.** (**A**) Different HCC-related gene expression datasets were analyzed in parallel with the ARACNe-MRA pipeline. (**B**) MRA agreement among different cohorts using unfiltered networks. The scatter plots show ranking agreement (by enrichment *P*-values) for all regulons between the different cohorts. Each dot represents one regulon (MR and all its targets) in the TN. The correlation coefficient *R* is given for each pairwise ranking. An UpSet plot of the intersection of identified regulons in different cohorts is presented in the right panel.

To assemble well-annotated human HCC gene expression profiles, 21 liver-oriented datasets were retrieved, yielding 1,316 gene expression profiles (Figure 1A). A detailed characterization of the HCC datasets is provided in Supplementary Table 1.

To ensure that the datasets generated from different types of arrays are comparable, the ComBat method [8], which is especially robust when handling multiple batches [9], was applied. For a more quantitative evaluation of the effects of dataset adjustment, principal variance component analysis (PVCA) [10] was performed to verify significant improvement after adjusting for intraplatform batch-to-batch differences (Supplementary Figure 1). In total, 12,100 annotated genes were present in the final dataset (named HCC6 hereafter).

### Functional enrichment analysis of HCC signatures

Based on identical normalization, filtration, and statistical treatment of raw datasets, the HCC6 signature was defined as a set of genes differentially expressed between tumor tissues (PT) and adjacent non-tumor tissues (NT). It consisted of 483 genes (32% of them upregulated) and included well-known HCC biomarkers (e.g. *GPC3, AFP, KPNA2*; Supplementary Table 2). We carried out gene ontology and KEGG pathway enrichment analysis to uncover potential biological functions for genes in the HCC6 signature, which were consistent with reprogramming features characteristic of cancer cells (Supplementary Figure 2).

In line with previous research [2], overrepresentation of cell proliferation markers was the most prominent feature in our HCC6 signature. We observed upregulation of numerous cell cycling genes, including cyclins (e.g. *CCNA2, CCNB1, CCNB2*), cyclin-dependent kinases and inhibitors (e.g. *CDK1, CDK4, CDKN3*), and genes acting on cell cycle and cell division checkpoints (e.g. *AURKA, NDC80*). Also, the HCC6 signature highlighted deregulation of genes associated with DNA replication (e.g. *MCM2-6, RFC4*), DNA unwinding (e.g. *TOP2A*), and DNA repair (e.g. *RRM2, FEN1*). Increased expression of several genes associated with protein translation, i.e. ribosomal subunits (e.g. *RPS5, RPL38*), and translation initiation factors (e.g. *EIF4G2*) was also observed. In addition, genes acting at the epigenetic level such as regulators of chromatin assembly and remodeling (e.g. *CHAF1A, HDAC1, HDAC5, HMGB2*), and components of the polycomb-repressive complex 2 (e.g. *EZH2, SUZ12*) were also up-regulated.

Metabolic reprogramming was noticeable for downregulated genes involved in liver specific metabolism, including those encoding acute phase plasma proteins (e.g. *A2M, ALB, CP*), components of complement and coagulation cascades (e.g. *C5-9, CFB, F2*), and detoxication enzymes (e.g. *ADH1A, CYP2E1*). Key regulators of oxidation-reduction processes (e.g. *STEAP3, CYP3A4, ALDH8A*) were also repressed.

### Regulon profiling in HCC

Next, a genome-wide transcriptional network (TN) centered on TFs and their predicted target genes was inferred using the RTN package [11, 12]. The computational pipeline is summarized in Figure 1A. Briefly, the TN was constructed by computing mutual information (MI) between annotated TFs [13] (n = 2,020 genes) and all potential targets in each cohort based on the ARACNe approach [14]. Then, the MRA algorithm was applied to compute the statistical significance of the overlap between the network and the molecular signature (differentially expressed genes). The groups of inferred target genes associated with each MR are hereinafter referred to as regulons.

To assess whether our results on the HCC6 dataset are consistent with other tumor-profiling datasets, we applied RTN on The Cancer Genome Atlas Liver Hepatocellular Carcinoma (TCGA-LIHC) data collection (external control), and on GSE14520 (internal control), etc, independently (Figure 1A). The enrichment *P*-value for each regulon was used to rank MRs identified in each network and the correlation statistic ® between these lists was compared.

We first ranked HCC6 regulons based on the enrichment score and found good agreement (*R* = 0.29-0.37) between HCC6, TCGA-LIHC, and GSE14520 (using its own differentially expressed genes as signature), suggesting that these HCC datasets share similar sets of regulated genes (Figure 1B). We then computed a TN for 990 tumor-matched, adjacent normal tissues from HCC6 patients. Enrichment for most MRs was found in this network, suggesting both PT and NT tissues share partial common regulatory network. Of note, after carrying out MRA on a network derived from a TCGA pan-cancer dataset, a lower level of correlation was observed (*R* = 0.09), while virtually no overlap was found between HCC6 and cholangiocarcinoma (TCGA-CHOL).

### Landscape of master regulators of HCC

**Figure 2:**
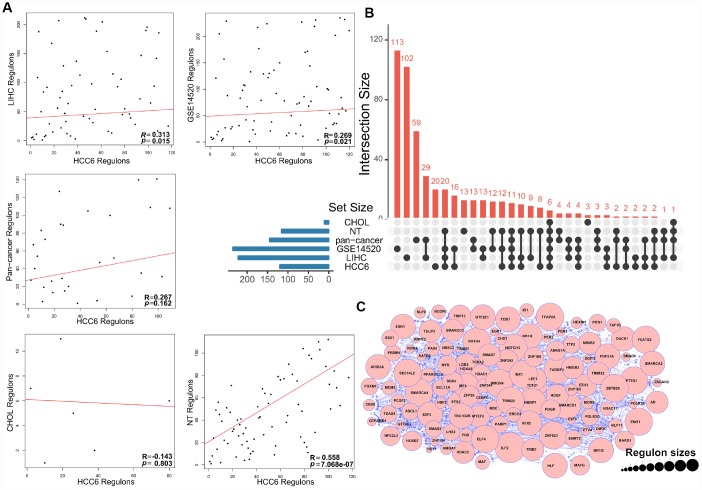
**MRA agreement among different cohorts using filtered networks.** (**A**) Networks were filtered by applying a DPI threshold of 0.01 to remove the weakest interactions. The scatter plots show ranking agreement (by enrichment *P*-value) for all regulons. (**B**) UpSet plot of the intersection of identified regulons in different cohorts. (**C**) Network visualization of the 120 MRs. The size of circles represents the size of each regulon.

To preserve the dominant TF-target pairs in the filtered TN, the weakest interactions between any two TFs and a common target gene were removed applying the data processing inequality (DPI) method. The analysis yielded 120 MR candidates in the HCC6 dataset (Supplementary Table 3). Again, there was good agreement between regulons in the HCC6, LIHC, and GSE14520 cohorts (Figure 2A, 2B). The regulatory network graph in Figure 2C shows association patterns between MRs in the HCC6 dataset.

### Validation of master regulators of HCC

**Figure 3:**
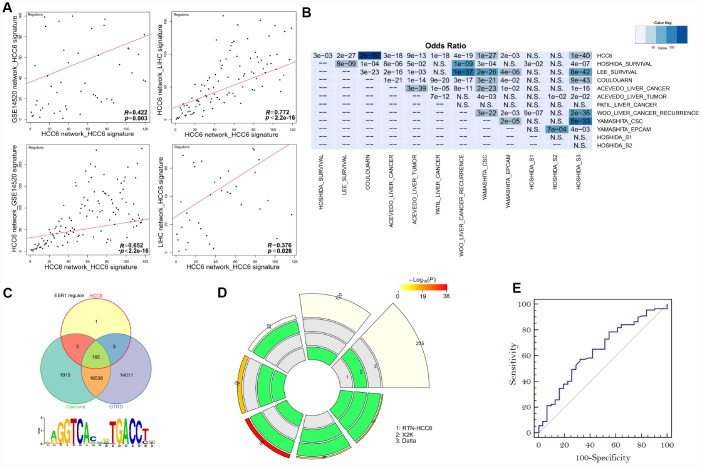
**Performance of MR prediction.** (**A**) MRs identified by using LIHC- and GSE14520-derived networks and the HCC6 signatures. The correlation coefficient *R* is given for each pairwise ranking. (**B**) Using HCC6 TN and different signatures, enrichment was estimated from the corresponding MRA analyses. The color key represents the odds ratios, and significant *P*-values are superimposed on the grids. (**C**) Venn diagrams showing overlap of the *ESR1* regulon with corresponding targets from Cistrome and GTRD. The *ESR1* motif was enriched in the *ESR1* regulon (Pscan analysis). (**D**) A circular plot visualizing all intersections and the corresponding statistics among three MR discovery methods. The three tracks in the middle represent the three methods, with individual blocks showing “presence” (green) or “absence” (grey) of the gene sets in each intersection. The height of the bars in the outer layer is proportional to the intersection size, as indicated by the numbers on the top of the bars. The color intensity of the bars represents the significance (*P* value) of the intersections. (**E**) ROC curves plotted for MR prediction.

We set out to systemically check the quality of predicted MRs. Since both network and signature might affect causal inferences during MR discovery, we performed cross-validation of MRA results using TNs and signatures from different HCC datasets. Compared with the regulons identified in the HCC6 network using different signatures, results showed the HCC6 based network had strong robustness (Figure 3A).

Aiming at assessing different features of liver cancer, various studies have reported molecular signatures related to HCC. Thus, we performed signature validation contrasting the TN derived from the HCC6 dataset with reported HCC-related signatures: progenitor tumor cell origin (CSC_Yamashita, EPCAM_Yamashita, CK19_Andersen [15], S2_Hoshida [16] and C2_Cario [17]), cellular proliferation [18], vascular invasion [19], TGF-beta_Coulouarn [20], MET_Kaposi-Novak [21], G3_Boyault [22], S1_Hoshida (TGFβ-WNT) [16]), Recurrence_Woo [23], OS_Kim [24], Interferon_Chiang [18], and G5/6_Boyault (CTTNB1_WNT activation) [22]. Results showed that the HCC6-signature covered almost all of these signatures (Figure 3B).

We also examined enrichment levels for target genes comprising the regulons of individual MRs. To this end, we collected recognized TF targets from the Cistrome Cancer web resource [25] and the Gene Transcription Regulation Database (GTRD) [26], both containing ChIP-seq derived processed data. As an example of this analysis, the *ESR1* regulon highly overlapped with the targets from both Cistrome and GTRD. In turn, the motif discovery tool Pscan [27] confirmed that the *ESR1* binding motif was enriched among its co-regulated genes (Figure 3C).

Besides ARACNe/RTN, we evaluated other methods designed for finding MRs in gene expression patterns, i.e. the Expression-2-Kinase (X2K) package with default parameters [28], and S. Datta’s approach [5]. Compared to these approaches, RTN identified more risk-MRs (Figure 3D).

Following a prototypic approach to validate cancer-associated MRs, we retrieved TFs annotated as cancer-related in at least one of three cancer gene databases (Bushman Laboratory cancer driver gene list [29], COSMIC somatic mutation catalog [30], and CCGD mouse cancer driver genes [31]). We found that 75% (90/120) of the MRs in our HCC6 gene set were annotated as cancer-associated in the above datasets. Applying information contained in these databases, receiver-operating characteristic curve analysis demonstrated the reliability of MR prediction (Figure 3E).

### Identification of MRs associated with HCC subtypes and etiology

**Figure 4:**
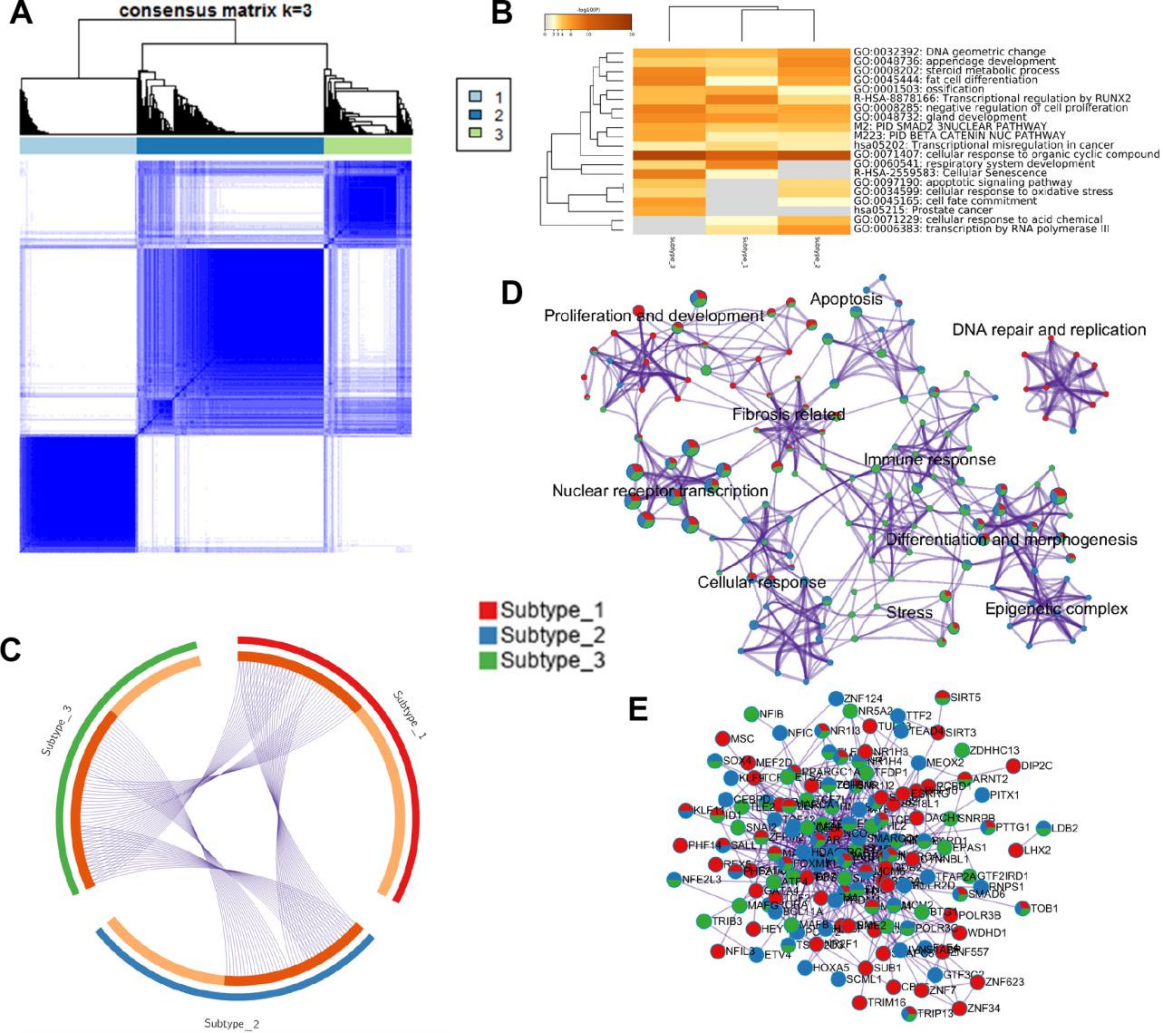
**HCC subtyping analysis and functional annotation of MRs.** (**A**) Three subgroups were identified by the non-negative matrix factorization (NMF) method in the HCC6 dataset, using consensus hierarchical clustering. (**B**) Meta-analysis of function annotation based on three subtype-related MR lists. Heatmap shows the top enrichment clusters (gray color indicates lack of significance). (**C**) Circos plot of MRs’ overlap among the three HCC subtypes. On the inside arc, dark orange color represents the MRs that appear in multiple subtypes and light orange color represents MRs that are unique to that subtype. (**D**) Enrichment network visualization for biological function from the three HCC subtypes. Nodes are represented by pie charts indicating their associations with each subtype. (**E**) Protein-protein interaction (PPI) network of MRs.

To answer the question of which MRs were associated with the different molecular subtypes of HCC, consensus hierarchical clustering [32] was performed on the HCC6 samples. Three tumor subgroups were discriminated from the consensus matrix (Figure 4A). Function annotation analyses (Figure 4B and 4D), MR overlap (Figure 4C), and protein-protein interaction (PPI) network of HCC6 MRs (Figure 4E) were further defined for the three HCC subtypes. For instance, network topology analysis indicated that HDAC1/2 were the top stress genes in the PPI network. As the epigenetic factors, HDACs control gene expression by recruiting multiple transcription factors and other chromatin-related factors. HDAC activate hepatocyte growth factor signaling in HCC [33].

We next performed MRA analysis within the HCC subtypes, interpreting subtype-related MRs using Metascape [34]. Associations with ‘cellular response to organic cyclic compound’, ‘nuclear receptor transcription pathway’, ‘chromatin organization’, ‘regulation of cell cycle process’, and ‘hormone- mediated signaling pathway’, among others, were defined for all three HCC subtypes (Supplementary Table 4). Subtype 1 was associated with biological processes such as ‘mitochondrial biogenesis’, ‘gland, mesenchyme, epithelium and tubule development’, ‘circadian clock’, and *FOXM1* and *NOTCH3* pathways, among others. Subtype 2, containing the BHC (BRAF-HDAC) and the CoREST-HDAC complexes, was linked to ‘cellular response to drug, and antibiotic’, ‘cellular glucose homeostasis’, ‘positive regulation of DNA repair’, ‘telomerase pathway’, and *AR* and *MYC* pathways, among others. Subtypes 1 and 2 both showed activated *AKT* signaling. Lastly, subtype 3 was defined by transcriptional networks related to ‘cellular response to oxygen levels’, ‘oncogene induced senescence’, ‘repression of *WNT* target genes’, ‘T-helper 17 type immune response’, ‘stem cell proliferation’, and ‘*TGF*-beta signaling pathway’, among others.

Since risk factors for developing HCC include HBV or HCV infection, alcoholic liver disease, and metabolism disorders like nonalcoholic steatohepatitis, we therefore tested whether the etiology of tumors was linked to candidate MRs. Detailed information on etiology-related MRs is provided in Supplementary Table 5.

### Consensus MRs in HCC

**Figure 5:**
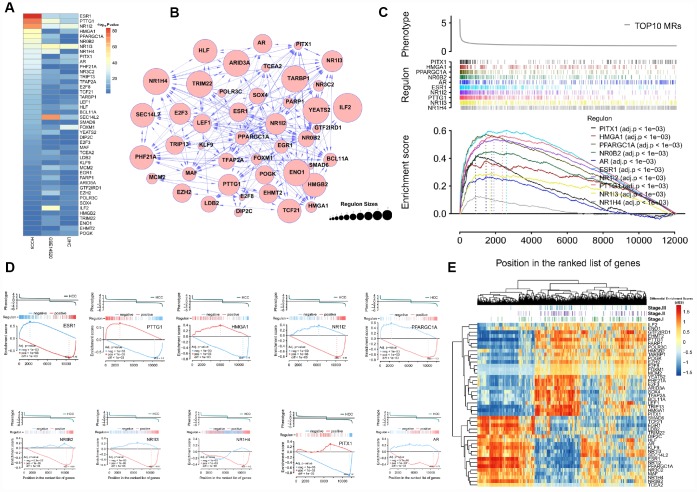
**Profiling of conserved MRs.** (**A**) Regulons are ranked by corresponding enrichment *P*-values, estimated for the HCC6, LIHC, and GSE14520 cohorts. (**B**) Network view of 44 consistent MRs. (**C**) GSEA of the top 10 MRs. (**D**) Examples of the top 10 MRs for which 2-tailed GSEA was carried out. (**E**) dES heatmap of the 44MRs.

To define a smaller set of conserved MRs, we selected the regulons showing significant enrichment across the HCC6, LIHC, and GSE14520 cohorts (Figure 5A). The association map of the resultant 44 MRs is provided in Figure 5B.

Once identified, we further tested the responsiveness of each regulon to tumor phenotype using gene set enrichment analysis (GSEA) [35]. Figure 5C shows that the top 10 regulons are consistently HCC-responsive. Regulons consist of both MR-induced and MR-repressed genes, and their relative activity influences the phenotype. To evaluate regulon activity, two-tailed GSEA was performed to calculate differential enrichment scores (dES) based on positively and negatively regulated regulon subsets in HCC samples. Detailed target information for each MR is provided in Supplementary Table 6.

Figure 5D presents an example of this analysis, showing GSEA running enrichment scores for the *ESR1* regulon after it was split into *ESR1*-activated and -repressed targets. Details on biological process involvement for the *ESR1* regulon, indicating multiple metabolic pathway inhibition mechanisms, are shown in Supplementary Figure 3. Figure 5E shows the dES profile of 44 MRs; two clusters resulted, suggesting opposite biological roles in HCC pathogenesis.

### Regulon activity and coordinated MRs as prognostic read-out

Next, dES representing regulon activity were used to investigate the prognostic value of MRs through Kaplan-Meier survival analysis. Almost half (20/44, *P* < 0.05) of the MRs in our HCC6 cohort were highly correlated with survival phenotype (Supplementary Table 7), compared with 14/44 and 32/44 in the GSE14520 and LIHC cohorts, respectively.

**Figure 6:**
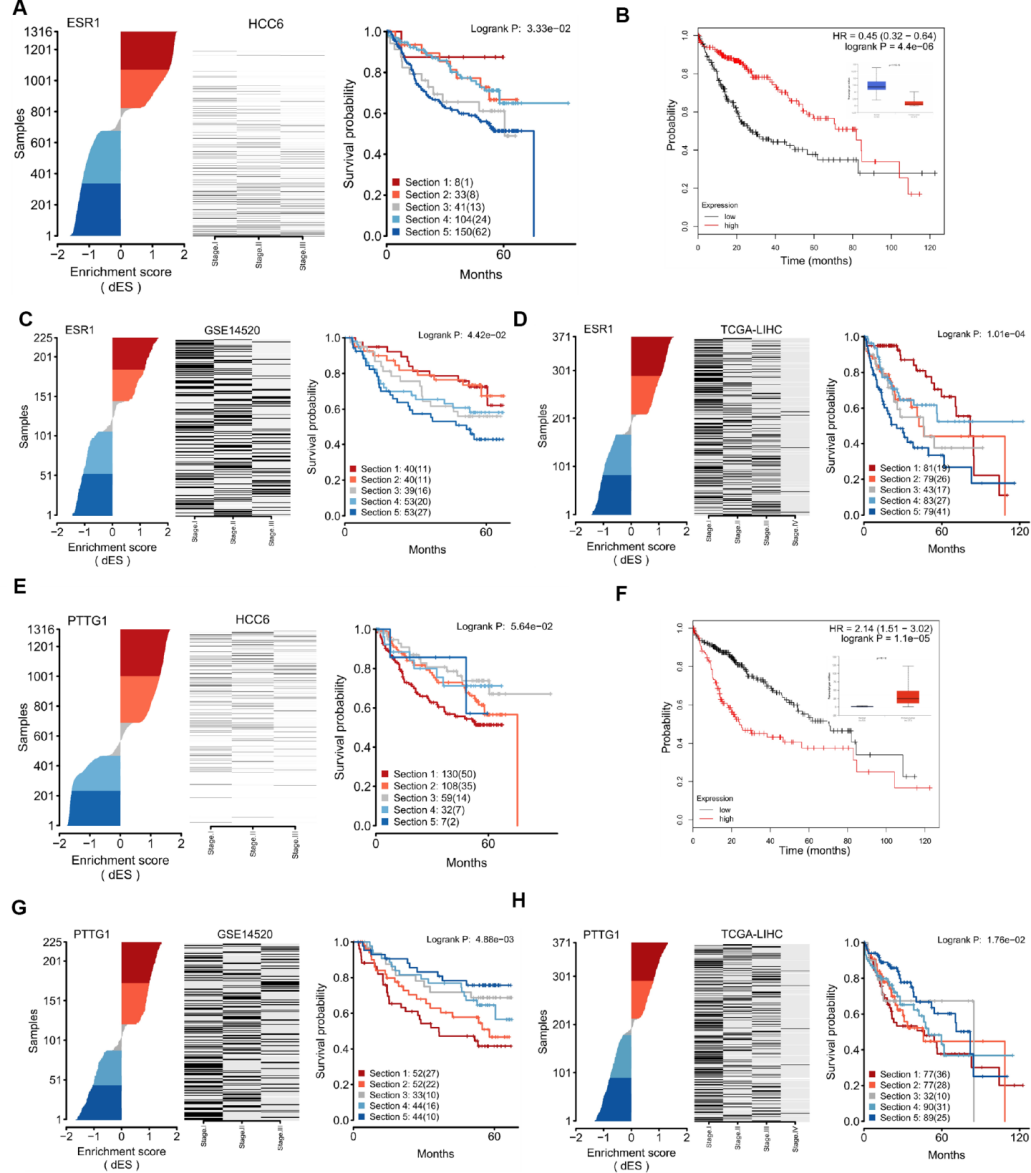
**Regulon activity as read-out of survival outcomes.** (**A**) dES of *ESR1* calculated for all samples in the HCC6 cohort. Disease-specific survival plots for each tumor subgroup are highlighted; patient numbers are listed for each section. (**B**) Kaplan-Meier survival curve using *ESR1* gene expression data in the LIHC cohort, generated using the KM-plotter tool [56]. (**C**) dES of *ESR1* in the GSE14520 cohort. (**D**) dES of *ESR1* in the LIHC cohort. (**E**–**H**) Analysis results for *PTTG1*.

Figure 6 shows dES and survival plots for the top 2 regulons. For the *ESR1* regulon, we found a continuous spectrum of dES across the tumors, except near the transition between its active and repressed state, which was characterized by an abrupt change. There was a strong trend for better survival in tumors with high dES. Significant trends were also noted after analyzing the GSE14520 and the LIHC cohorts, evaluated as controls (Figure 6C–6D). Upon stratification for *ESR1* expression only, this MR was strongly correlated with prognosis in the LIHC cohort (Figure 6B), but not in the GSE14520 cohort (Supplementary Table 7). Meanwhile, an opposite survival trend was found for the *PTTG1* regulon (Figure 6E–6H). These results suggest that regulon activity, expressed as dES, can predict survival outcome in a more context-dependent manner. Detailed MR-based survival analysis results are provided in Supplementary Figure 4.

**Figure 7:**
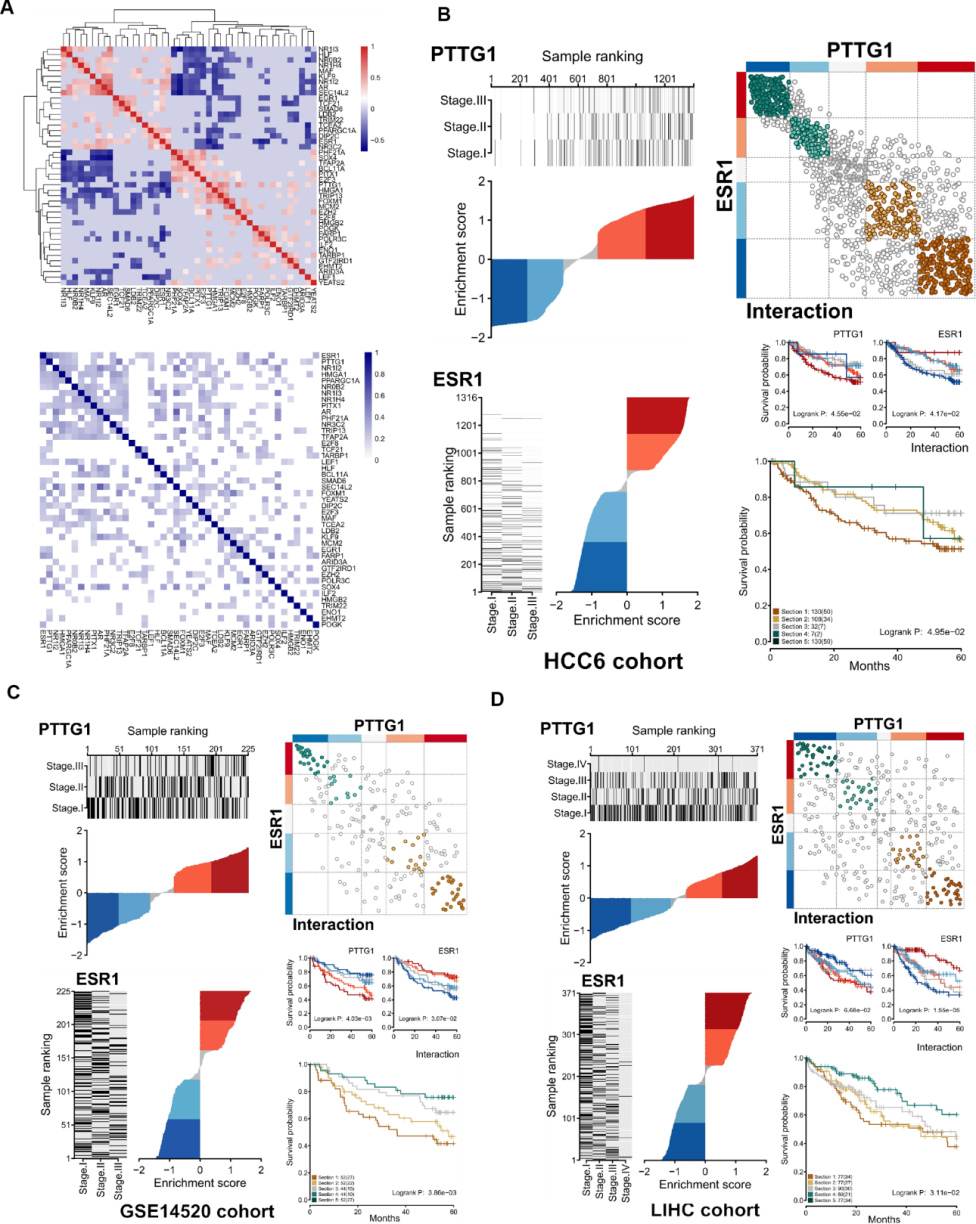
**Differential expression effects of MR interactions on shared target genes.** (**A**) Upper panel: Heatmap of gene expression correlation for targets shared by the 44 MRs (HCC6). Lower panel: Hierarchical clustering based on jaccard similarity coefficients (shades of blue) computed among 44 regulons. (**B**) Interaction of *ESR1*-*PTTG1* pair in the HCC6 cohort. The dES of *ESR1* and *PTTG1* are shown. On the right upper panel, a cartoon depicts the observed interactions between *ESR1* and *PTTG1* targets, with brown circles indicating co-activation, and green circles denoting co-repression. Targets are shown in grey if the two MRs have opposing effects. Assuming *ESR1* and *PTTG1* interaction, survival outcomes for the *ESR1* regulon, the *PTTG1* regulon, and both interacting regulons are depicted (**C**, **D**).

Since MRs can have divergent effects on the expression of a shared target gene in a real cellular setting, we examined the relationship between regulons. Correlation values were used to assess whether or not MR pairs regulated shared target genes in the same (positive or negative) direction. This analysis was carried out for 44 MRs in our regulatory network, and a correlation heatmap was generated. In addition, a heatmap of Jaccard similarity coefficient (JC) focusing on the overlap between the 44 regulons was also obtained (Figure 7A).

Generally, JC values fell into two distinct groups with high correlation within each group: gene targets shared between two MRs in the same group are regulated in the same direction by both MRs, whereas gene targets shared between a MR in one group and a MR in the other are regulated in opposite directions. This suggests the existence of two distinct regulatory MR groups, each one opposing the effects of the other. Again, using *ESR1* and *PTTG1* as an example, these two MRs were highly anti-correlated and showed extensive overlap (*R* = -0.586, JC = 0.431). Considering compounding effect, stratification of activity of interacted-ESR1 and PTTG1 regulon further reveals different survival patterns. This influence was verified in the LIHC and GSE14250 cohorts (Figure 7C–7D).

### Functional analysis of a candidate MR

**Figure 8:**
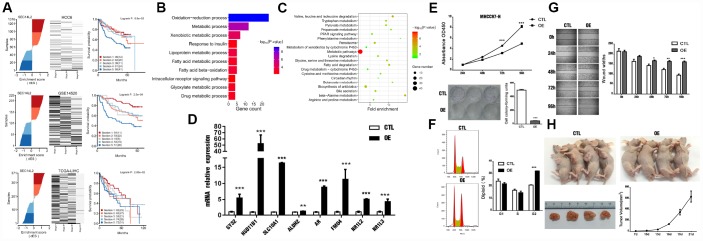
***SEC14L2* is a potential HCC suppressor.** (**A**) Calculation of dES and survival responses for *SEC14L2* in the HCC6 cohort. (**B**) GO terms enrichment analysis of *SEC14L2* regulon. (**C**) KEGG pathway enrichment analysis of *SEC14L2* regulon. (**D**) Representative target genes of *SEC14L2* regulon were significantly up-regulated after *SEC14L2* overexpression. (**E**) *SEC14L2* overexpression significantly inhibited cell proliferation (CCK-8 assay) and colony formation in HCC cell lines. Upper and lower panels show corresponding results in MHCC97-H cells. These assays were repeated in Huh7 cells (Supplementary Figure 6). (**F**) Cell-cycle distribution analysis of MHCC97-H cells overexpressing *SEC14L2*. (**G**) Cell migration (wound-healing) assay results. Data represent the average of three independent experiments in duplicate. (**H**) Effect of *SEC14L2* overexpression on tumor xenografts in vivo. Almost total inhibition of tumor growth was observed after subcutaneous implantation of *SEC14L2-* overexpressing MHCC97-H cells in nude mice. OE, over-expressed; *P < 0.05, **P < 0.01, ***P < 0.001.

The lipid-binding protein *SEC14L2*, which possesses putative transcriptional activatory activity, was predicted as a conservative MR in HCC in this study (Figure 8A). Functional enrichment analysis indicated that oxidation-reduction process, metabolic process, PPAR signaling, peroxisome pathway, and fatty acid degradation, etc, were significantly regulated by *SEC14L2* regulon in HCC (Figure 8B, 8C).

A recent report identified *SEC14L2* as a host factor permitting replication of clinical HCV isolates [36], but the relationship between this molecule and HCC was not established. Notably, *SEC14L2* expression could not be detected in human hepatoma and non-hepatoma cell lines in vitro [36]. However, primary human hepatocytes, both from fetal and adult sources, expressed readily detectable levels [36]. We thus examined the potential growth-suppressive effects of *SEC14L2* re-expression in HCC cells.

Restored expression of *SEC14L2* was observed in *SEC14L2*-transduced MHCC97-H cells, which showed reduced proliferation and decreased colony formation (Figure 8E and Supplementary Figure 5). Also, the number of cells in G2 phase following *SEC14L2* ectopic expression was substantially increased (50.96 ± 0.20% *vs* 43.28 ± 0.95% in control cells; *P* < 0.05). On wound healing assays, ectopic expression of *SEC14L2* distinctly inhibited migration of MHCC97-H cells (*P* < 0.01) (Figure 8G).

We subsequently investigated the effects of forced *SEC14L2* expression on the tumorigenic potential of HCC cells in vivo. To this end, MHCC97-H cells stably transduced with *SEC14L2* or empty vector were injected subcutaneously into nude mice. Periodic volume measurements showed that tumor growth was nearly abolished after *SEC14L2*-transduction, compared with tumors containing control cells (Figure 8H).

As expected, expression of the component genes like *GYS2, HSD11B1, SLC10A1, ALDH2*, and *AR*, etc, in *SEC14L2* regulon were significantly up-regulated after the ectopic expression of *SEC14L2* in MHCC-97H cells (Figure 8D). Briefly, *GYS2* was recently found to be responsible for the deregulation of glycogen metabolism in HCC [37]. *HSD11B1* was identified as a circulating biomarker candidate for HCC [38]. *SLC10A1*(*NTCP*) expression was markedly reduced in most HCC [39]. *ALDH2* deficiency promotes liver cancer by activating oncogenic pathways via oxidized DNA-enriched extracellular vesicles [40]. These results suggested that *SEC14L2* regulon might have a positive mechanism in metabolic pathways regulated by *SEC14L2*.

## DISCUSSION

Streamlining the analysis of multiple datasets using the same computational processes enhances statistical power and may significantly increase the accuracy of the findings [41, 42]. Our study compiled a large HCC dataset (HCC6) and verified its quality by PVCA analysis. Importantly, we show that the HCC6 signature panel overlaps extensively with a comprehensive 935-gene HCC signature reported by Allain et al. in a meta-analysis of multiple datasets [2]. This lends confidence that the HCC6 dataset captured the main features of HCC.

Several lines of evidence support the relevance of the MRs and associated regulons discovered in the present study. First, two of the most important parameters in MRA, i.e. transcription networks and gene signatures, were widely cross-tested. Upon similar analysis, MRs consistently identified from the HCC6 networks were absent in networks obtained from other malignancies. Second, most MRs identified in this work are cancer-related according to database annotation. Finally, the impact of some MRs on liver cancer predicted by HCC6 is highly consistent with current knowledge.

In this sense, the relation between *ESR1* and malignant disease has been asserted in a variety of tissues including breast, colon, bladder, and liver. As a candidate tumor suppressor gene [43], decreased *ESR1* expression was significantly correlated to high liver damage score, pathological invasion, and tumor size [44]. *PTTG1*, a human securin that inhibits sister chromatid separation and is involved in transformation and tumorigenesis, is overexpressed in HCC and has prognostic significance for postoperative survival of patients with HCC [45]. Our prediction also retrieved well-known MRs such as *FOXM1, EZH2*, and *SOX4* [6]. *FOXM1*, cooperating with YAP, was found to contribute to chromosome instability in liver cancer [46].

Besides reaffirming findings from previous studies (Supplementary Table 8), our analysis also predicted putative MR functions in HCC. For instance, several MRs belonging to the orphan nuclear receptor (NR) family such as NR0B2, NR1I2, NR1H4, and NR1I3 were found.

NR0B2 is a transcriptional corepressor affecting diverse metabolic processes, including bile acid synthesis, cholesterol and lipid metabolism, and glucose and energy homeostasis. Evidence suggests that NR0B2 plays an important suppressing role in the development of liver cancer [47]. Research has revealed broader transcriptional circuits controlled by NR1H4. NR1H4 deficiency led to a 100% incidence of spontaneous liver tumors in male and female mice, indicating that disruption of estrogen-protected pathways promotes hepatic oncogenesis. NR1I2 plays an integral role in xenobiotic and endobiotic metabolism, glucocorticoid and mineralocorticoid homeostasis, vitamin metabolism, and hepatic gluconeogenesis, and was shown to promote tumor growth and chemo-resistance in major cancer types [48]. NR1I3 regulates a set of genes involved in cellular growth, and studies have shown that it may also aid in the promotion of tumor formation. Thus, our prediction suggests that multiple NRs might also be HCC-related MRs. Broader liver transcriptional circuits controlled by multiple orphan NRs warrant further consideration.

Overall, we found that 90 out of 120 MRs analyzed have support from cancer-gene database. As for the rest, little is known about their role in liver cancer according to current literature. For example, no study has so far implicated *SEC14L2* in liver cancer, though proteomic analysis identified low levels of *SEC14L2* to be prognostic markers for overall breast cancer survival [49]. Saeed et al*.* identified *SEC14L2* as a host factor permitting replication of clinical HCV isolates in cell culture [36]. We found that low *SEC14L2* expression is associated with poor patient survival in the HCC6, LIHC, and GSE14520 cohorts. Moreover, *SEC14L2* cross-talks with many NRs (Supplementary Figure 5). Further support for the importance of *SEC14L2* comes from our functional in vivo experiment, indicating that restoration of *SEC14L2* expression in HCC cells significantly inhibited tumor growth.

Earlier, three sub-clusters were defined in HCC by the TCGA research team [50]. Clustering analysis of HCC6 transcriptome profiles also resulted in three clusters, and subtype-related MRs were predicted therein. It would be interesting to investigate the functions of these MRs in future studies.

Importantly, a significant correlation was observed between the regulon activity of various MRs and patient survival. In contrast, simple classification based on molecular expression levels did not unmask prognostic relevance across multiple datasets. For instance, *ESR1* and *PTTG1* do not have significant prognostic value in the GSE14520 cohort, though these two genes have a clear role in HCC. Therefore, MRs might have increased power to detect prognosis traits that are otherwise concealed by sample heterogeneity and co-regulated genes. The fact that MRs could predict clinical outcome, while MR pairs were differentially linked to opposite prognostic categories, prompted us to deduce that MR interactions have a significant, probably major, contribution to HCC pathogenesis. These findings may reconcile controversies on the prognostic importance of HCC biomarkers and devise an applicable way to subgroup patients based on MR interactions.

In summary, our integrative analysis led to the discovery of many MRs with putative roles in HCC development and progression. Further, the present study offers information to define MRs as biomarkers for early stage diagnosis and to direct targeted therapeutics for HCC.

## MATERIALS AND METHODS

### HCC gene expression compendium

The HCC compendium was constructed by collecting 21 HCC-oriented datasets from six main microarray platforms (GPL570, GPL571, GPL3921, GPL96, GPL6244, and GPL10558) in Gene Expression Omnibus (https://www.ncbi.nlm.nih.gov/geo/) (i.e., GPL570:GSE1898 and GSE4024, GPL571/3921:GSE14520, etc.). All the datasets used fresh frozen liver tissues.

Raw data were uniformly aggregated and normalized using robust multi-array average (RMA) [51] or lumi package [52] according to the different platform. To ensure that the datasets generated from the six types of arrays are comparable, the ComBat batch correction method was performed to remove platform-specific effects [8]. Finally, 2,306 HCC gene expression profiles, including 1,316 tumor tissues (PT) and 990 adjacent non-tumor tissues (NT), were compiled. Genes with multiple probesets were represented by the mean intensity across all samples. RNA-sequencing liver cancer (TCGA-LIHC) Level 3 gene expression data and clinical information were downloaded from the Cancer Genome Atlas data portal.

### HCC gene signature

Differentially expressed genes between PT and NT were identified by limma package. FDR adjusted *P* < 0.05 and an absolute fold change > 1.5 were used as cut-offs for analysis. Enrichment for biological functions or canonical pathways was assessed using DAVID [53] or Metascape [34]. ConsensusClusterPlus (CHC) was used to carry out consensus hierarchical clustering to identify subtypes [32], and consensus indices of each pair of samples were visualized in a consensus matrix.

### Network inference and MRA analysis

R package RTN [11, 12] was used to reconstruct and analyze the regulatory network based on mutual information (MI), a measure that evaluates dependencies between two random variables. Briefly, the regulatory structure of the network is derived by mapping significant associations between a known TF and its potential targets. Interactions below a minimum MI threshold are eliminated by a permutation step and unstable interactions are further removed by bootstrap to create a consensus network. In a last step, the data processing inequality (DPI) algorithm is applied with null tolerance to eliminate interactions that are likely to be mediated by another TF. The RedeR package [54] was used to visualize the network.

After network inference, master regulator analysis (MRA) was performed. The algorithm computes the statistical significance of the overlap between the regulon and the signatures (differentially expressed genes obtained from each study), corrected for multiple comparisons.

### Regulon activity and survival analysis

Two-tailed gene set enrichment analysis (GSEA) was performed to calculate regulon activity with 1000 permutations, as previously described [11, 12]. Briefly, the resultant regulon was split into two subgroups, positive targets (A) and negative targets (B), using Pearson correlation. Next, independent enrichment scores (ES) for each subgroup were tested by GSEA statistics in the ranked phenotype, with two enrichment distributions. Regulon activity, represented by differential enrichment score (dES=ES_A_-ES_B_), was thus computed. A highly positive dES implies that the regulon is induced, while a highly negative dES indicates that the regulatory unit is repressed, in the disease phenotype. The two-tail GSEA *P*-value cutoff was set to 0.05 and 1000 permutations were used.

Survival analysis was performed using log-rank statistics. For stratified tests, patients were divided into three groups based on dES values: those with an active regulon (dES>0 and ES_A_>0 and ES_B_<0), those with a repressed regulon (dES<0 and ES_A_<0 and ES_B_>0), and a small group in which the dES values were around zero (inconclusive). The two large groups were further subdivided in half.

### Cell culture

The liver cancer cell lines Huh7 and MHCC97-H were obtained from the Cell Bank of Chinese Academy of Sciences (Shanghai, China), and have been authenticated by STR DNA profiling. Cells were grown in Dulbecco's modified Eagle's medium (DMEM) supplemented with 100U/ml penicillin, 100μg/ml streptomycin and 10% fetal bovine serum. Cultures were performed at 37°C in a 5% CO_2_ atmosphere.

### Cell transduction

Lentivirus production in HEK293T cells was performed as previously described [55]. MHCC97-H cells were infected with lentivirus expressing *SEC14L2* at a multiplicity of infection (M.O.I.) of 5 in the presence of 10 μg/ml polybrene (Sigma, USA) for 16 h, and selected using 2 μg/ml of puromycin (Sigma, USA) for one week. Stable control and *SEC14L2*-overexpressing MHCC97-H cells were obtained after verification of *SEC14L2* expression by qPCR.

### Cell proliferation assays

Cell proliferation was detected with CCK8 reagents (Dojindo, Japan). To evaluate colony formation, cells infected with lentivirus were cultured for 3 weeks, stained with gentian violet, and colonies with >50 cells were counted. Cell cycle distribution was analyzed using a cell cycle staining kit (Multisciences Biotech Co., China) and flow cytometry. The experiments were repeated at least three times.

### In vivo tumorigenicity

Male athymic nude mice (4-5 weeks old) were purchased from Shanghai Laboratory Animal Co. Ltd (SLAC, China). Mice were injected subcutaneously with 5 × 10^6^ MHCC97-H cells in 0.1 mL of serum-free DMEM. The left flank was implanted with control tumor cells whereas the right side was injected with *SEC14L2*-transduced tumor cells. Animal procedures were approved by Hangzhou Normal University’s Animal Care and Use Committee (Hangzhou, China).

### Statistics

Bioinformatics analysis of microarray data was carried out in R (version 3.4.1) and statistical analysis of experimental results was performed using Prism GraphPad software (version 7). Unpaired Student’s t-test was used to compute statistical significance, set at p < 0.05 unless specified. Data are presented as mean ± SEM.

## Supplementary Material

Supplementary Figures

Supplementary Tables

Supplementary Table 2

Supplementary Table 3

Supplementary Table 4

Supplementary Table 5

Supplementary Table 6

Supplementary Table 7

Supplementary Table 8
