# Supplementary material for: Discovering master regulators in hepatocellular carcinoma: one novel MR, SEC14L2 inhibits cancer cells
Source: Aging (Albany NY). 2019 Dec 18;11(24):12375–411. doi: 10.18632/aging.102579 (PMC6949064; doi:10.18632/aging.102579)
Supplement: Supplementary Figures [file aging-11-102579-s009..pdf]

SUPPLEMENTARY FIGURES

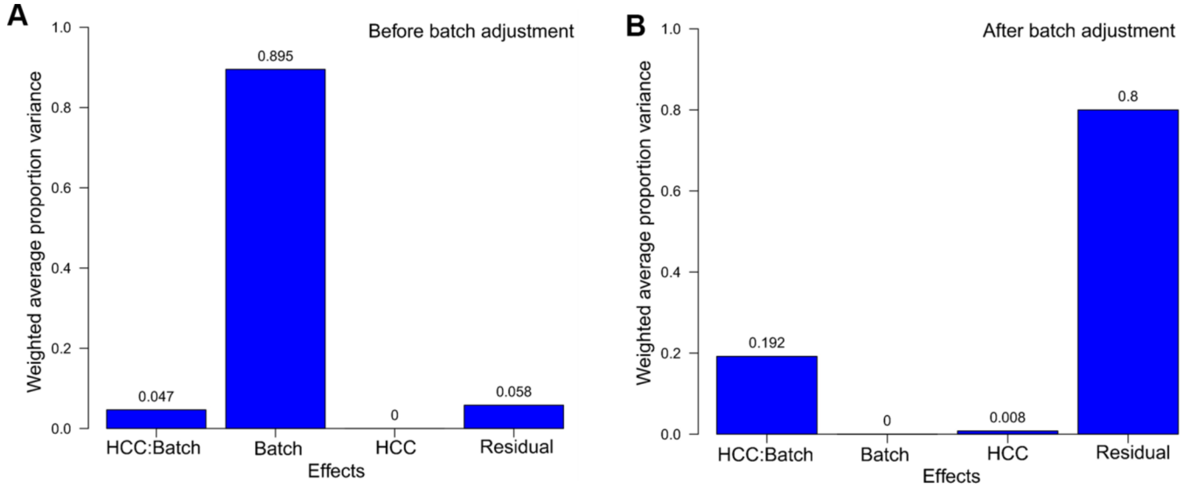

Supplementary Figure 1. Quality assessment of batch correction for microarray data merging. The bar chart shows the proportion of variation attributable to batch effects introduced from potential sources. All the effects, including batch and profile effects, interaction between batch and profile effects, and residual effects, were estimated for their contribution to the overall variation by PVCA. (**A**) Data before batch adjustment. (**B**) Data processed by ComBat as batch adjustment model.

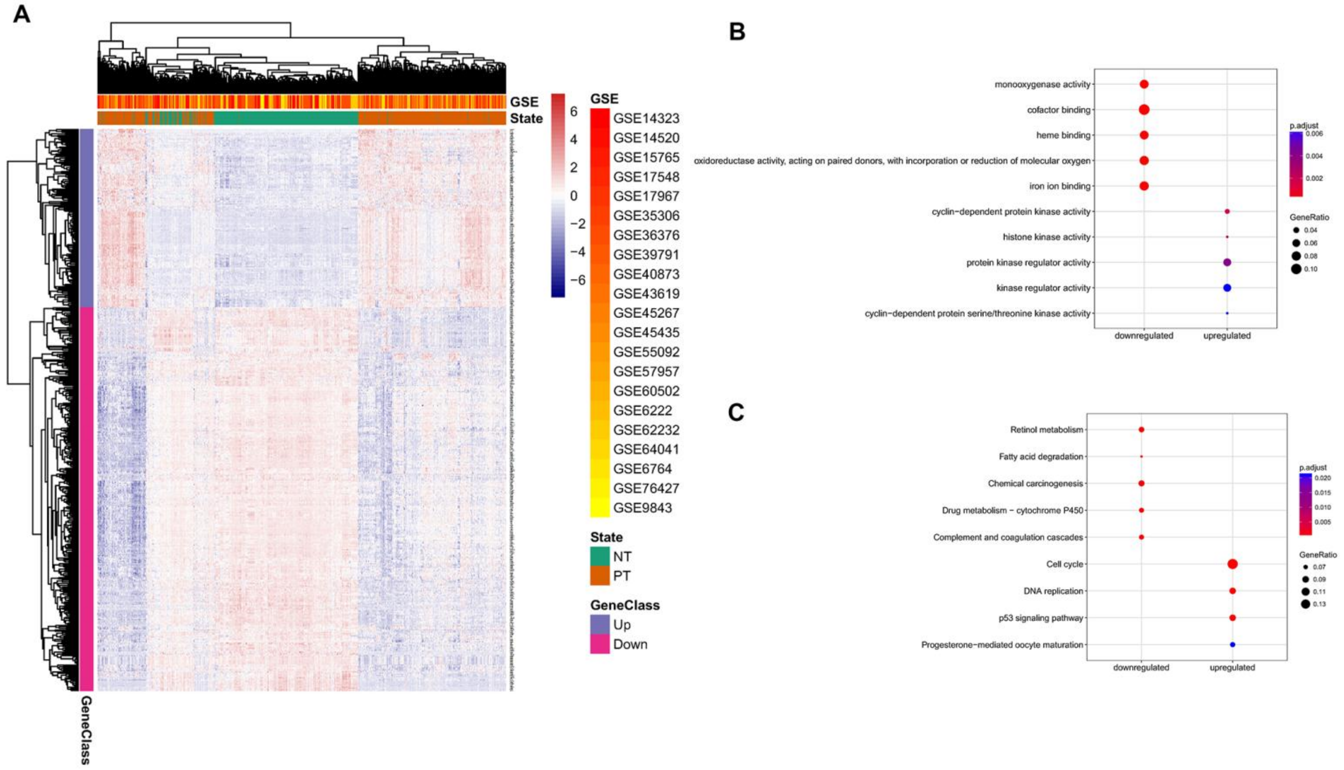

Supplementary Figure 2. HCC6 signature. (**A**) Clustering analysis based on expression data from 21 HCC-related datasets revealed a clear transcriptional homogeneity in the investigated samples. (**B**) GO and KEGG analysis of the HCC6 signature.

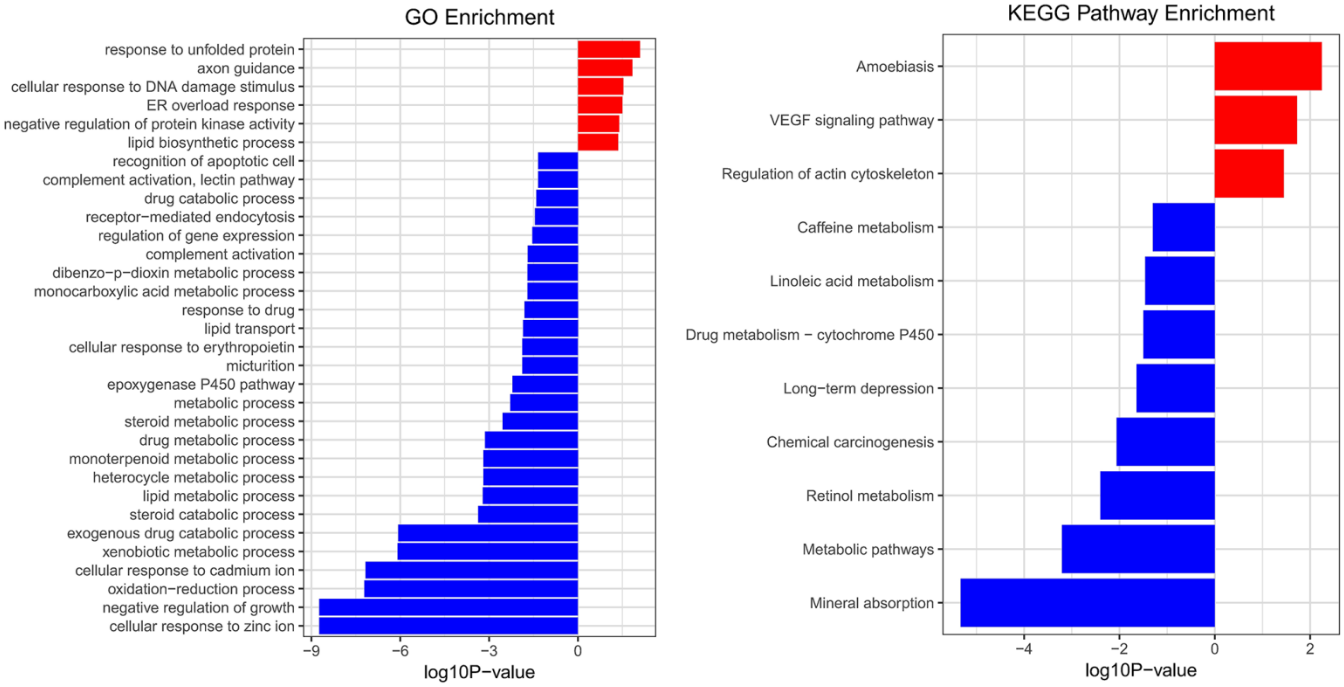

Supplementary Figure 3. Functional annotation of the ESR1 regulon. Red indicates the activated functions by ESR1 regulon. Blue indicates the repressed functions by ESR1 regulon.

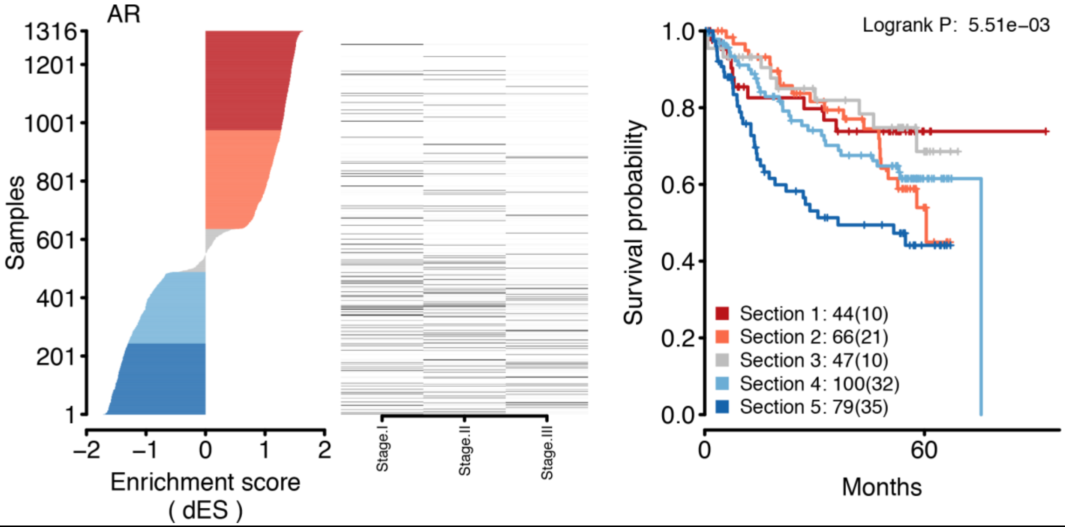

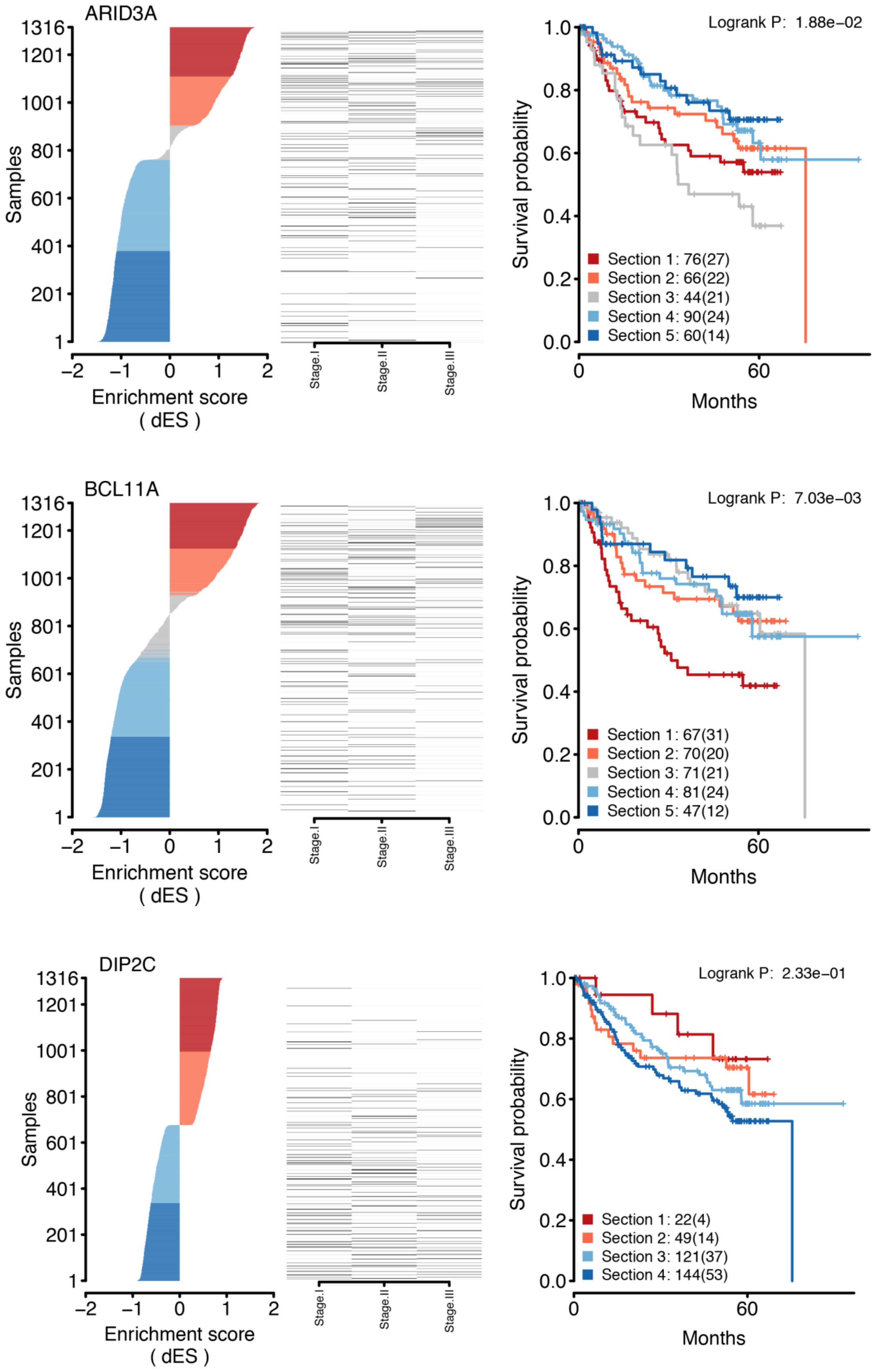

ARID3A
Samples
Enrichment score (dES)
Stage.I
Stage.II
Stage.III
Logrank P: 1.88e−02
Survival probability
Months
Section 1: 76(27)
Section 2: 66(22)
Section 3: 44(21)
Section 4: 90(24)
Section 5: 60(14)
BCL11A
Samples
Enrichment score (dES)
Stage.I
Stage.II
Stage.III
Logrank P: 7.03e−03
Survival probability
Months
Section 1: 67(31)
Section 2: 70(20)
Section 3: 71(21)
Section 4: 81(24)
Section 5: 47(12)
DIP2C
Samples
Enrichment score (dES)
Stage.I
Stage.II
Stage.III
Logrank P: 2.33e−01
Survival probability
Months
Section 1: 22(4)
Section 2: 49(14)
Section 3: 121(37)
Section 4: 144(53)

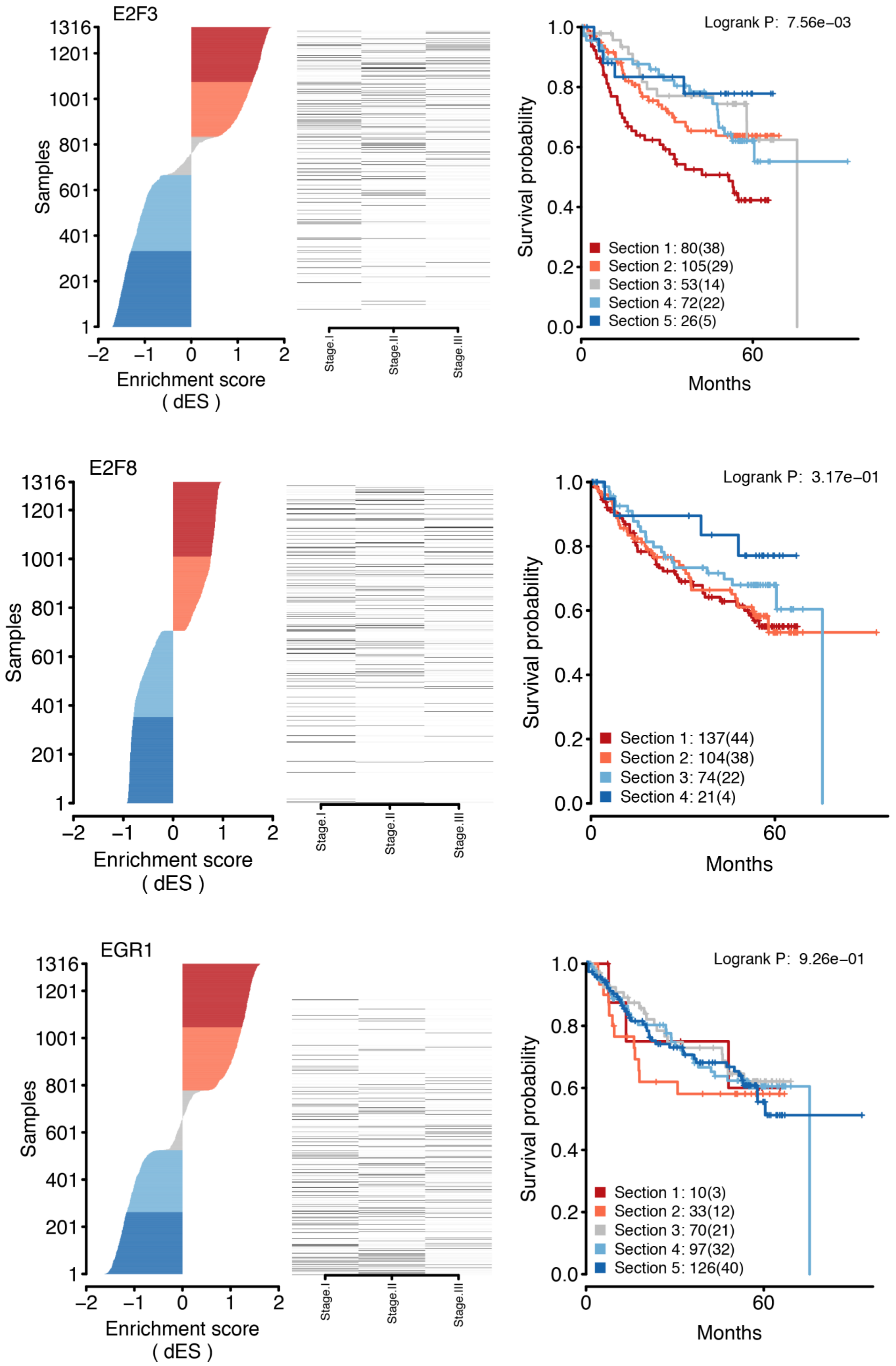
E2F3
Samples
Enrichment score
(dES)
Stage.I
Stage.II
Stage.III
Logrank P: 7.56e−03
Survival probability
Section 1: 80(38)
Section 2: 105(29)
Section 3: 53(14)
Section 4: 72(22)
Section 5: 26(5)
Months
E2F8
Samples
Enrichment score
(dES)
Stage.I
Stage.II
Stage.III
Logrank P: 3.17e−01
Survival probability
Section 1: 137(44)
Section 2: 104(38)
Section 3: 74(22)
Section 4: 21(4)
Months
EGR1
Samples
Enrichment score
(dES)
Stage.I
Stage.II
Stage.III
Logrank P: 9.26e−01
Survival probability
Section 1: 10(3)
Section 2: 33(12)
Section 3: 70(21)
Section 4: 97(32)
Section 5: 126(40)
Months

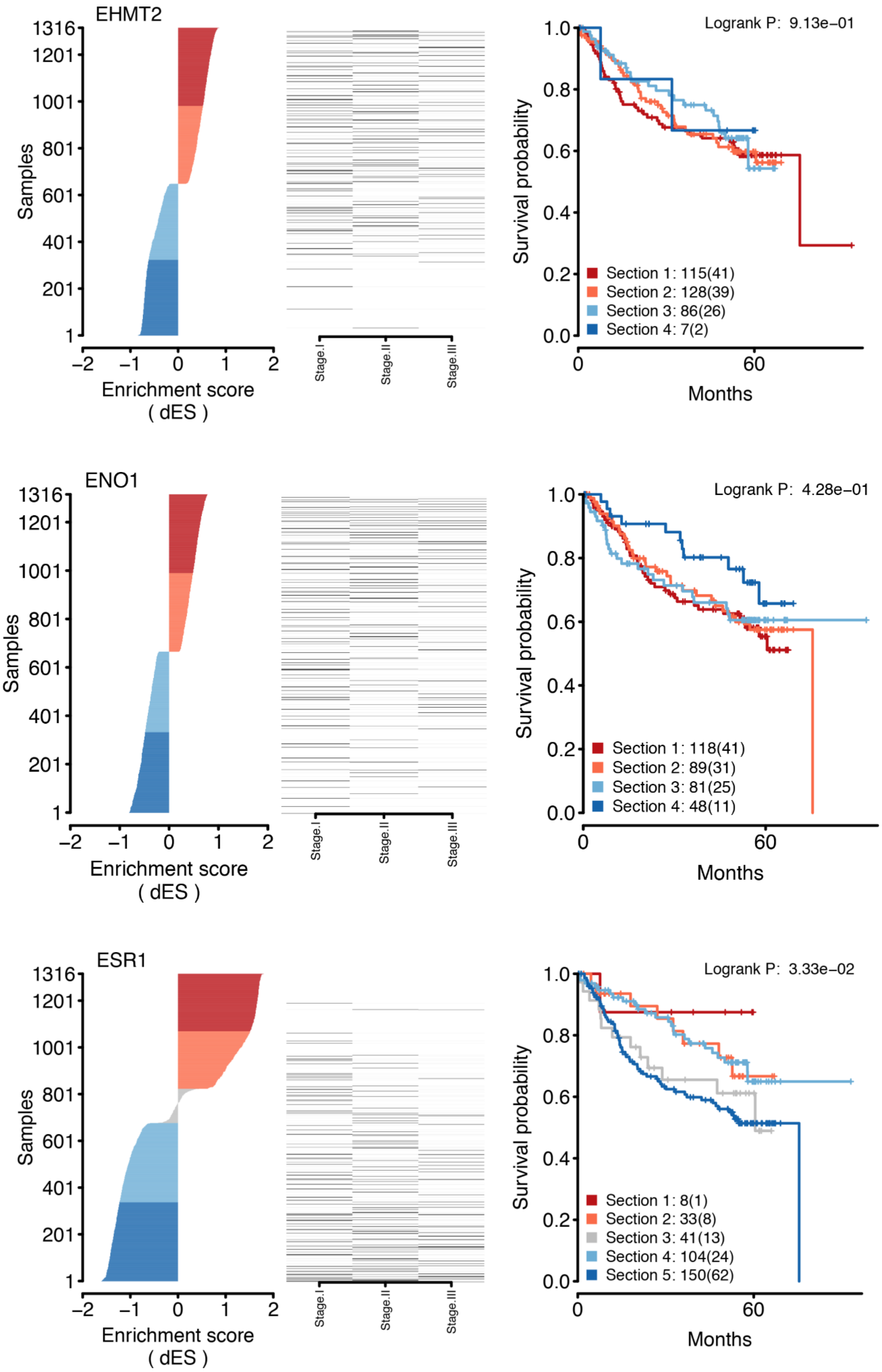
EHMT2
1316
1201
1001
801
601
401
201
1
Samples
-2 -1 0 1 2
Enrichment score
(dES)
Stage.I
Stage.II
Stage.III
Logrank P: 9.13e-01
1.0
0.8
0.6
0.4
0.2
0.0
Survival probability
Section 1: 115(41)
Section 2: 128(39)
Section 3: 86(26)
Section 4: 7(2)
0 60
Months
ENO1
1316
1201
1001
801
601
401
201
1
Samples
-2 -1 0 1 2
Enrichment score
(dES)
Stage.I
Stage.II
Stage.III
Logrank P: 4.28e-01
1.0
0.8
0.6
0.4
0.2
0.0
Survival probability
Section 1: 118(41)
Section 2: 89(31)
Section 3: 81(25)
Section 4: 48(11)
0 60
Months
ESR1
1316
1201
1001
801
601
401
201
1
Samples
-2 -1 0 1 2
Enrichment score
(dES)
Stage.I
Stage.II
Stage.III
Logrank P: 3.33e-02
1.0
0.8
0.6
0.4
0.2
0.0
Survival probability
Section 1: 8(1)
Section 2: 33(8)
Section 3: 41(13)
Section 4: 104(24)
Section 5: 150(62)
0 60
Months

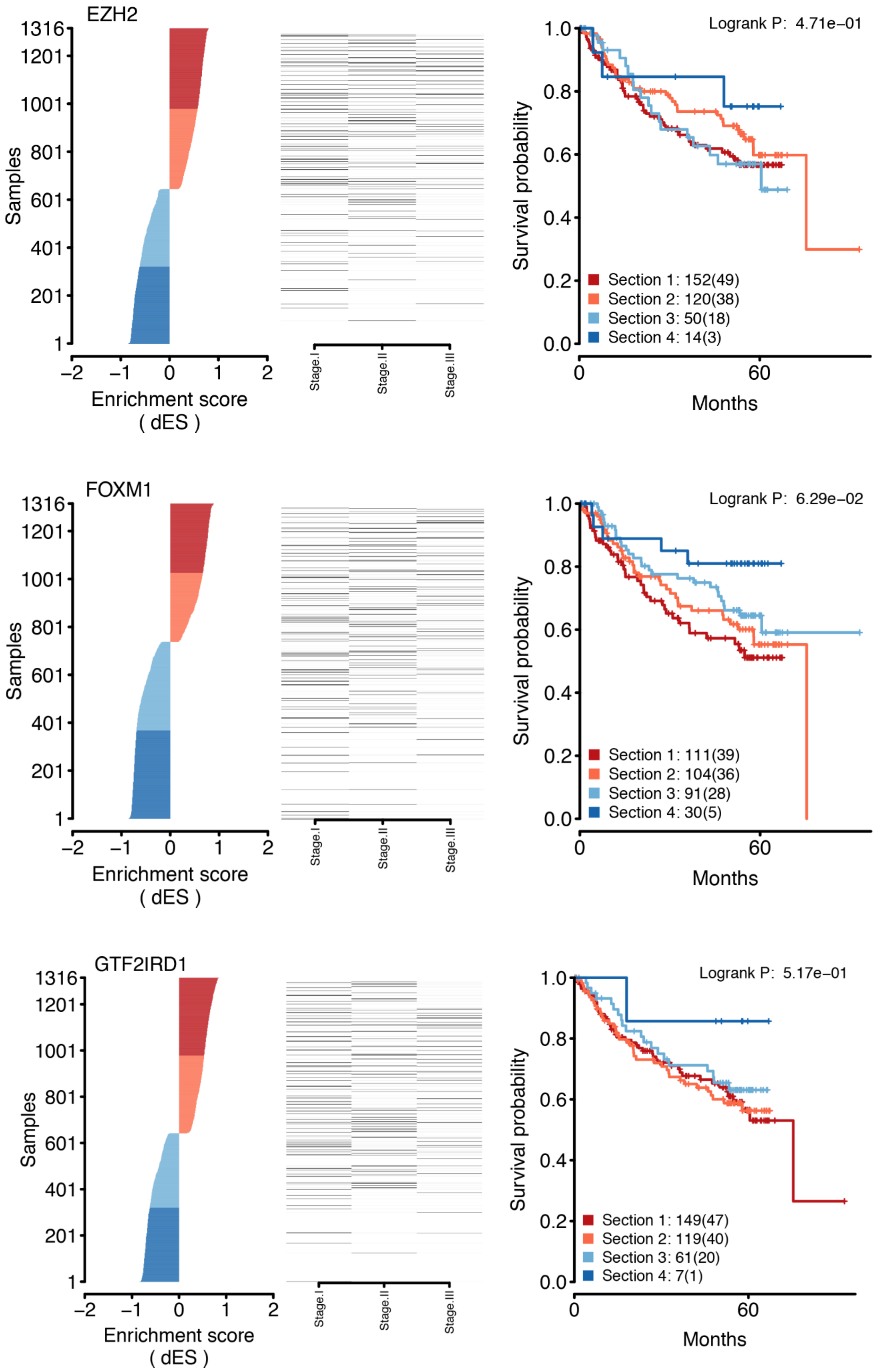
EZH2
Samples
Enrichment score
(dES)
Stage.I
Stage.II
Stage.III
Logrank P: 4.71e−01
Survival probability
Months
Section 1: 152(49)
Section 2: 120(38)
Section 3: 50(18)
Section 4: 14(3)
FOXM1
Samples
Enrichment score
(dES)
Stage.I
Stage.II
Stage.III
Logrank P: 6.29e−02
Survival probability
Months
Section 1: 111(39)
Section 2: 104(36)
Section 3: 91(28)
Section 4: 30(5)
GTF2IRD1
Samples
Enrichment score
(dES)
Stage.I
Stage.II
Stage.III
Logrank P: 5.17e−01
Survival probability
Months
Section 1: 149(47)
Section 2: 119(40)
Section 3: 61(20)
Section 4: 7(1)

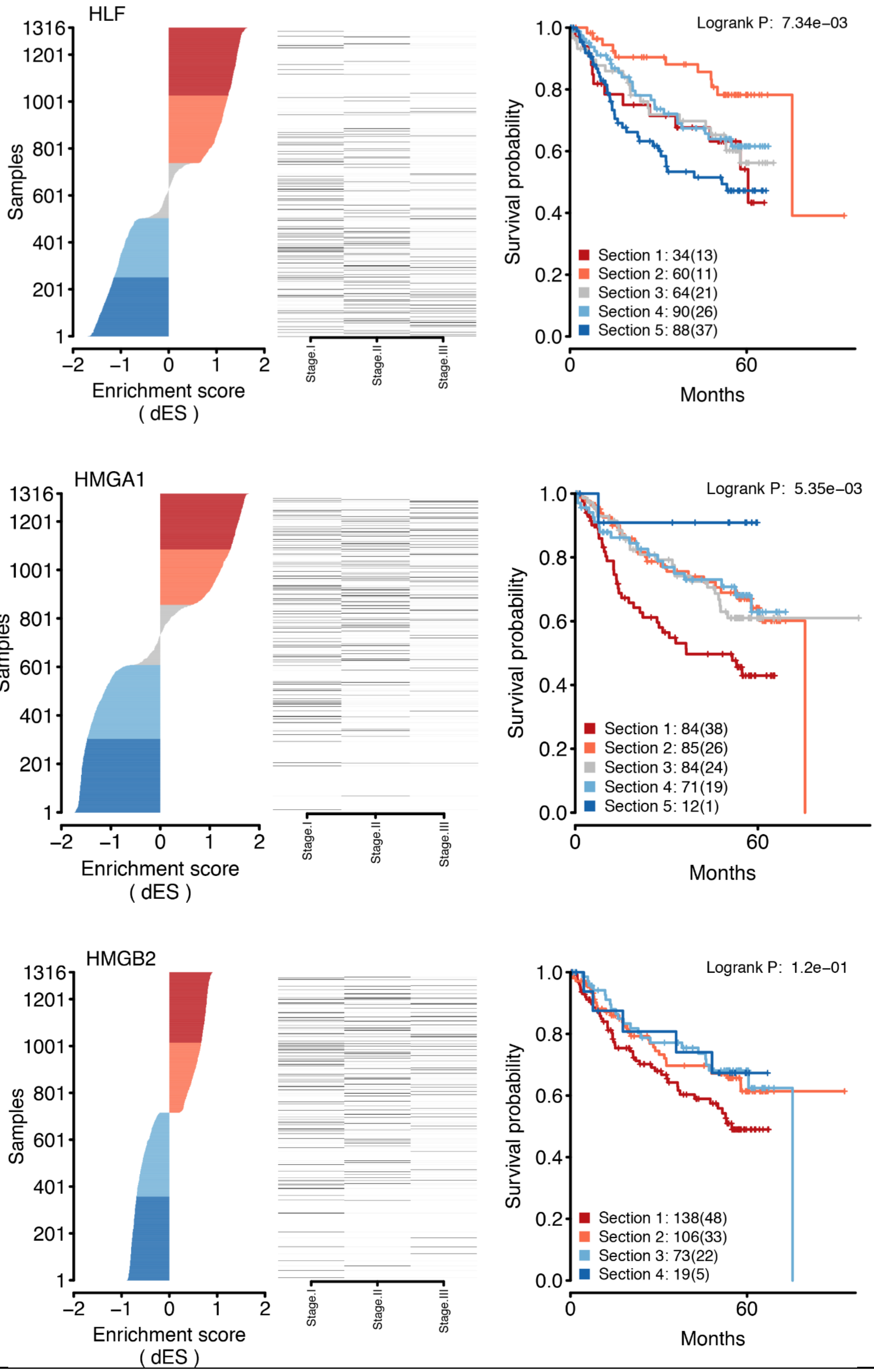
HLF
Logrank P: 7.34e−03
Section 1: 34(13)
Section 2: 60(11)
Section 3: 64(21)
Section 4: 90(26)
Section 5: 88(37)
HMGA1
Logrank P: 5.35e−03
Section 1: 84(38)
Section 2: 85(26)
Section 3: 84(24)
Section 4: 71(19)
Section 5: 12(1)
HMGB2
Logrank P: 1.2e−01
Section 1: 138(48)
Section 2: 106(33)
Section 3: 73(22)
Section 4: 19(5)
Samples
Enrichment score
(dES)
Stage.I
Stage.II
Stage.III
Survival probability
Months

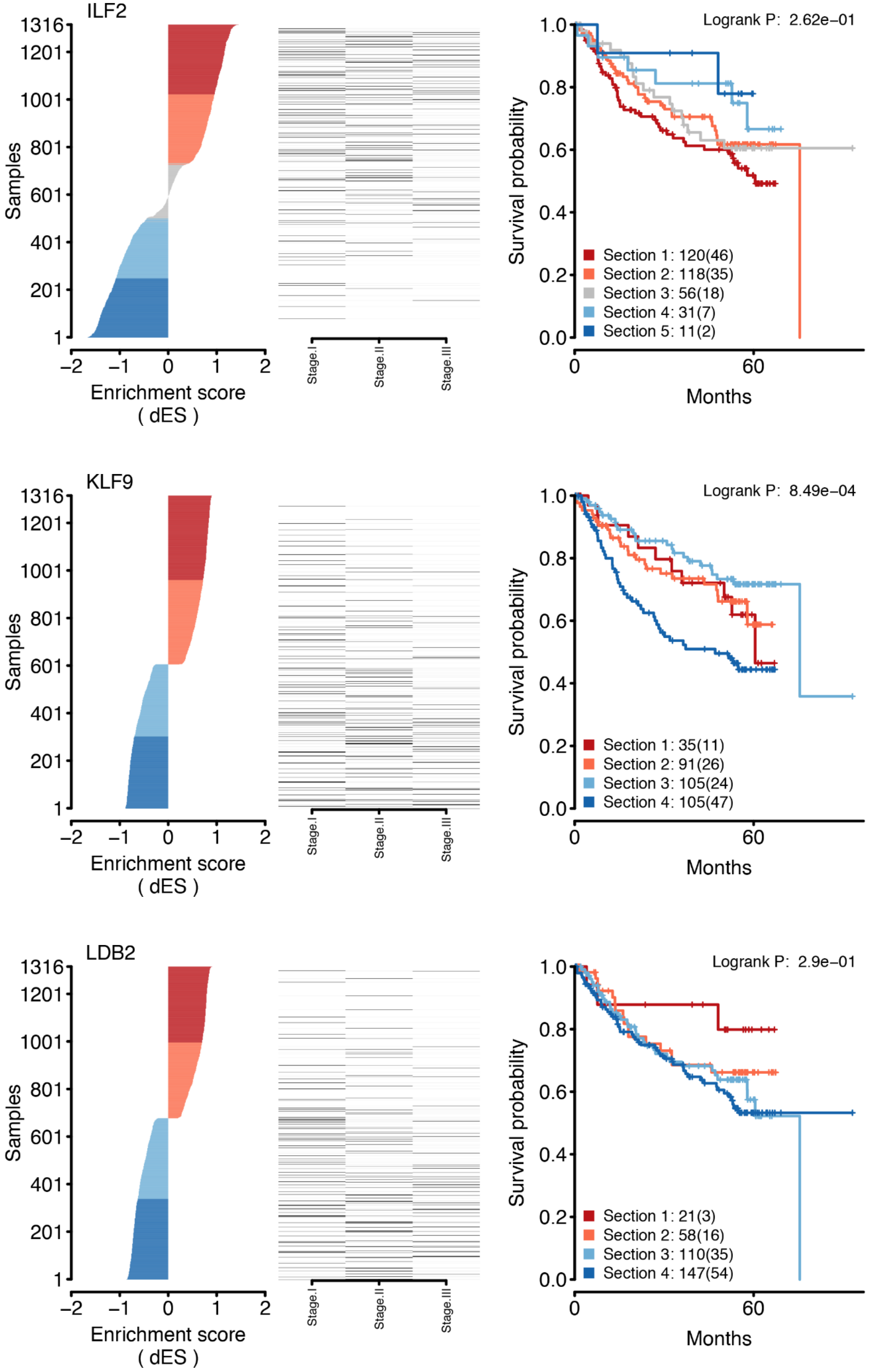
ILF2
Samples
1316
1201
1001
801
601
401
201
1
−2
−1
0
1
2
Enrichment score
(dES)
Stage.I
Stage.II
Stage.III
Logrank P: 2.62e−01
Survival probability
1.0
0.8
0.6
0.4
0.2
0.0
Section 1: 120(46)
Section 2: 118(35)
Section 3: 56(18)
Section 4: 31(7)
Section 5: 11(2)
0
60
Months
KLF9
Logrank P: 8.49e−04
Section 1: 35(11)
Section 2: 91(26)
Section 3: 105(24)
Section 4: 105(47)
LDB2
Logrank P: 2.9e−01
Section 1: 21(3)
Section 2: 58(16)
Section 3: 110(35)
Section 4: 147(54)

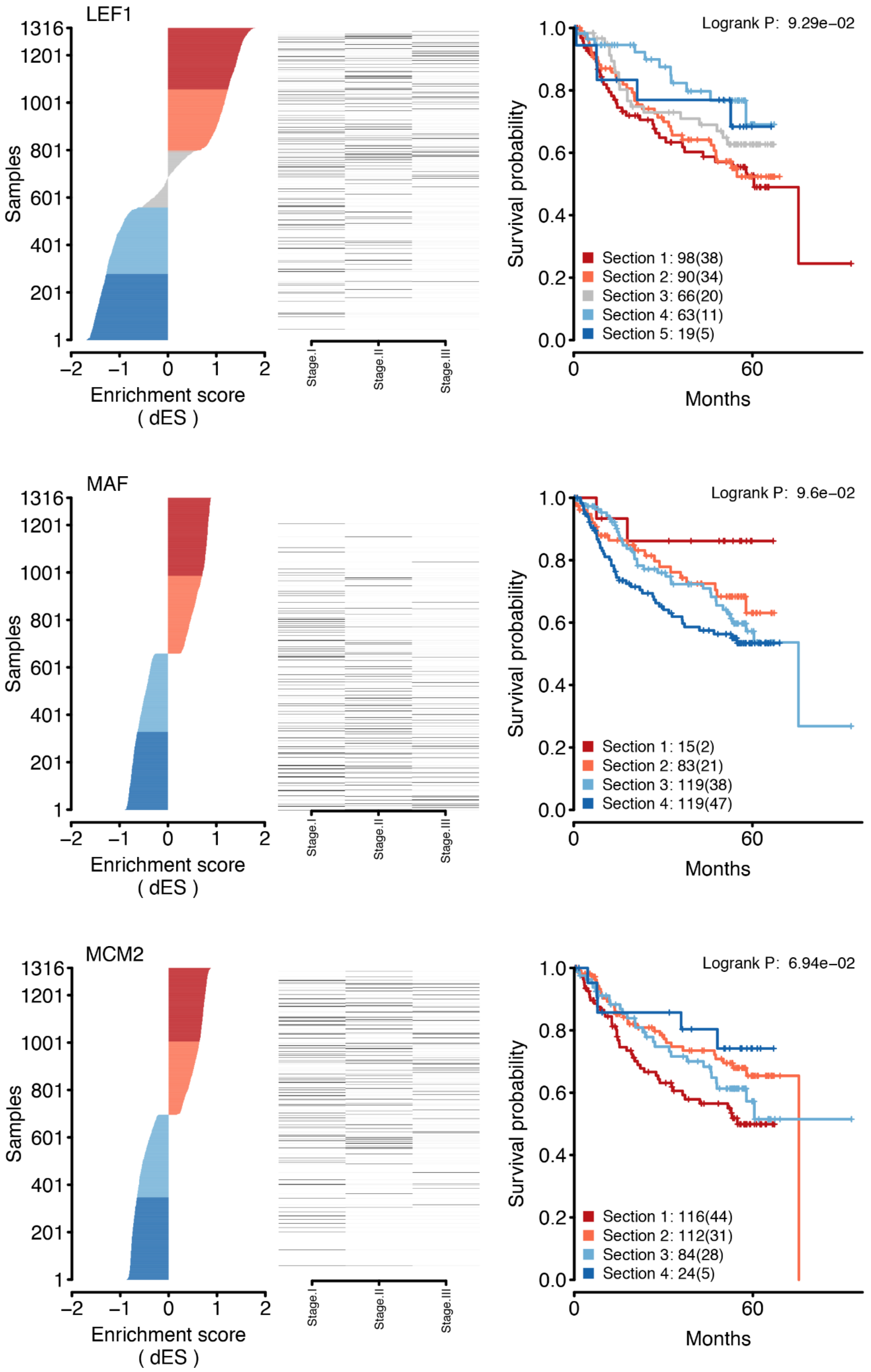
LEF1
Samples
Enrichment score
(dES)
Stage.I
Stage.II
Stage.III
Logrank P: 9.29e−02
Survival probability
Months
Section 1: 98(38)
Section 2: 90(34)
Section 3: 66(20)
Section 4: 63(11)
Section 5: 19(5)
MAF
Samples
Enrichment score
(dES)
Stage.I
Stage.II
Stage.III
Logrank P: 9.6e−02
Survival probability
Months
Section 1: 15(2)
Section 2: 83(21)
Section 3: 119(38)
Section 4: 119(47)
MCM2
Samples
Enrichment score
(dES)
Stage.I
Stage.II
Stage.III
Logrank P: 6.94e−02
Survival probability
Months
Section 1: 116(44)
Section 2: 112(31)
Section 3: 84(28)
Section 4: 24(5)

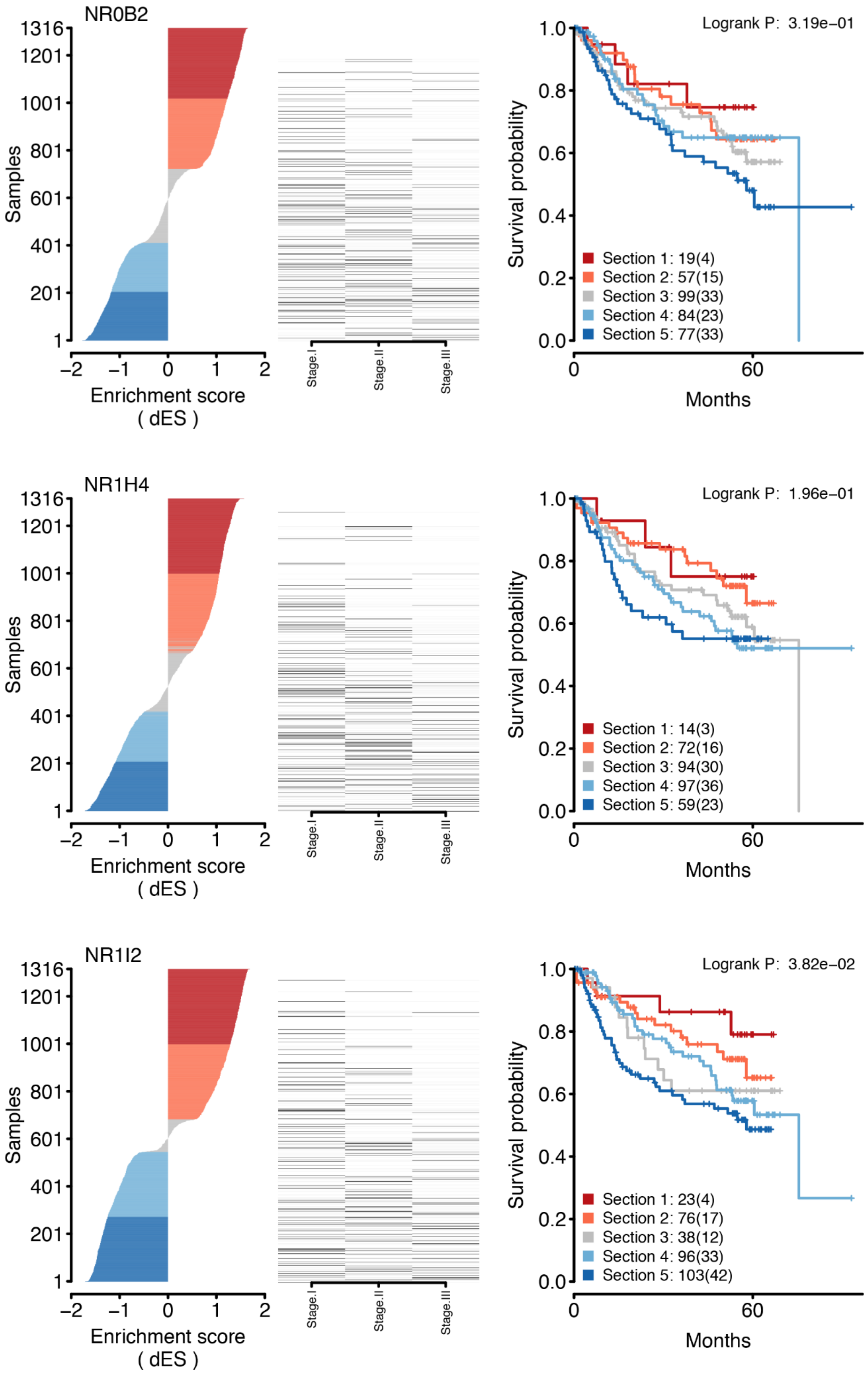
NR0B2
Samples
Enrichment score
(dES)
Stage.I
Stage.II
Stage.III
Logrank P: 3.19e−01
Survival probability
Section 1: 19(4)
Section 2: 57(15)
Section 3: 99(33)
Section 4: 84(23)
Section 5: 77(33)
Months
NR1H4
Logrank P: 1.96e−01
Section 1: 14(3)
Section 2: 72(16)
Section 3: 94(30)
Section 4: 97(36)
Section 5: 59(23)
NR1I2
Logrank P: 3.82e−02
Section 1: 23(4)
Section 2: 76(17)
Section 3: 38(12)
Section 4: 96(33)
Section 5: 103(42)

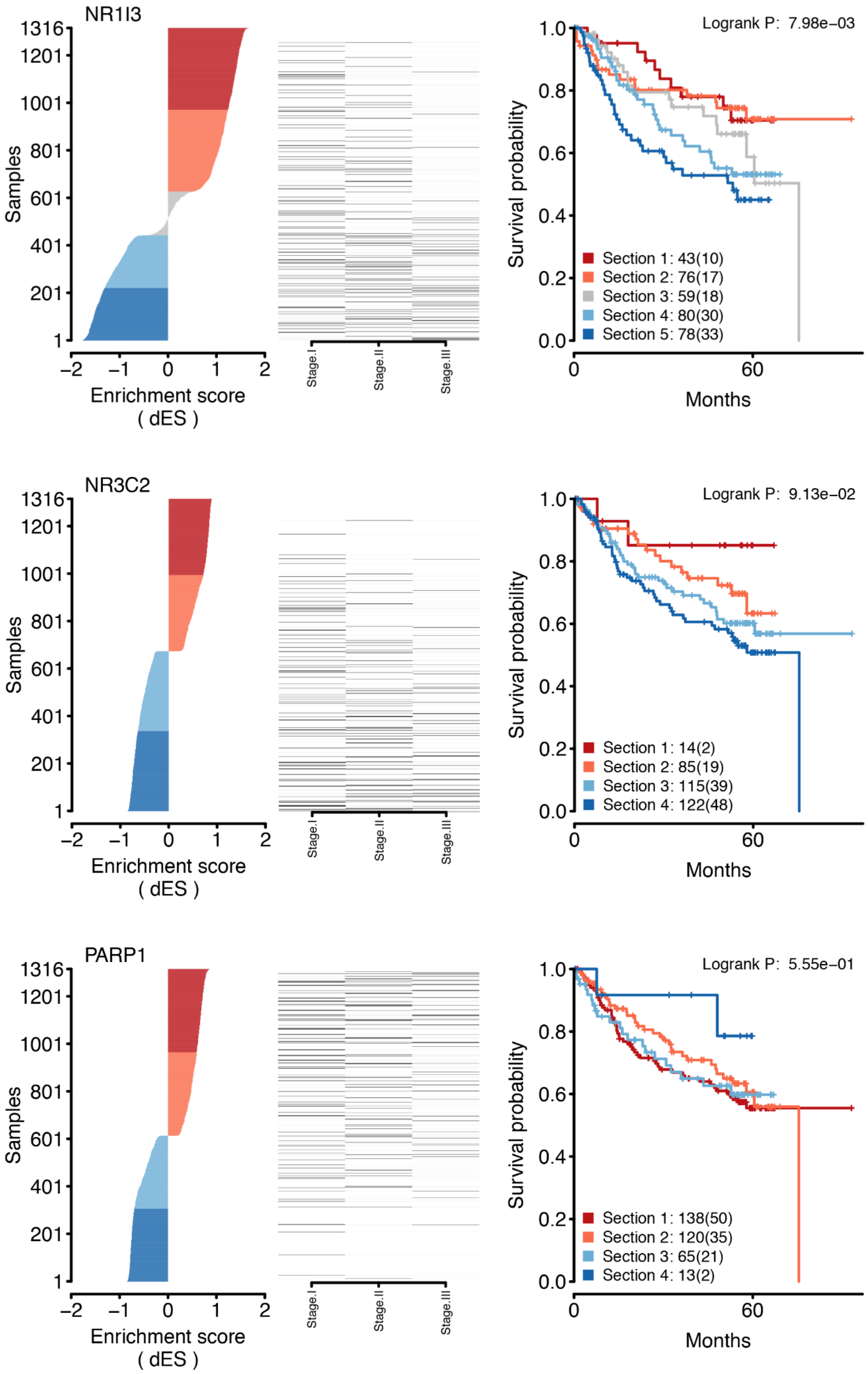
NR1I3
Samples
Enrichment score (dES)
Stage.I
Stage.II
Stage.III
Logrank P: 7.98e−03
Survival probability
Months
Section 1: 43(10)
Section 2: 76(17)
Section 3: 59(18)
Section 4: 80(30)
Section 5: 78(33)
NR3C2
Samples
Enrichment score (dES)
Stage.I
Stage.II
Stage.III
Logrank P: 9.13e−02
Survival probability
Months
Section 1: 14(2)
Section 2: 85(19)
Section 3: 115(39)
Section 4: 122(48)
PARP1
Samples
Enrichment score (dES)
Stage.I
Stage.II
Stage.III
Logrank P: 5.55e−01
Survival probability
Months
Section 1: 138(50)
Section 2: 120(35)
Section 3: 65(21)
Section 4: 13(2)

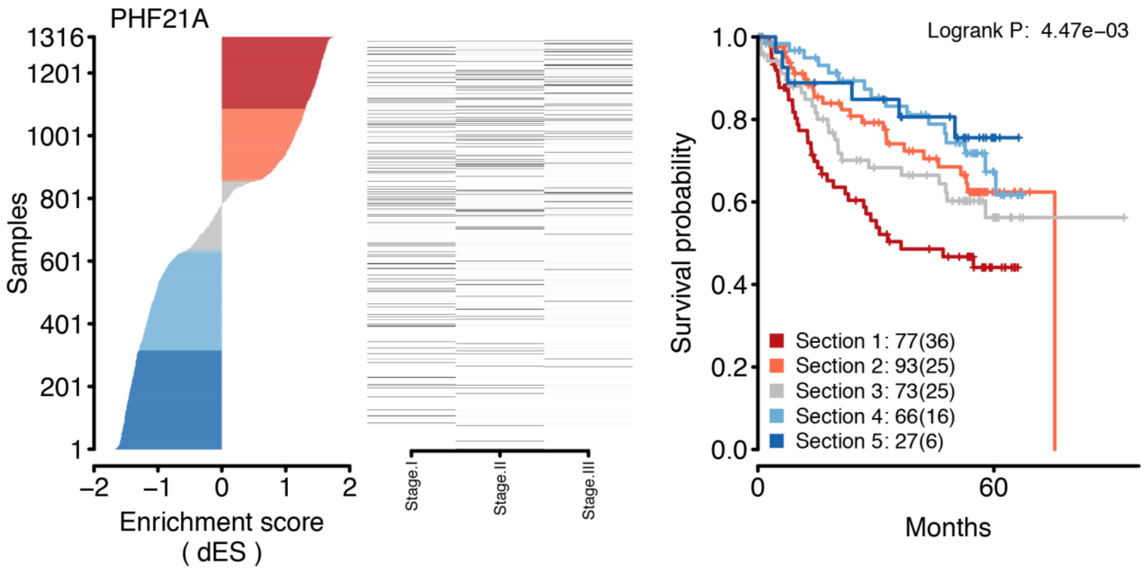
PHF21A
Samples
Enrichment score
(dES)
Stage.I
Stage.II
Stage.III
Logrank P: 4.47e−03
Survival probability
Section 1: 77(36)
Section 2: 93(25)
Section 3: 73(25)
Section 4: 66(16)
Section 5: 27(6)
Months

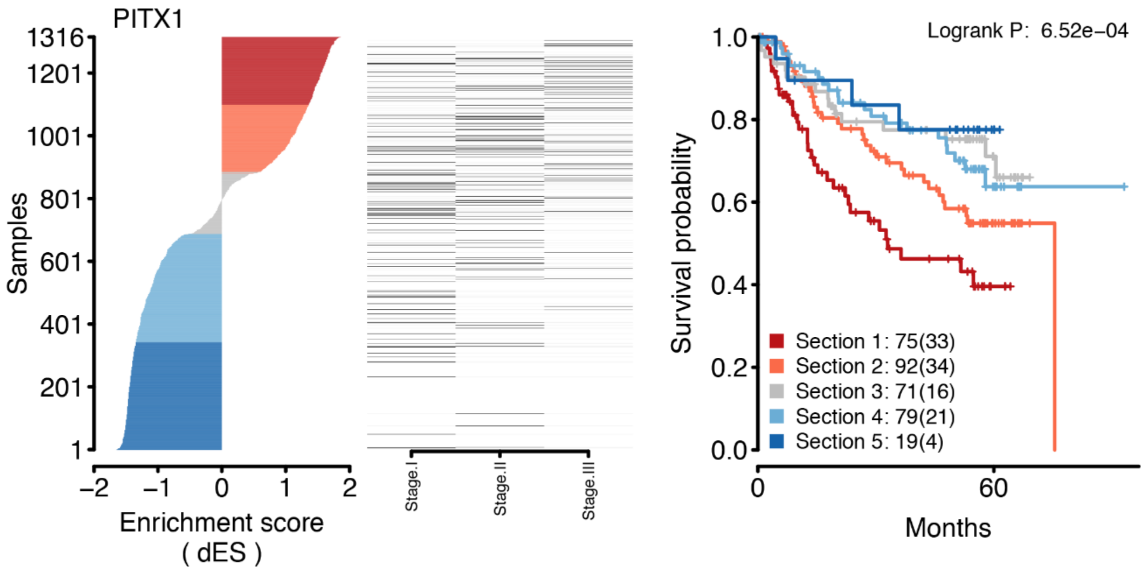
PITX1
Samples
Enrichment score
(dES)
Stage.I
Stage.II
Stage.III
Logrank P: 6.52e−04
Survival probability
Section 1: 75(33)
Section 2: 92(34)
Section 3: 71(16)
Section 4: 79(21)
Section 5: 19(4)
Months

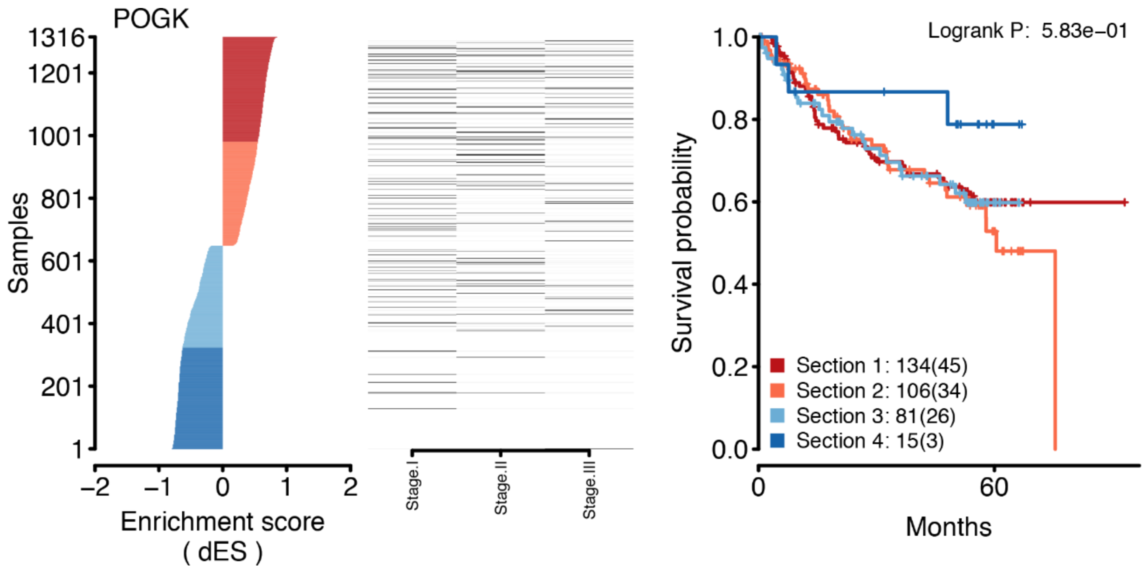
POGK
Samples
Enrichment score
(dES)
Stage.I
Stage.II
Stage.III
Logrank P: 5.83e−01
Survival probability
Section 1: 134(45)
Section 2: 106(34)
Section 3: 81(26)
Section 4: 15(3)
Months

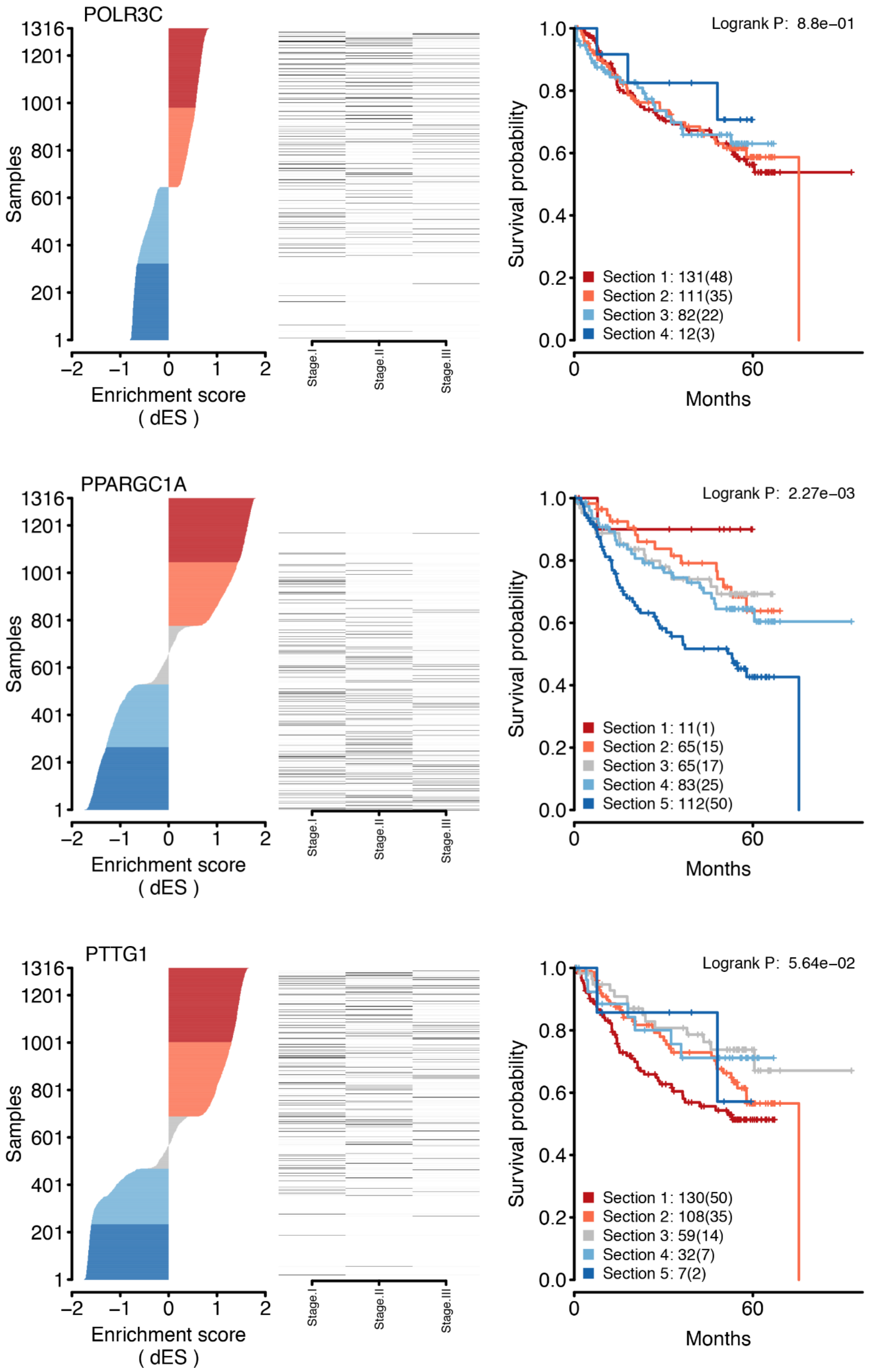
POLR3C
1316
1201
1001
801
601
401
201
1
Samples
−2
−1
0
1
2
Enrichment score
(dES)
Stage.I
Stage.II
Stage.III
Logrank P: 8.8e−01
1.0
0.8
0.6
0.4
0.2
0.0
Survival probability
Section 1: 131(48)
Section 2: 111(35)
Section 3: 82(22)
Section 4: 12(3)
0
60
Months
PPARGC1A
Logrank P: 2.27e−03
Section 1: 11(1)
Section 2: 65(15)
Section 3: 65(17)
Section 4: 83(25)
Section 5: 112(50)
PTTG1
Logrank P: 5.64e−02
Section 1: 130(50)
Section 2: 108(35)
Section 3: 59(14)
Section 4: 32(7)
Section 5: 7(2)

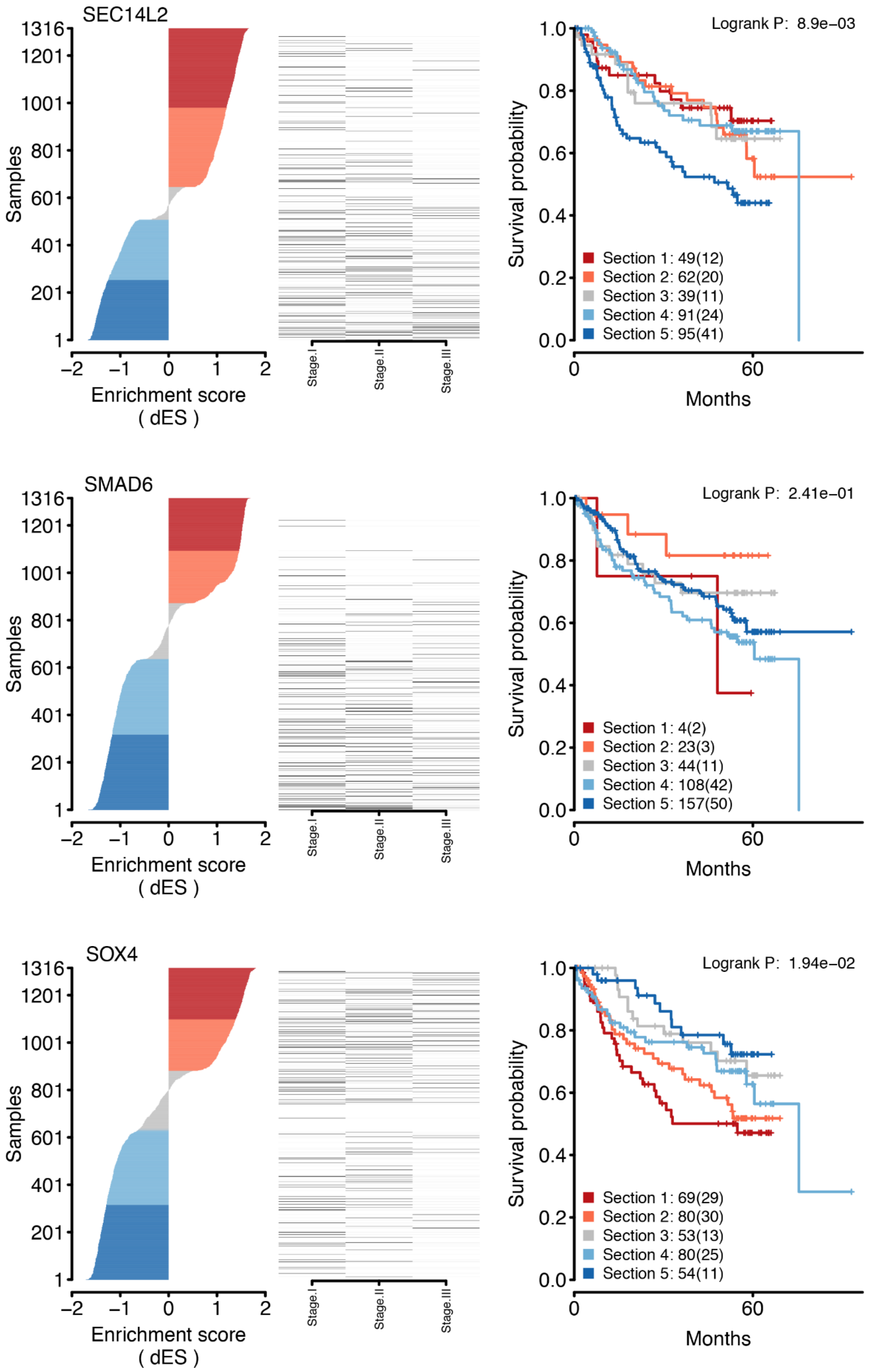

SEC14L2
Samples
1316
1201
1001
801
601
401
201
1
−2
−1
0
1
2
Enrichment score
(dES)
Stage.I
Stage.II
Stage.III
Logrank P: 8.9e−03
Survival probability
1.0
0.8
0.6
0.4
0.2
0.0
0
60
Months
Section 1: 49(12)
Section 2: 62(20)
Section 3: 39(11)
Section 4: 91(24)
Section 5: 95(41)
SMAD6
Logrank P: 2.41e−01
Section 1: 4(2)
Section 2: 23(3)
Section 3: 44(11)
Section 4: 108(42)
Section 5: 157(50)
SOX4
Logrank P: 1.94e−02
Section 1: 69(29)
Section 2: 80(30)
Section 3: 53(13)
Section 4: 80(25)
Section 5: 54(11)

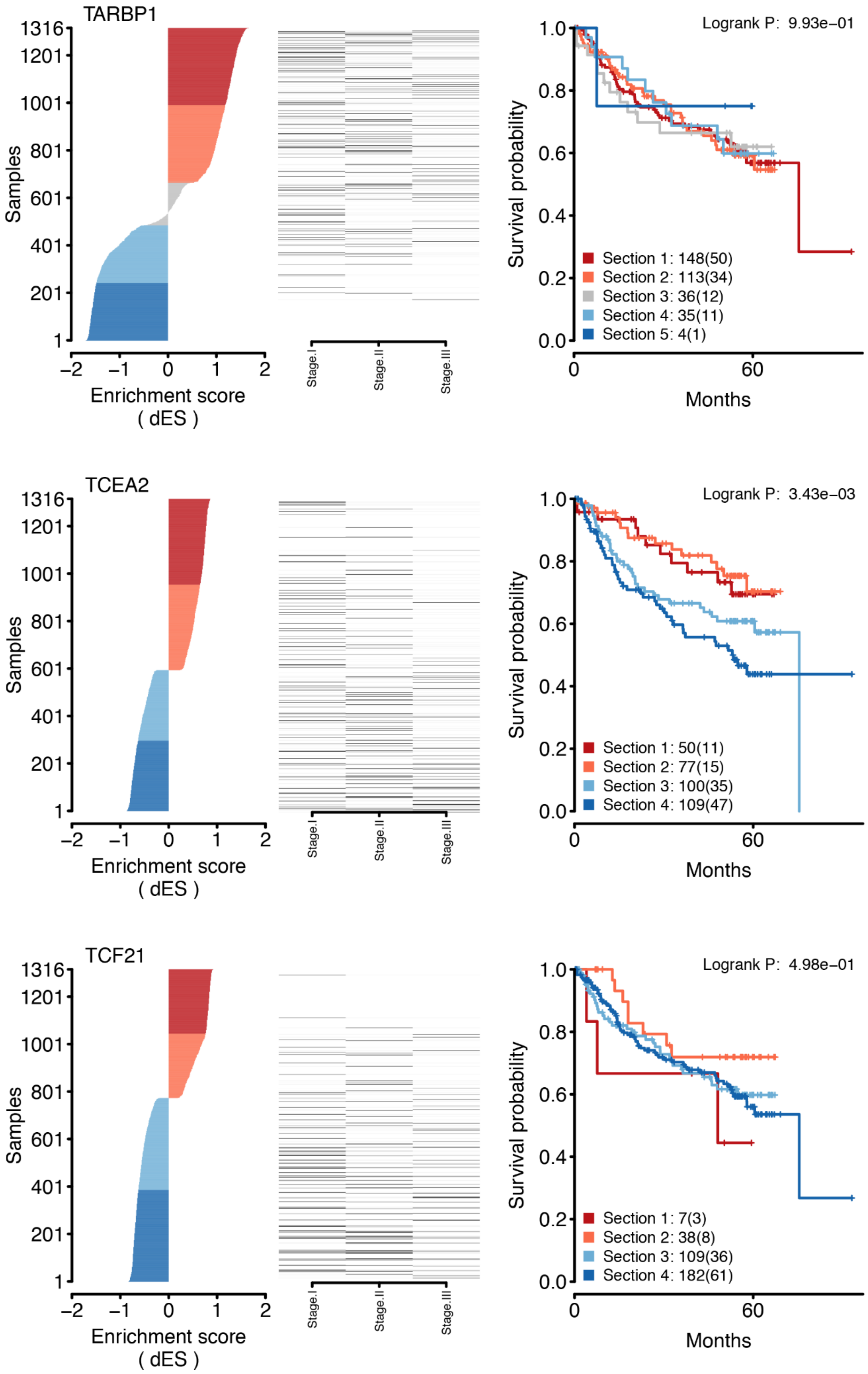
TARBP1
Samples
Enrichment score
(dES)
Stage.I
Stage.II
Stage.III
Survival probability
Months
Logrank P: 9.93e−01
Section 1: 148(50)
Section 2: 113(34)
Section 3: 36(12)
Section 4: 35(11)
Section 5: 4(1)
TCEA2
Samples
Enrichment score
(dES)
Stage.I
Stage.II
Stage.III
Survival probability
Months
Logrank P: 3.43e−03
Section 1: 50(11)
Section 2: 77(15)
Section 3: 100(35)
Section 4: 109(47)
TCF21
Samples
Enrichment score
(dES)
Stage.I
Stage.II
Stage.III
Survival probability
Months
Logrank P: 4.98e−01
Section 1: 7(3)
Section 2: 38(8)
Section 3: 109(36)
Section 4: 182(61)

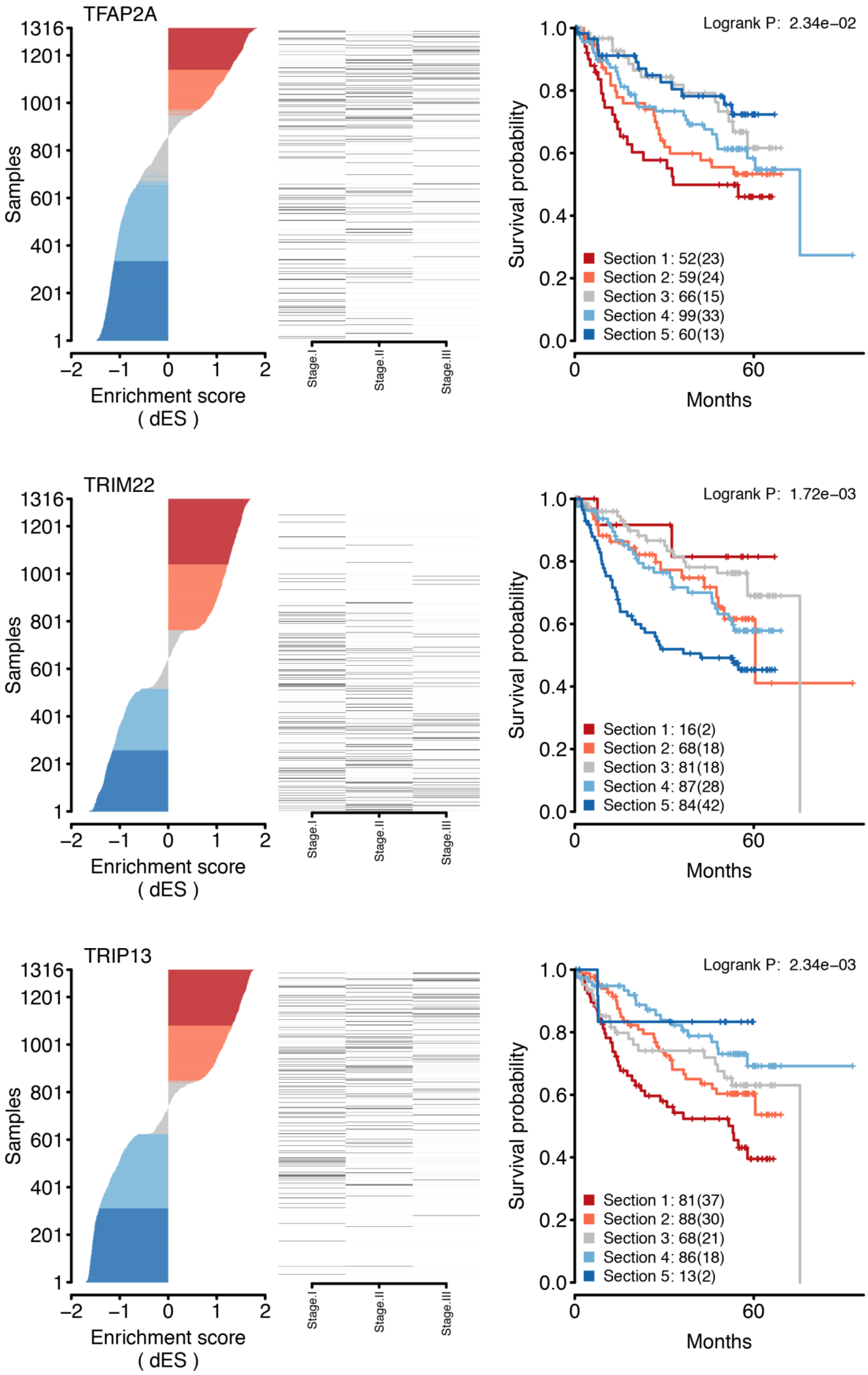
TFAP2A
Samples
Enrichment score (dES)
Stage.I
Stage.II
Stage.III
Logrank P: 2.34e−02
Survival probability
Months
Section 1: 52(23)
Section 2: 59(24)
Section 3: 66(15)
Section 4: 99(33)
Section 5: 60(13)
TRIM22
Samples
Enrichment score (dES)
Stage.I
Stage.II
Stage.III
Logrank P: 1.72e−03
Survival probability
Months
Section 1: 16(2)
Section 2: 68(18)
Section 3: 81(18)
Section 4: 87(28)
Section 5: 84(42)
TRIP13
Samples
Enrichment score (dES)
Stage.I
Stage.II
Stage.III
Logrank P: 2.34e−03
Survival probability
Months
Section 1: 81(37)
Section 2: 88(30)
Section 3: 68(21)
Section 4: 86(18)
Section 5: 13(2)

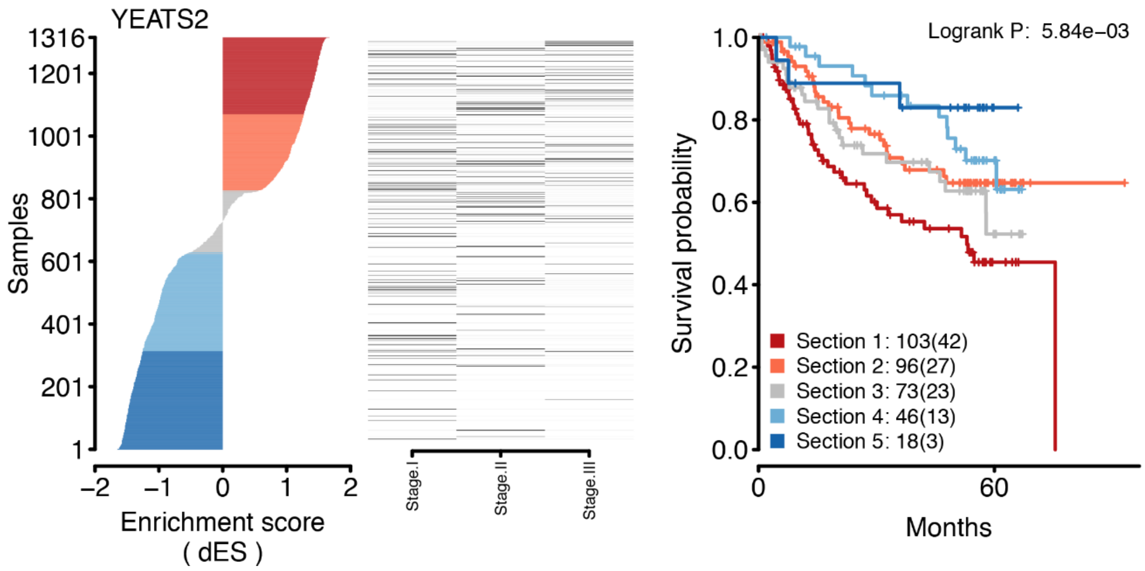

Supplementary Figure 4. Prognosis relevance for identified MRs.

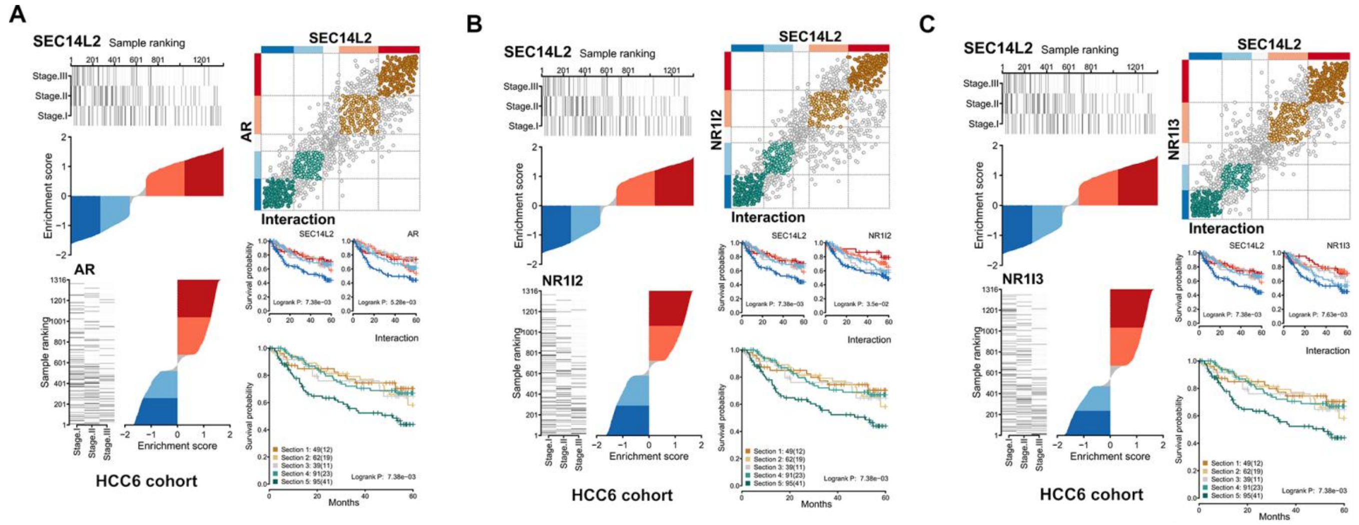

Supplementary Figure 5. Regulon activity of SEC14L2: interaction with multiple NRs. (**A**) SEC14L2-AR interaction. (**B**) SEC14L2-NR1I2 interaction. (**C**) SEC14L2-NR1I3 interaction.

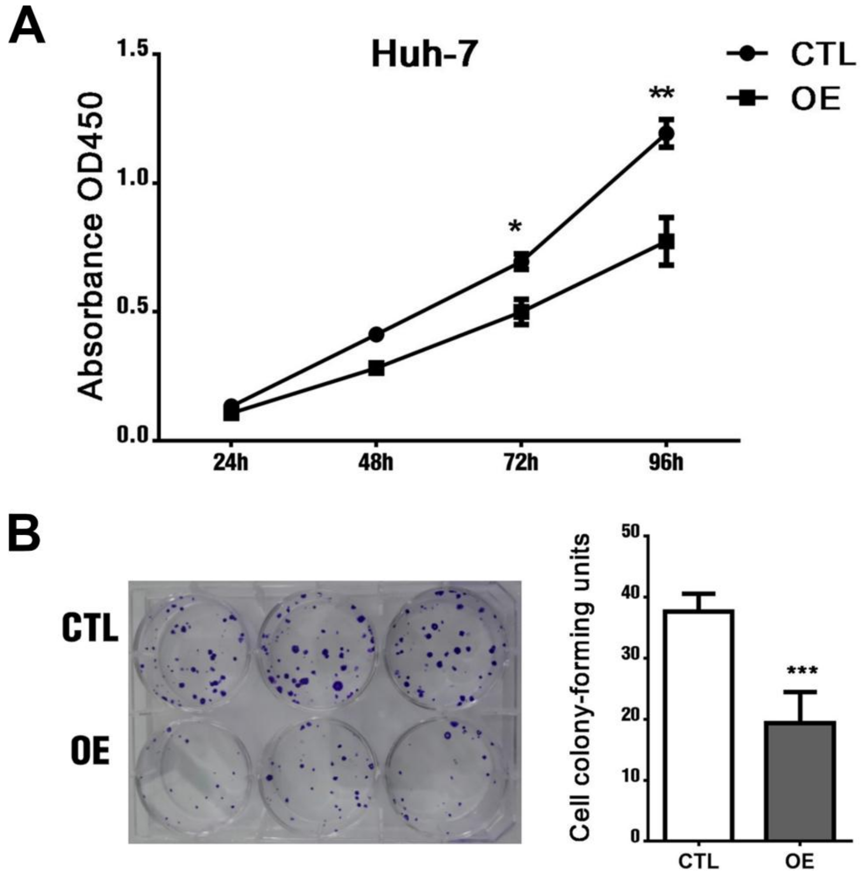

Supplementary Figure 6. SEC14L2 overexpression significantly inhibited cell proliferation in Huh7 cells. (**A**) Proliferation (CCK-8) assay in Huh7 cells. (**B**) Colony formation assay and quantitative analysis.
