# Supplementary material for: Discovering master regulators in hepatocellular carcinoma: one novel MR, SEC14L2 inhibits cancer cells
Source: Aging (Albany NY). 2019 Dec 18;11(24):12375–411. doi: 10.18632/aging.102579 (PMC6949064; doi:10.18632/aging.102579)
Supplement: Supplementary Tables [file aging-11-102579-s007..pdf]

SUPPLEMENTARY TABLES

Supplementary Table 1. HCC datasets used in this study.

ID	Brief introduction	Platform	No. of samples	Note*
**GSE9843**	Gene expression profiling of 91 hepatocellular carcinomas with hepatitis C virus etiology	GPL570	91	
**GSE55092**	Viral Expression and Molecular Profiling in Liver Tissue versus Microdissected Hepatocytes in Hepatitis B Virus - Associated Hepatocellular Carcinoma	GPL570	140	20 profiles from laser capture-microdissected (LCM) hepatocytes
**GSE62232**	Large-scale gene expression profiling of 81 hepatocellular carcinomas	GPL570	81	
**GSE45267**	Gene expression profiles of human hepatocellular carcinoma (training set 1)	GPL570	87	
**GSE45435**	Gene expression profiles of human hepatocellular carcinoma (validation set)	GPL570	31	
**GSE6764**	Genome-wide molecular profiles of HCV-induced dysplasia and hepatocellular carcinoma	GPL570	75	10 normal liver tissues;10 cirrhotic liver tissues;3 cirrhotic liver tissue from patients without HCC;10 low-grade dysplastic liver tissues;7 high-grade dysplastic liver tissues
**GSE17548**	Expression data from cirrhosis and HCC tissue samples	GPL570	30	
**GSE6222**	Genome-wide analysis of gene expression patterns in human liver cancers	GPL570	13	3 cell lines
**GSE40873**	Low SLC22A7 expression in noncancerous liver promotes hepatocellular carcinoma occurrence - a prospective study	GPL570	49	49 noncancerous liver tissues of HCC patients within Milan criteria
**GSE15765**	Identification of cholangiocarcinoma-like gene expression traits in hepatocellular carcinoma	GPL571	90	13 cholangiocarcinoma; 7 combined hepatocellular carcinoma and cholangiocarcinoma
**GSE17967**	RMA expression data for liver samples from subjects with HCV cirrhosis with and without concomitant HCC	GPL571	63	47 cirrhosis
**GSE14520**	Gene expression data of human hepatocellular carcinoma (HCC)	GPL571;GPL3921	488	
**GSE14323**	RMA expression data for liver samples from subjects with HCV, HCV-HCC, or normal liver	GPL96;GPL571	124	GPL571: 19 Normal;41 cirrhosis; 17 cirrhosisHCC; 12 HCC
**GSE35306**	Combined hepatocellular-cholangiocarcinomas exhibit progenitor features and activation of Wnt and TGFbeta signaling pathways	GPL6244	30	3 intrahepatic cholangiocarcinoma;20 combined hepatocellular-cholangiocarcinoma;7 HCC
**GSE64041**	Gene expression profiling in paired human hepatocellular carcinoma and liver parenchyma biopsies and normal liver biopsies.	GPL6244	65	60HCC; 5 healthy liver biopsies

**GSE36376**	Gene Expression Profiles of both tumor and adjacent non-tumor liver Identify Hepatocellular Carcinoma Patients at High Risk of Recurrence after Curative Hepatectomy	GPL10558	433
**GSE76427**	Microarray expression data for tumor and adjacent non-tumor tissues from hepatocellular carcinoma patients	GPL10558	167
**GSE39791**	Hepatic regeneration gene expression signature predicts late recurrence of hepatocellular carcinoma	GPL10558	144
**GSE43619**	Activation of beta-catenin in hepatocellular carcinoma (HCC patients)	GPL10558	88
**GSE57957**	Expression profile of Hepatocellular Carcinoma	GPL10558	78
**GSE60502**	Gene expression profiling of 18 hepatocellular carcinoma and adjacent non-tumorous liver tissue	GPL96	36

*Gene expression profiles from cell lines, hepatocytes, and cholangiocarcinoma were excluded.

Please browse Full Text version to see the data of Supplementary Tables 1 and 2.

Supplementary Table 2. Differentially expressed genes in HCC6.

Supplementary Table 3. MRA analysis on the filtered transcriptional network constructed for HCC6.

Supplementary Table 4. HCC subtype-related MRs.

Supplementary Table 5. HCC etiology-related MRs.

Supplementary Table 6. Regulons: MRs and their targets.

Supplementary Table 7. MR-based survival test.

Supplementary Table 8. Summary of previous knowledge of MRs.
