# Supplementary material for: Discovering master regulators in hepatocellular carcinoma: one novel MR, SEC14L2 inhibits cancer cells
Source: Aging (Albany NY). 2019 Dec 18;11(24):12375–411. doi: 10.18632/aging.102579 (PMC6949064; doi:10.18632/aging.102579)
Supplement: Supplementary Table 3 [file aging-11-102579-s003..docx]

**Table S3: MRA analysis^1^ on the filtered transcriptional network calculated for HCC6**

| MRA rank^2^ | Regulon | Universe Size^3^ | Regulon Size^4^ | Total Hits^5^ | Expected Hits^6^ | Observed Hits^7^ | *P*-value | Adjusted *P*-value |
| --- | --- | --- | --- | --- | --- | --- | --- | --- |
| 1 | ESR1 | 12100 | 180 | 483 | 7.19 | 97 | 3.40E-89 | 3.7E-86 |
| 2 | PTTG1 | 12100 | 169 | 483 | 6.75 | 87 | 2.00E-77 | 1.1E-74 |
| 3 | NR1I2 | 12100 | 217 | 483 | 8.66 | 93 | 1.90E-73 | 6.7E-71 |
| 4 | KLF11 | 12100 | 90 | 483 | 3.59 | 64 | 5.20E-70 | 1.4E-67 |
| 5 | HMGA1 | 12100 | 69 | 483 | 2.75 | 45 | 1.40E-46 | 3.1E-44 |
| 6 | PPARGC1A | 12100 | 108 | 483 | 4.31 | 53 | 1.40E-45 | 2.6E-43 |
| 7 | NR0B2 | 12100 | 158 | 483 | 6.31 | 58 | 5.80E-41 | 8.9E-39 |
| 8 | PRDM4 | 12100 | 104 | 483 | 4.15 | 48 | 1.10E-39 | 1.5E-37 |
| 9 | NFE2L3 | 12100 | 118 | 483 | 4.71 | 50 | 4.90E-39 | 5.9E-37 |
| 10 | NR1I3 | 12100 | 161 | 483 | 6.43 | 56 | 4.50E-38 | 4.8E-36 |
| 11 | NR1H4 | 12100 | 233 | 483 | 9.3 | 61 | 2.10E-33 | 2.1E-31 |
| 12 | PITX1 | 12100 | 87 | 483 | 3.47 | 40 | 4.00E-33 | 3.6E-31 |
| 13 | AR | 12100 | 121 | 483 | 4.83 | 45 | 3.40E-32 | 2.8E-30 |
| 14 | PHF21A | 12100 | 83 | 483 | 3.31 | 38 | 2.00E-31 | 1.5E-29 |
| 15 | FOS | 12100 | 63 | 483 | 2.51 | 34 | 2.30E-31 | 1.6E-29 |
| 16 | ETS2 | 12100 | 101 | 483 | 4.03 | 41 | 3.10E-31 | 2.1E-29 |
| 17 | POLR1E | 12100 | 51 | 483 | 2.04 | 29 | 8.20E-28 | 5.2E-26 |
| 18 | NR3C2 | 12100 | 77 | 483 | 3.07 | 34 | 1.50E-27 | 8.7E-26 |
| 19 | TRIP13 | 12100 | 112 | 483 | 4.47 | 38 | 1.30E-25 | 6.8E-24 |
| 20 | HEY1 | 12100 | 29 | 483 | 1.16 | 22 | 1.30E-25 | 6.8E-24 |
| 21 | DACH1 | 12100 | 71 | 483 | 2.83 | 31 | 4.90E-25 | 2.5E-23 |
| 22 | GTF3C2 | 12100 | 46 | 483 | 1.84 | 26 | 5.90E-25 | 2.9E-23 |
| 23 | SMARCA4 | 12100 | 85 | 483 | 3.39 | 33 | 1.40E-24 | 6.5E-23 |
| 24 | LHX2 | 12100 | 32 | 483 | 1.28 | 22 | 4.70E-24 | 2.1E-22 |
| 25 | TFAP2A | 12100 | 118 | 483 | 4.71 | 36 | 1.60E-22 | 6.7E-21 |
| 26 | E2F8 | 12100 | 32 | 483 | 1.28 | 21 | 2.40E-22 | 9.7E-21 |
| 27 | ETV4 | 12100 | 45 | 483 | 1.8 | 24 | 2.60E-22 | 1.1E-20 |
| 28 | TCF21 | 12100 | 79 | 483 | 3.15 | 29 | 6.20E-21 | 2.4E-19 |
| 29 | TARBP1 | 12100 | 140 | 483 | 5.59 | 35 | 8.20E-19 | 3.0E-17 |
| 30 | LEF1 | 12100 | 79 | 483 | 3.15 | 27 | 1.20E-18 | 4.4E-17 |
| 31 | HLF | 12100 | 120 | 483 | 4.79 | 32 | 3.30E-18 | 1.2E-16 |
| 32 | BCL11A | 12100 | 69 | 483 | 2.75 | 25 | 4.70E-18 | 1.6E-16 |
| 33 | BARD1 | 12100 | 52 | 483 | 2.08 | 22 | 9.30E-18 | 3.0E-16 |
| 34 | SEC14L2 | 12100 | 199 | 483 | 7.94 | 40 | 1.10E-17 | 3.3E-16 |
| 35 | SMAD6 | 12100 | 20 | 483 | 0.8 | 15 | 1.10E-17 | 3.3E-16 |
| 36 | ASCL1 | 12100 | 79 | 483 | 3.15 | 26 | 1.60E-17 | 4.8E-16 |
| 37 | TEAD4 | 12100 | 74 | 483 | 2.95 | 24 | 4.20E-16 | 1.2E-14 |
| 38 | FOXM1 | 12100 | 70 | 483 | 2.79 | 23 | 1.30E-15 | 3.5E-14 |
| 39 | YEATS2 | 12100 | 119 | 483 | 4.75 | 29 | 1.80E-15 | 4.9E-14 |
| 40 | CBX6 | 12100 | 52 | 483 | 2.08 | 20 | 2.80E-15 | 7.4E-14 |
| 41 | SATB2 | 12100 | 27 | 483 | 1.08 | 15 | 9.40E-15 | 2.5E-13 |
| 42 | DIP2C | 12100 | 36 | 483 | 1.44 | 16 | 1.10E-13 | 2.9E-12 |
| 43 | NCOR1 | 12100 | 55 | 483 | 2.2 | 19 | 1.40E-13 | 3.4E-12 |
| 44 | TTF2 | 12100 | 38 | 483 | 1.52 | 16 | 3.20E-13 | 7.8E-12 |
| 45 | MAFG | 12100 | 86 | 483 | 3.43 | 22 | 1.70E-12 | 3.9E-11 |
| 46 | E2F3 | 12100 | 57 | 483 | 2.28 | 18 | 3.60E-12 | 8.3E-11 |
| 47 | MYEF2 | 12100 | 51 | 483 | 2.04 | 17 | 5.30E-12 | 1.2E-10 |
| 48 | ZSCAN2 | 12100 | 38 | 483 | 1.52 | 15 | 5.60E-12 | 1.2E-10 |
| 49 | RBBP7 | 12100 | 67 | 483 | 2.67 | 19 | 7.60E-12 | 1.7E-10 |
| 50 | MAF | 12100 | 53 | 483 | 2.12 | 17 | 1.10E-11 | 2.3E-10 |
| 51 | HDAC2 | 12100 | 53 | 483 | 2.12 | 17 | 1.10E-11 | 2.3E-10 |
| 52 | TCEA2 | 12100 | 72 | 483 | 2.87 | 19 | 3.10E-11 | 6.3E-10 |
| 53 | LDB2 | 12100 | 64 | 483 | 2.55 | 18 | 3.20E-11 | 6.4E-10 |
| 54 | PAX6 | 12100 | 45 | 483 | 1.8 | 15 | 9.60E-11 | 1.9E-09 |
| 55 | KLF9 | 12100 | 46 | 483 | 1.84 | 15 | 1.40E-10 | 2.7E-09 |
| 56 | ID1 | 12100 | 18 | 483 | 0.72 | 10 | 3.10E-10 | 5.9E-09 |
| 57 | PER3 | 12100 | 24 | 483 | 0.96 | 11 | 5.70E-10 | 1.1E-08 |
| 58 | HOXA5 | 12100 | 19 | 483 | 0.76 | 10 | 6.30E-10 | 1.1E-08 |
| 59 | FOSB | 12100 | 30 | 483 | 1.2 | 12 | 6.40E-10 | 1.1E-08 |
| 60 | HEXIM1 | 12100 | 30 | 483 | 1.2 | 12 | 6.40E-10 | 1.1E-08 |
| 61 | SMARCD1 | 12100 | 116 | 483 | 4.63 | 22 | 8.90E-10 | 1.6E-08 |
| 62 | HDAC1 | 12100 | 36 | 483 | 1.44 | 12 | 7.50E-09 | 1.3E-07 |
| 63 | HOXB7 | 12100 | 47 | 483 | 1.88 | 13 | 2.30E-08 | 3.8E-07 |
| 64 | TBC1D2B | 12100 | 47 | 483 | 1.88 | 13 | 2.30E-08 | 3.8E-07 |
| 65 | IRF8 | 12100 | 26 | 483 | 1.04 | 10 | 2.80E-08 | 4.6E-07 |
| 66 | MCM2 | 12100 | 49 | 483 | 1.96 | 13 | 3.90E-08 | 6.4E-07 |
| 67 | NOTCH3 | 12100 | 130 | 483 | 5.19 | 21 | 4.20E-08 | 6.7E-07 |
| 68 | TOB1 | 12100 | 35 | 483 | 1.4 | 11 | 6.40E-08 | 1.0E-06 |
| 69 | SSX1 | 12100 | 60 | 483 | 2.4 | 14 | 6.90E-08 | 1.1E-06 |
| 70 | EGR1 | 12100 | 22 | 483 | 0.88 | 9 | 7.50E-08 | 1.1E-06 |
| 71 | SMAD3 | 12100 | 52 | 483 | 2.08 | 13 | 8.50E-08 | 1.3E-06 |
| 72 | TAF1B | 12100 | 29 | 483 | 1.16 | 10 | 9.50E-08 | 1.4E-06 |
| 73 | ZNF124 | 12100 | 17 | 483 | 0.68 | 8 | 1.10E-07 | 1.6E-06 |
| 74 | PARP1 | 12100 | 45 | 483 | 1.8 | 12 | 1.20E-07 | 1.8E-06 |
| 75 | HSF2 | 12100 | 25 | 483 | 1 | 9 | 2.80E-07 | 3.9E-06 |
| 76 | ZNF292 | 12100 | 26 | 483 | 1.04 | 9 | 4.10E-07 | 5.7E-06 |
| 77 | ARID3A | 12100 | 150 | 483 | 5.99 | 21 | 5.10E-07 | 7.1E-06 |
| 78 | GTF2IRD1 | 12100 | 28 | 483 | 1.12 | 9 | 8.40E-07 | 1.1E-05 |
| 79 | RORA | 12100 | 21 | 483 | 0.84 | 8 | 7.80E-07 | 1.1E-05 |
| 80 | EZH2 | 12100 | 84 | 483 | 3.35 | 15 | 9.80E-07 | 1.3E-05 |
| 81 | ZNF623 | 12100 | 64 | 483 | 2.55 | 13 | 1.10E-06 | 1.5E-05 |
| 82 | TRIM31 | 12100 | 23 | 483 | 0.92 | 8 | 1.80E-06 | 2.3E-05 |
| 83 | TULP3 | 12100 | 51 | 483 | 2.04 | 11 | 4.10E-06 | 5.3E-05 |
| 84 | POLR3C | 12100 | 45 | 483 | 1.8 | 10 | 8.50E-06 | 1.1E-04 |
| 85 | SOX4 | 12100 | 83 | 483 | 3.31 | 13 | 2.30E-05 | 2.9E-04 |
| 86 | RFX5 | 12100 | 51 | 483 | 2.04 | 10 | 2.70E-05 | 3.4E-04 |
| 87 | MCM6 | 12100 | 17 | 483 | 0.68 | 6 | 3.30E-05 | 4.1E-04 |
| 88 | ZNF544 | 12100 | 36 | 483 | 1.44 | 8 | 6.90E-05 | 8.4E-04 |
| 89 | HDAC11 | 12100 | 38 | 483 | 1.52 | 8 | 1.00E-04 | 1.2E-03 |
| 90 | MYB | 12100 | 39 | 483 | 1.56 | 8 | 1.30E-04 | 1.5E-03 |
| 91 | DDIT3 | 12100 | 52 | 483 | 2.08 | 9 | 1.90E-04 | 2.2E-03 |
| 92 | CEBPD | 12100 | 23 | 483 | 0.92 | 6 | 2.20E-04 | 2.6E-03 |
| 93 | ILF2 | 12100 | 274 | 483 | 10.94 | 24 | 2.60E-04 | 3.0E-03 |
| 94 | ZFP36 | 12100 | 33 | 483 | 1.32 | 7 | 2.70E-04 | 3.1E-03 |
| 95 | ANKS1A | 12100 | 24 | 483 | 0.96 | 6 | 2.90E-04 | 3.2E-03 |
| 96 | PER2 | 12100 | 26 | 483 | 1.04 | 6 | 4.60E-04 | 5.1E-03 |
| 97 | PCGF2 | 12100 | 47 | 483 | 1.88 | 8 | 4.80E-04 | 5.4E-03 |
| 98 | MSC | 12100 | 72 | 483 | 2.87 | 10 | 5.40E-04 | 5.9E-03 |
| 99 | SMAD7 | 12100 | 18 | 483 | 0.72 | 5 | 5.50E-04 | 6.0E-03 |
| 100 | GTF2E1 | 12100 | 27 | 483 | 1.08 | 6 | 5.70E-04 | 6.1E-03 |
| 101 | HMGN4 | 12100 | 87 | 483 | 3.47 | 11 | 6.60E-04 | 7.0E-03 |
| 102 | HMGB2 | 12100 | 28 | 483 | 1.12 | 6 | 7.00E-04 | 7.3E-03 |
| 103 | HDGF | 12100 | 30 | 483 | 1.2 | 6 | 1.00E-03 | 1.1E-02 |
| 104 | SIX1 | 12100 | 30 | 483 | 1.2 | 6 | 1.00E-03 | 1.1E-02 |
| 105 | MCM3 | 12100 | 31 | 483 | 1.24 | 6 | 1.20E-03 | 1.2E-02 |
| 106 | ELF4 | 12100 | 253 | 483 | 10.1 | 21 | 1.20E-03 | 1.2E-02 |
| 107 | TRIM22 | 12100 | 94 | 483 | 3.75 | 11 | 1.30E-03 | 1.3E-02 |
| 108 | EHMT2 | 12100 | 43 | 483 | 1.72 | 7 | 1.40E-03 | 1.4E-02 |
| 109 | ENO1 | 12100 | 125 | 483 | 4.99 | 13 | 1.40E-03 | 1.4E-02 |
| 110 | SMARCA2 | 12100 | 44 | 483 | 1.76 | 7 | 1.60E-03 | 1.6E-02 |
| 111 | CHD7 | 12100 | 44 | 483 | 1.76 | 7 | 1.60E-03 | 1.6E-02 |
| 112 | ZNF165 | 12100 | 47 | 483 | 1.88 | 7 | 2.40E-03 | 2.3E-02 |
| 113 | TRIM32 | 12100 | 47 | 483 | 1.88 | 7 | 2.40E-03 | 2.3E-02 |
| 114 | TRIB3 | 12100 | 75 | 483 | 2.99 | 9 | 2.90E-03 | 2.7E-02 |
| 115 | ERCC8 | 12100 | 50 | 483 | 2 | 7 | 3.50E-03 | 3.2E-02 |
| 116 | SMARCC1 | 12100 | 50 | 483 | 2 | 7 | 3.50E-03 | 3.2E-02 |
| 117 | POGK | 12100 | 38 | 483 | 1.52 | 6 | 3.70E-03 | 3.3E-02 |
| 118 | ZNF189 | 12100 | 17 | 483 | 0.68 | 4 | 3.90E-03 | 3.6E-02 |
| 119 | ARNT2 | 12100 | 18 | 483 | 0.72 | 4 | 4.90E-03 | 4.4E-02 |
| 120 | ZBTB20 | 12100 | 41 | 483 | 1.64 | 6 | 5.40E-03 | 4.8E-02 |

1Significant regulons for *P* < 0.001 (hypergeometric test) are shown. MRs from the extended MR list are highlighted in green.
2In addition, the rank and adjusted p-value of all five MRs (relevant in all three experiments) are shown highlighted in red.

3Number of genes in the transcriptional network.
4Number of genes in a given regulon.
5Number of genes in the meta-PCNA signature.
6Expected overlap between "*total hits*" and "*regulon size*".
7Observed overlap between "*total hits*" and "*regulon size*"

**MRA analysis on the filtered transcriptional network from GSE14520**

| MRA rank | Regulon | Universe.Size | Regulon.Size | Total.Hits | Expected.Hits | Observed.Hits | Pvalue | Adjusted.Pvalue |
| --- | --- | --- | --- | --- | --- | --- | --- | --- |
| 1 | SEC14L2 | 12127 | 289 | 2683 | 63.94 | 207 | 8.20E-74 | 9.20E-71 |
| 2 | SNRPD1 | 12127 | 162 | 2683 | 35.84 | 124 | 1.10E-49 | 6.30E-47 |
| 3 | NR1I3 | 12127 | 150 | 2683 | 33.19 | 117 | 1.90E-48 | 7.20E-46 |
| 4 | ILF2 | 12127 | 133 | 2683 | 29.43 | 106 | 1.30E-45 | 3.70E-43 |
| 5 | PTTG1 | 12127 | 82 | 2683 | 18.14 | 75 | 2.30E-41 | 5.20E-39 |
| 6 | NR1H4 | 12127 | 149 | 2683 | 32.97 | 105 | 1.20E-36 | 2.20E-34 |
| 7 | NR1I2 | 12127 | 182 | 2683 | 40.27 | 114 | 2.90E-32 | 4.60E-30 |
| 8 | MAFF | 12127 | 136 | 2683 | 30.09 | 88 | 1.10E-26 | 1.60E-24 |
| 9 | ESR1 | 12127 | 69 | 2683 | 15.27 | 56 | 2.20E-25 | 2.80E-23 |
| 10 | COPS5 | 12127 | 136 | 2683 | 30.09 | 86 | 4.70E-25 | 5.30E-23 |
| 11 | EZH2 | 12127 | 56 | 2683 | 12.39 | 47 | 1.00E-22 | 1.00E-20 |
| 12 | MCM2 | 12127 | 62 | 2683 | 13.72 | 49 | 2.00E-21 | 1.90E-19 |
| 13 | GTF2H1 | 12127 | 178 | 2683 | 39.38 | 97 | 3.00E-21 | 2.60E-19 |
| 14 | PPARGC1A | 12127 | 113 | 2683 | 25 | 69 | 3.90E-19 | 3.10E-17 |
| 15 | YEATS2 | 12127 | 88 | 2683 | 19.47 | 58 | 1.40E-18 | 1.00E-16 |
| 16 | PARP1 | 12127 | 52 | 2683 | 11.5 | 41 | 4.70E-18 | 3.30E-16 |
| 17 | TOB1 | 12127 | 132 | 2683 | 29.2 | 74 | 1.90E-17 | 1.20E-15 |
| 18 | FOXK2 | 12127 | 71 | 2683 | 15.71 | 49 | 3.50E-17 | 2.20E-15 |
| 19 | NPM1 | 12127 | 85 | 2683 | 18.81 | 54 | 2.40E-16 | 1.40E-14 |
| 20 | NR0B2 | 12127 | 174 | 2683 | 38.5 | 86 | 1.50E-15 | 8.20E-14 |
| 21 | TCEA2 | 12127 | 114 | 2683 | 25.22 | 64 | 2.60E-15 | 1.40E-13 |
| 22 | POLR2K | 12127 | 87 | 2683 | 19.25 | 53 | 6.00E-15 | 3.00E-13 |
| 23 | TEAD4 | 12127 | 113 | 2683 | 25 | 63 | 6.70E-15 | 3.20E-13 |
| 24 | MLXIPL | 12127 | 270 | 2683 | 59.74 | 116 | 8.90E-15 | 4.20E-13 |
| 25 | NR3C2 | 12127 | 96 | 2683 | 21.24 | 56 | 1.50E-14 | 6.70E-13 |
| 26 | GTF2E1 | 12127 | 100 | 2683 | 22.12 | 57 | 3.50E-14 | 1.50E-12 |
| 27 | AR | 12127 | 111 | 2683 | 24.56 | 61 | 4.40E-14 | 1.80E-12 |
| 28 | ADNP | 12127 | 134 | 2683 | 29.65 | 69 | 7.20E-14 | 2.90E-12 |
| 29 | TRIP13 | 12127 | 55 | 2683 | 12.17 | 38 | 1.20E-13 | 4.70E-12 |
| 30 | ARID4B | 12127 | 71 | 2683 | 15.71 | 44 | 5.10E-13 | 1.90E-11 |
| 31 | TFAP2A | 12127 | 148 | 2683 | 32.74 | 72 | 8.20E-13 | 3.00E-11 |
| 32 | GTF2IRD1 | 12127 | 56 | 2683 | 12.39 | 37 | 2.10E-12 | 7.50E-11 |
| 33 | ELF4 | 12127 | 279 | 2683 | 61.73 | 113 | 2.30E-12 | 7.90E-11 |
| 34 | TAF5L | 12127 | 45 | 2683 | 9.96 | 32 | 3.10E-12 | 1.00E-10 |
| 35 | ENO1 | 12127 | 206 | 2683 | 45.58 | 89 | 8.30E-12 | 2.70E-10 |
| 36 | TRIM22 | 12127 | 126 | 2683 | 27.88 | 62 | 1.70E-11 | 5.30E-10 |
| 37 | TSC22D3 | 12127 | 38 | 2683 | 8.41 | 28 | 1.80E-11 | 5.40E-10 |
| 38 | LDB2 | 12127 | 67 | 2683 | 14.82 | 40 | 3.20E-11 | 9.30E-10 |
| 39 | ZNHIT3 | 12127 | 224 | 2683 | 49.56 | 93 | 4.20E-11 | 1.20E-09 |
| 40 | FOXM1 | 12127 | 53 | 2683 | 11.73 | 34 | 5.70E-11 | 1.60E-09 |
| 41 | DR1 | 12127 | 98 | 2683 | 21.68 | 51 | 7.80E-11 | 2.10E-09 |
| 42 | HLF | 12127 | 87 | 2683 | 19.25 | 47 | 8.00E-11 | 2.10E-09 |
| 43 | HABP4 | 12127 | 66 | 2683 | 14.6 | 39 | 8.70E-11 | 2.30E-09 |
| 44 | POLR2G | 12127 | 59 | 2683 | 13.05 | 36 | 1.30E-10 | 3.30E-09 |
| 45 | CREBL2 | 12127 | 78 | 2683 | 17.26 | 43 | 2.10E-10 | 5.30E-09 |
| 46 | E2F5 | 12127 | 65 | 2683 | 14.38 | 38 | 2.30E-10 | 5.70E-09 |
| 47 | ZNF143 | 12127 | 32 | 2683 | 7.08 | 24 | 2.80E-10 | 6.60E-09 |
| 48 | OVOL2 | 12127 | 143 | 2683 | 31.64 | 65 | 4.20E-10 | 9.70E-09 |
| 49 | HMGB3 | 12127 | 66 | 2683 | 14.6 | 38 | 4.40E-10 | 1.00E-08 |
| 50 | EGR1 | 12127 | 31 | 2683 | 6.86 | 23 | 9.50E-10 | 2.10E-08 |
| 51 | PER1 | 12127 | 27 | 2683 | 5.97 | 21 | 1.20E-09 | 2.60E-08 |
| 52 | CREG1 | 12127 | 105 | 2683 | 23.23 | 51 | 1.90E-09 | 4.10E-08 |
| 53 | CHD7 | 12127 | 56 | 2683 | 12.39 | 33 | 2.70E-09 | 5.70E-08 |
| 54 | HDGF | 12127 | 51 | 2683 | 11.28 | 31 | 2.80E-09 | 5.90E-08 |
| 55 | TAF1A | 12127 | 39 | 2683 | 8.63 | 26 | 3.10E-09 | 6.40E-08 |
| 56 | AEBP1 | 12127 | 194 | 2683 | 42.92 | 79 | 3.50E-09 | 6.80E-08 |
| 57 | ZNF7 | 12127 | 59 | 2683 | 13.05 | 34 | 3.50E-09 | 6.80E-08 |
| 58 | TIMELESS | 12127 | 59 | 2683 | 13.05 | 34 | 3.50E-09 | 6.80E-08 |
| 59 | POGK | 12127 | 87 | 2683 | 19.25 | 44 | 4.90E-09 | 9.40E-08 |
| 60 | CEBPG | 12127 | 90 | 2683 | 19.91 | 45 | 5.30E-09 | 9.90E-08 |
| 61 | PCBD1 | 12127 | 186 | 2683 | 41.15 | 76 | 5.80E-09 | 1.10E-07 |
| 62 | NR4A1 | 12127 | 48 | 2683 | 10.62 | 29 | 1.10E-08 | 2.00E-07 |
| 63 | PPARG | 12127 | 75 | 2683 | 16.59 | 39 | 1.30E-08 | 2.40E-07 |
| 64 | TRIB3 | 12127 | 81 | 2683 | 17.92 | 41 | 1.60E-08 | 2.80E-07 |
| 65 | SSB | 12127 | 59 | 2683 | 13.05 | 33 | 1.70E-08 | 2.90E-07 |
| 66 | BLZF1 | 12127 | 52 | 2683 | 11.5 | 30 | 2.80E-08 | 4.80E-07 |
| 67 | BCL11A | 12127 | 60 | 2683 | 13.27 | 33 | 2.90E-08 | 4.90E-07 |
| 68 | SOX4 | 12127 | 153 | 2683 | 33.85 | 64 | 3.20E-08 | 5.20E-07 |
| 69 | TCF3 | 12127 | 35 | 2683 | 7.74 | 23 | 3.90E-08 | 6.30E-07 |
| 70 | CNOT6 | 12127 | 66 | 2683 | 14.6 | 35 | 3.90E-08 | 6.30E-07 |
| 71 | DEK | 12127 | 50 | 2683 | 11.06 | 29 | 4.10E-08 | 6.50E-07 |
| 72 | HSF2 | 12127 | 16 | 2683 | 3.54 | 14 | 5.00E-08 | 7.70E-07 |
| 73 | EIF5B | 12127 | 57 | 2683 | 12.61 | 31 | 1.10E-07 | 1.70E-06 |
| 74 | ARID3A | 12127 | 151 | 2683 | 33.41 | 62 | 1.20E-07 | 1.80E-06 |
| 75 | TCEA1 | 12127 | 44 | 2683 | 9.73 | 26 | 1.20E-07 | 1.80E-06 |
| 76 | ATF1 | 12127 | 120 | 2683 | 26.55 | 52 | 1.60E-07 | 2.30E-06 |
| 77 | MCM6 | 12127 | 23 | 2683 | 5.09 | 17 | 1.80E-07 | 2.60E-06 |
| 78 | TBC1D2B | 12127 | 50 | 2683 | 11.06 | 28 | 1.90E-07 | 2.80E-06 |
| 79 | TTF2 | 12127 | 45 | 2683 | 9.96 | 26 | 2.30E-07 | 3.20E-06 |
| 80 | GTF3C3 | 12127 | 67 | 2683 | 14.82 | 34 | 2.50E-07 | 3.40E-06 |
| 81 | SNRPB | 12127 | 70 | 2683 | 15.49 | 35 | 2.60E-07 | 3.60E-06 |
| 82 | RBBP4 | 12127 | 103 | 2683 | 22.79 | 46 | 2.80E-07 | 3.80E-06 |
| 83 | E2F8 | 12127 | 26 | 2683 | 5.75 | 18 | 3.70E-07 | 5.00E-06 |
| 84 | TRIM32 | 12127 | 77 | 2683 | 17.04 | 37 | 4.30E-07 | 5.80E-06 |
| 85 | DNMT1 | 12127 | 31 | 2683 | 6.86 | 20 | 4.80E-07 | 6.30E-06 |
| 86 | ZNF623 | 12127 | 31 | 2683 | 6.86 | 20 | 4.80E-07 | 6.30E-06 |
| 87 | RUVBL1 | 12127 | 69 | 2683 | 15.27 | 34 | 6.00E-07 | 7.80E-06 |
| 88 | FOS | 12127 | 29 | 2683 | 6.42 | 19 | 6.60E-07 | 8.40E-06 |
| 89 | ELK4 | 12127 | 39 | 2683 | 8.63 | 23 | 6.90E-07 | 8.60E-06 |
| 90 | XRCC6 | 12127 | 39 | 2683 | 8.63 | 23 | 6.90E-07 | 8.60E-06 |
| 91 | SMAD1 | 12127 | 47 | 2683 | 10.4 | 26 | 7.30E-07 | 9.10E-06 |
| 92 | PER3 | 12127 | 103 | 2683 | 22.79 | 45 | 8.00E-07 | 9.80E-06 |
| 93 | MCM7 | 12127 | 42 | 2683 | 9.29 | 24 | 8.80E-07 | 1.10E-05 |
| 94 | SAP30 | 12127 | 79 | 2683 | 17.48 | 37 | 9.70E-07 | 1.20E-05 |
| 95 | DAB2 | 12127 | 130 | 2683 | 28.76 | 53 | 1.20E-06 | 1.50E-05 |
| 96 | SOX13 | 12127 | 92 | 2683 | 20.35 | 41 | 1.30E-06 | 1.50E-05 |
| 97 | NPAS2 | 12127 | 35 | 2683 | 7.74 | 21 | 1.40E-06 | 1.60E-05 |
| 98 | TCF21 | 12127 | 30 | 2683 | 6.64 | 19 | 1.40E-06 | 1.60E-05 |
| 99 | HMGB2 | 12127 | 38 | 2683 | 8.41 | 22 | 1.90E-06 | 2.10E-05 |
| 100 | TFDP2 | 12127 | 38 | 2683 | 8.41 | 22 | 1.90E-06 | 2.10E-05 |
| 101 | HMGN4 | 12127 | 38 | 2683 | 8.41 | 22 | 1.90E-06 | 2.10E-05 |
| 102 | TBX3 | 12127 | 129 | 2683 | 28.54 | 52 | 2.30E-06 | 2.60E-05 |
| 103 | TRIM27 | 12127 | 58 | 2683 | 12.83 | 29 | 2.70E-06 | 3.00E-05 |
| 104 | ETV1 | 12127 | 31 | 2683 | 6.86 | 19 | 2.90E-06 | 3.10E-05 |
| 105 | CEBPD | 12127 | 53 | 2683 | 11.73 | 27 | 3.80E-06 | 4.10E-05 |
| 106 | PRPF4B | 12127 | 86 | 2683 | 19.03 | 38 | 4.10E-06 | 4.30E-05 |
| 107 | LEF1 | 12127 | 74 | 2683 | 16.37 | 34 | 4.50E-06 | 4.70E-05 |
| 108 | TARBP1 | 12127 | 63 | 2683 | 13.94 | 30 | 6.60E-06 | 6.90E-05 |
| 109 | NFKBIE | 12127 | 49 | 2683 | 10.84 | 25 | 8.40E-06 | 8.60E-05 |
| 110 | BOLA1 | 12127 | 52 | 2683 | 11.5 | 26 | 9.00E-06 | 9.10E-05 |
| 111 | DBP | 12127 | 33 | 2683 | 7.3 | 19 | 1.10E-05 | 1.10E-04 |
| 112 | PHF20 | 12127 | 28 | 2683 | 6.19 | 17 | 1.20E-05 | 1.20E-04 |
| 113 | SSBP3 | 12127 | 47 | 2683 | 10.4 | 24 | 1.20E-05 | 1.20E-04 |
| 114 | MYC | 12127 | 163 | 2683 | 36.06 | 60 | 1.30E-05 | 1.30E-04 |
| 115 | ARNT | 12127 | 50 | 2683 | 11.06 | 25 | 1.30E-05 | 1.30E-04 |
| 116 | ZBTB16 | 12127 | 31 | 2683 | 6.86 | 18 | 1.50E-05 | 1.50E-04 |
| 117 | ZNF239 | 12127 | 26 | 2683 | 5.75 | 16 | 1.70E-05 | 1.60E-04 |
| 118 | DENND4A | 12127 | 57 | 2683 | 12.61 | 27 | 2.10E-05 | 2.00E-04 |
| 119 | GTF3C4 | 12127 | 79 | 2683 | 17.48 | 34 | 2.50E-05 | 2.40E-04 |
| 120 | HMGA1 | 12127 | 32 | 2683 | 7.08 | 18 | 2.80E-05 | 2.60E-04 |
| 121 | MAF | 12127 | 32 | 2683 | 7.08 | 18 | 2.80E-05 | 2.60E-04 |
| 122 | JUNB | 12127 | 70 | 2683 | 15.49 | 31 | 2.90E-05 | 2.70E-04 |
| 123 | PDCD11 | 12127 | 90 | 2683 | 19.91 | 37 | 3.90E-05 | 3.50E-04 |
| 124 | NRBF2 | 12127 | 137 | 2683 | 30.31 | 51 | 3.90E-05 | 3.50E-04 |
| 125 | TRIP4 | 12127 | 41 | 2683 | 9.07 | 21 | 4.10E-05 | 3.60E-04 |
| 126 | ZHX3 | 12127 | 30 | 2683 | 6.64 | 17 | 4.10E-05 | 3.60E-04 |
| 127 | BCL3 | 12127 | 124 | 2683 | 27.43 | 47 | 4.60E-05 | 4.10E-04 |
| 128 | E2F3 | 12127 | 20 | 2683 | 4.42 | 13 | 4.70E-05 | 4.10E-04 |
| 129 | HDAC2 | 12127 | 94 | 2683 | 20.8 | 38 | 4.80E-05 | 4.10E-04 |
| 130 | ZNF74 | 12127 | 36 | 2683 | 7.96 | 19 | 5.60E-05 | 4.80E-04 |
| 131 | FBXO7 | 12127 | 72 | 2683 | 15.93 | 31 | 5.70E-05 | 4.80E-04 |
| 132 | SMAD6 | 12127 | 18 | 2683 | 3.98 | 12 | 6.40E-05 | 5.40E-04 |
| 133 | ZHX2 | 12127 | 18 | 2683 | 3.98 | 12 | 6.40E-05 | 5.40E-04 |
| 134 | IRF6 | 12127 | 102 | 2683 | 22.57 | 40 | 6.90E-05 | 5.70E-04 |
| 135 | KLF9 | 12127 | 31 | 2683 | 6.86 | 17 | 7.30E-05 | 5.90E-04 |
| 136 | IVNS1ABP | 12127 | 31 | 2683 | 6.86 | 17 | 7.30E-05 | 5.90E-04 |
| 137 | SOX9 | 12127 | 61 | 2683 | 13.5 | 27 | 9.50E-05 | 7.70E-04 |
| 138 | GLI3 | 12127 | 40 | 2683 | 8.85 | 20 | 9.60E-05 | 7.80E-04 |
| 139 | SP2 | 12127 | 46 | 2683 | 10.18 | 22 | 1.00E-04 | 8.20E-04 |
| 140 | HIF1A | 12127 | 104 | 2683 | 23.01 | 40 | 1.10E-04 | 9.00E-04 |
| 141 | SCML1 | 12127 | 104 | 2683 | 23.01 | 40 | 1.10E-04 | 9.00E-04 |
| 142 | HAX1 | 12127 | 32 | 2683 | 7.08 | 17 | 1.20E-04 | 9.70E-04 |
| 143 | RFX5 | 12127 | 62 | 2683 | 13.72 | 27 | 1.30E-04 | 1.00E-03 |
| 144 | MYBL1 | 12127 | 19 | 2683 | 4.2 | 12 | 1.40E-04 | 1.10E-03 |
| 145 | RORC | 12127 | 59 | 2683 | 13.05 | 26 | 1.40E-04 | 1.10E-03 |
| 146 | ZNF175 | 12127 | 19 | 2683 | 4.2 | 12 | 1.40E-04 | 1.10E-03 |
| 147 | APEX1 | 12127 | 56 | 2683 | 12.39 | 25 | 1.40E-04 | 1.10E-03 |
| 148 | TARDBP | 12127 | 53 | 2683 | 11.73 | 24 | 1.50E-04 | 1.10E-03 |
| 149 | HEXIM1 | 12127 | 44 | 2683 | 9.73 | 21 | 1.50E-04 | 1.10E-03 |
| 150 | PSIP1 | 12127 | 44 | 2683 | 9.73 | 21 | 1.50E-04 | 1.10E-03 |
| 151 | NR5A2 | 12127 | 63 | 2683 | 13.94 | 27 | 1.80E-04 | 1.30E-03 |
| 152 | RAD51 | 12127 | 30 | 2683 | 6.64 | 16 | 1.80E-04 | 1.30E-03 |
| 153 | BAZ1A | 12127 | 30 | 2683 | 6.64 | 16 | 1.80E-04 | 1.30E-03 |
| 154 | HIC2 | 12127 | 96 | 2683 | 21.24 | 37 | 1.90E-04 | 1.40E-03 |
| 155 | SREBF2 | 12127 | 54 | 2683 | 11.95 | 24 | 2.10E-04 | 1.50E-03 |
| 156 | PITX1 | 12127 | 48 | 2683 | 10.62 | 22 | 2.20E-04 | 1.60E-03 |
| 157 | ZBTB10 | 12127 | 42 | 2683 | 9.29 | 20 | 2.30E-04 | 1.60E-03 |
| 158 | ZNF146 | 12127 | 25 | 2683 | 5.53 | 14 | 2.40E-04 | 1.70E-03 |
| 159 | PDLIM1 | 12127 | 25 | 2683 | 5.53 | 14 | 2.40E-04 | 1.70E-03 |
| 160 | SALL1 | 12127 | 97 | 2683 | 21.46 | 37 | 2.50E-04 | 1.70E-03 |
| 161 | KLF12 | 12127 | 52 | 2683 | 11.5 | 23 | 3.10E-04 | 2.20E-03 |
| 162 | NFIC | 12127 | 49 | 2683 | 10.84 | 22 | 3.20E-04 | 2.20E-03 |
| 163 | SRF | 12127 | 49 | 2683 | 10.84 | 22 | 3.20E-04 | 2.20E-03 |
| 164 | TSHZ2 | 12127 | 34 | 2683 | 7.52 | 17 | 3.20E-04 | 2.20E-03 |
| 165 | PIAS3 | 12127 | 40 | 2683 | 8.85 | 19 | 3.40E-04 | 2.30E-03 |
| 166 | BRCA1 | 12127 | 23 | 2683 | 5.09 | 13 | 3.50E-04 | 2.40E-03 |
| 167 | SIRT3 | 12127 | 99 | 2683 | 21.9 | 37 | 3.90E-04 | 2.60E-03 |
| 168 | CHAF1A | 12127 | 18 | 2683 | 3.98 | 11 | 4.00E-04 | 2.70E-03 |
| 169 | TRIM31 | 12127 | 26 | 2683 | 5.75 | 14 | 4.10E-04 | 2.70E-03 |
| 170 | HTATSF1 | 12127 | 69 | 2683 | 15.27 | 28 | 4.20E-04 | 2.80E-03 |
| 171 | POLR3C | 12127 | 29 | 2683 | 6.42 | 15 | 4.50E-04 | 2.90E-03 |
| 172 | RLF | 12127 | 41 | 2683 | 9.07 | 19 | 5.00E-04 | 3.20E-03 |
| 173 | TLE3 | 12127 | 41 | 2683 | 9.07 | 19 | 5.00E-04 | 3.20E-03 |
| 174 | SREBF1 | 12127 | 57 | 2683 | 12.61 | 24 | 5.60E-04 | 3.60E-03 |
| 175 | RAI14 | 12127 | 27 | 2683 | 5.97 | 14 | 6.80E-04 | 4.30E-03 |
| 176 | DIP2C | 12127 | 42 | 2683 | 9.29 | 19 | 7.20E-04 | 4.60E-03 |
| 177 | PAPOLA | 12127 | 99 | 2683 | 21.9 | 36 | 8.50E-04 | 5.40E-03 |
| 178 | POLR2B | 12127 | 65 | 2683 | 14.38 | 26 | 8.70E-04 | 5.40E-03 |
| 179 | POU2F1 | 12127 | 49 | 2683 | 10.84 | 21 | 9.30E-04 | 5.80E-03 |
| 180 | ILF3 | 12127 | 62 | 2683 | 13.72 | 25 | 9.40E-04 | 5.80E-03 |
| 181 | WWP1 | 12127 | 79 | 2683 | 17.48 | 30 | 1.00E-03 | 6.20E-03 |
| 182 | NFYA | 12127 | 28 | 2683 | 6.19 | 14 | 1.10E-03 | 6.60E-03 |
| 183 | FHL2 | 12127 | 37 | 2683 | 8.19 | 17 | 1.10E-03 | 6.70E-03 |
| 184 | SAP30BP | 12127 | 115 | 2683 | 25.44 | 40 | 1.20E-03 | 7.50E-03 |
| 185 | ARID4A | 12127 | 70 | 2683 | 15.49 | 27 | 1.30E-03 | 8.00E-03 |
| 186 | HTATIP2 | 12127 | 119 | 2683 | 26.33 | 41 | 1.30E-03 | 8.00E-03 |
| 187 | RNF6 | 12127 | 84 | 2683 | 18.58 | 31 | 1.40E-03 | 8.50E-03 |
| 188 | ECD | 12127 | 84 | 2683 | 18.58 | 31 | 1.40E-03 | 8.50E-03 |
| 189 | RFC1 | 12127 | 44 | 2683 | 9.73 | 19 | 1.40E-03 | 8.50E-03 |
| 190 | DPF2 | 12127 | 57 | 2683 | 12.61 | 23 | 1.50E-03 | 8.60E-03 |
| 191 | JUND | 12127 | 38 | 2683 | 8.41 | 17 | 1.60E-03 | 9.20E-03 |
| 192 | KLF10 | 12127 | 38 | 2683 | 8.41 | 17 | 1.60E-03 | 9.20E-03 |
| 193 | GATA2 | 12127 | 35 | 2683 | 7.74 | 16 | 1.60E-03 | 9.50E-03 |
| 194 | ARID5B | 12127 | 122 | 2683 | 26.99 | 41 | 2.30E-03 | 1.30E-02 |
| 195 | ZNF84 | 12127 | 21 | 2683 | 4.65 | 11 | 2.30E-03 | 1.30E-02 |
| 196 | NR1H3 | 12127 | 66 | 2683 | 14.6 | 25 | 2.60E-03 | 1.50E-02 |
| 197 | SP3 | 12127 | 56 | 2683 | 12.39 | 22 | 2.80E-03 | 1.60E-02 |
| 198 | SNAI2 | 12127 | 16 | 2683 | 3.54 | 9 | 3.10E-03 | 1.70E-02 |
| 199 | ETV5 | 12127 | 37 | 2683 | 8.19 | 16 | 3.30E-03 | 1.80E-02 |
| 200 | KNTC1 | 12127 | 37 | 2683 | 8.19 | 16 | 3.30E-03 | 1.80E-02 |
| 201 | EHMT2 | 12127 | 50 | 2683 | 11.06 | 20 | 3.30E-03 | 1.80E-02 |
| 202 | GTF2A2 | 12127 | 106 | 2683 | 23.45 | 36 | 3.30E-03 | 1.80E-02 |
| 203 | RNF4 | 12127 | 67 | 2683 | 14.82 | 25 | 3.30E-03 | 1.80E-02 |
| 204 | PRDM4 | 12127 | 28 | 2683 | 6.19 | 13 | 3.70E-03 | 2.00E-02 |
| 205 | DRAP1 | 12127 | 25 | 2683 | 5.53 | 12 | 3.80E-03 | 2.10E-02 |
| 206 | NFKBIA | 12127 | 41 | 2683 | 9.07 | 17 | 4.20E-03 | 2.30E-02 |
| 207 | PHF7 | 12127 | 65 | 2683 | 14.38 | 24 | 4.70E-03 | 2.50E-02 |
| 208 | SCMH1 | 12127 | 35 | 2683 | 7.74 | 15 | 4.90E-03 | 2.60E-02 |
| 209 | NCOA6 | 12127 | 35 | 2683 | 7.74 | 15 | 4.90E-03 | 2.60E-02 |
| 210 | ZBTB20 | 12127 | 76 | 2683 | 16.81 | 27 | 5.20E-03 | 2.80E-02 |
| 211 | CBFB | 12127 | 62 | 2683 | 13.72 | 23 | 5.20E-03 | 2.80E-02 |
| 212 | MYCN | 12127 | 29 | 2683 | 6.42 | 13 | 5.40E-03 | 2.90E-02 |
| 213 | TRIM28 | 12127 | 87 | 2683 | 19.25 | 30 | 5.50E-03 | 2.90E-02 |
| 214 | ZNF131 | 12127 | 20 | 2683 | 4.42 | 10 | 5.60E-03 | 2.90E-02 |
| 215 | ASXL1 | 12127 | 23 | 2683 | 5.09 | 11 | 5.70E-03 | 3.00E-02 |
| 216 | SATB2 | 12127 | 59 | 2683 | 13.05 | 22 | 5.80E-03 | 3.00E-02 |
| 217 | TAF6 | 12127 | 49 | 2683 | 10.84 | 19 | 6.20E-03 | 3.20E-02 |
| 218 | MYB | 12127 | 56 | 2683 | 12.39 | 21 | 6.40E-03 | 3.30E-02 |
| 219 | FMR1 | 12127 | 63 | 2683 | 13.94 | 23 | 6.50E-03 | 3.30E-02 |
| 220 | TAF5 | 12127 | 53 | 2683 | 11.73 | 20 | 7.10E-03 | 3.60E-02 |
| 221 | TCF4 | 12127 | 43 | 2683 | 9.51 | 17 | 7.50E-03 | 3.80E-02 |
| 222 | TRIM24 | 12127 | 43 | 2683 | 9.51 | 17 | 7.50E-03 | 3.80E-02 |
| 223 | RBBP7 | 12127 | 30 | 2683 | 6.64 | 13 | 7.60E-03 | 3.80E-02 |
| 224 | TRIM33 | 12127 | 27 | 2683 | 5.97 | 12 | 8.10E-03 | 4.00E-02 |
| 225 | CHD1 | 12127 | 93 | 2683 | 20.58 | 31 | 8.30E-03 | 4.10E-02 |
| 226 | RORA | 12127 | 18 | 2683 | 3.98 | 9 | 8.50E-03 | 4.20E-02 |
| 227 | ZXDC | 12127 | 18 | 2683 | 3.98 | 9 | 8.50E-03 | 4.20E-02 |
| 228 | PHF21A | 12127 | 75 | 2683 | 16.59 | 26 | 8.60E-03 | 4.20E-02 |
| 229 | GATAD2A | 12127 | 47 | 2683 | 10.4 | 18 | 8.80E-03 | 4.30E-02 |
| 230 | DDIT3 | 12127 | 68 | 2683 | 15.04 | 24 | 8.90E-03 | 4.30E-02 |
| 231 | SMARCC1 | 12127 | 61 | 2683 | 13.5 | 22 | 9.00E-03 | 4.40E-02 |
| 232 | BBX | 12127 | 79 | 2683 | 17.48 | 27 | 9.30E-03 | 4.50E-02 |
| 233 | CEBPA | 12127 | 135 | 2683 | 29.87 | 42 | 9.40E-03 | 4.50E-02 |
| 234 | ZNF165 | 12127 | 83 | 2683 | 18.36 | 28 | 9.90E-03 | 4.70E-02 |
| 235 | JARID2 | 12127 | 31 | 2683 | 6.86 | 13 | 1.10E-02 | 5.00E-02 |

**MRA analysis on the filtered transcriptional network from GSE36376**

| MRA rank2 | Regulon | Universe Size3 | Regulon Size4 | Total Hits5 | Expected Hits6 | Observed Hits7 | P-value | Adjusted P-value |
| --- | --- | --- | --- | --- | --- | --- | --- | --- |
| 1 | NR1I3 | 30346 | 290 | 2368 | 22.63 | 144 | 7.10E-81 | 6.40E-78 |
| 2 | MCM2 | 30346 | 129 | 2368 | 10.07 | 79 | 3.50E-54 | 1.60E-51 |
| 3 | ENO1 | 30346 | 246 | 2368 | 19.2 | 99 | 1.90E-45 | 5.90E-43 |
| 4 | PARP1 | 30346 | 94 | 2368 | 7.34 | 61 | 2.50E-44 | 5.60E-42 |
| 5 | ILF2 | 30346 | 115 | 2368 | 8.97 | 67 | 3.90E-44 | 6.10E-42 |
| 6 | DEDD | 30346 | 65 | 2368 | 5.07 | 51 | 4.00E-44 | 6.10E-42 |
| 7 | POGK | 30346 | 72 | 2368 | 5.62 | 53 | 3.00E-43 | 4.00E-41 |
| 8 | ZNF207 | 30346 | 92 | 2368 | 7.18 | 59 | 1.80E-42 | 2.00E-40 |
| 9 | SEC14L2 | 30346 | 273 | 2368 | 21.3 | 98 | 7.10E-40 | 7.20E-38 |
| 10 | CHD7 | 30346 | 80 | 2368 | 6.24 | 51 | 1.10E-36 | 9.60E-35 |
| 11 | ARID3A | 30346 | 273 | 2368 | 21.3 | 94 | 1.40E-36 | 1.20E-34 |
| 12 | POLR2H | 30346 | 94 | 2368 | 7.34 | 54 | 2.20E-35 | 1.70E-33 |
| 13 | POLR3C | 30346 | 84 | 2368 | 6.55 | 51 | 3.70E-35 | 2.60E-33 |
| 14 | KLF9 | 30346 | 101 | 2368 | 7.88 | 55 | 2.50E-34 | 1.60E-32 |
| 15 | LDB2 | 30346 | 169 | 2368 | 13.19 | 70 | 1.90E-33 | 1.20E-31 |
| 16 | SMARCD1 | 30346 | 109 | 2368 | 8.51 | 52 | 7.70E-29 | 4.40E-27 |
| 17 | HOXC4 | 30346 | 95 | 2368 | 7.41 | 48 | 3.60E-28 | 1.90E-26 |
| 18 | ZNF189 | 30346 | 64 | 2368 | 4.99 | 39 | 2.70E-27 | 1.40E-25 |
| 19 | BUD31 | 30346 | 88 | 2368 | 6.87 | 45 | 8.70E-27 | 4.20E-25 |
| 20 | ID2 | 30346 | 82 | 2368 | 6.4 | 43 | 3.00E-26 | 1.30E-24 |
| 21 | ETV4 | 30346 | 86 | 2368 | 6.71 | 44 | 3.10E-26 | 1.30E-24 |
| 22 | AATF | 30346 | 75 | 2368 | 5.85 | 41 | 4.90E-26 | 2.00E-24 |
| 23 | HEXIM1 | 30346 | 101 | 2368 | 7.88 | 46 | 1.40E-24 | 5.40E-23 |
| 24 | HDAC2 | 30346 | 168 | 2368 | 13.11 | 59 | 5.80E-24 | 2.20E-22 |
| 25 | NR1I2 | 30346 | 93 | 2368 | 7.26 | 43 | 2.10E-23 | 7.60E-22 |
| 26 | APEX1 | 30346 | 151 | 2368 | 11.78 | 55 | 2.60E-23 | 9.00E-22 |
| 27 | TRIB3 | 30346 | 81 | 2368 | 6.32 | 40 | 3.20E-23 | 1.10E-21 |
| 28 | HLF | 30346 | 152 | 2368 | 11.86 | 55 | 3.70E-23 | 1.20E-21 |
| 29 | PTTG1 | 30346 | 100 | 2368 | 7.8 | 44 | 7.90E-23 | 2.50E-21 |
| 30 | PRDM4 | 30346 | 49 | 2368 | 3.82 | 31 | 1.10E-22 | 3.30E-21 |
| 31 | POLR2G | 30346 | 56 | 2368 | 4.37 | 33 | 1.20E-22 | 3.50E-21 |
| 32 | SMARCC1 | 30346 | 115 | 2368 | 8.97 | 46 | 1.10E-21 | 3.00E-20 |
| 33 | TGIF2 | 30346 | 101 | 2368 | 7.88 | 43 | 1.20E-21 | 3.30E-20 |
| 34 | RORC | 30346 | 136 | 2368 | 10.61 | 50 | 1.50E-21 | 4.10E-20 |
| 35 | COPS5 | 30346 | 103 | 2368 | 8.04 | 43 | 3.00E-21 | 7.80E-20 |
| 36 | TBC1D2B | 30346 | 165 | 2368 | 12.88 | 55 | 3.40E-21 | 8.50E-20 |
| 37 | POLR1C | 30346 | 68 | 2368 | 5.31 | 34 | 3.50E-20 | 8.70E-19 |
| 38 | TTF2 | 30346 | 90 | 2368 | 7.02 | 39 | 4.20E-20 | 1.00E-18 |
| 39 | TRIM28 | 30346 | 82 | 2368 | 6.4 | 37 | 6.90E-20 | 1.60E-18 |
| 40 | ESR1 | 30346 | 59 | 2368 | 4.6 | 31 | 2.40E-19 | 5.50E-18 |
| 41 | DNMT3L | 30346 | 119 | 2368 | 9.29 | 44 | 3.10E-19 | 6.80E-18 |
| 42 | SUZ12 | 30346 | 73 | 2368 | 5.7 | 34 | 6.30E-19 | 1.40E-17 |
| 43 | KLF2 | 30346 | 57 | 2368 | 4.45 | 30 | 8.60E-19 | 1.80E-17 |
| 44 | SNRPB | 30346 | 62 | 2368 | 4.84 | 31 | 1.60E-18 | 3.30E-17 |
| 45 | HDAC1 | 30346 | 34 | 2368 | 2.65 | 23 | 3.70E-18 | 7.50E-17 |
| 46 | SUPT16H | 30346 | 30 | 2368 | 2.34 | 21 | 3.60E-17 | 7.20E-16 |
| 47 | ZNF7 | 30346 | 40 | 2368 | 3.12 | 24 | 4.30E-17 | 8.30E-16 |
| 48 | SUPT5H | 30346 | 45 | 2368 | 3.51 | 25 | 1.20E-16 | 2.30E-15 |
| 49 | RBM14 | 30346 | 139 | 2368 | 10.85 | 44 | 2.70E-16 | 5.00E-15 |
| 50 | E2F6 | 30346 | 36 | 2368 | 2.81 | 22 | 5.00E-16 | 9.20E-15 |
| 51 | GTF3C2 | 30346 | 52 | 2368 | 4.06 | 26 | 9.30E-16 | 1.70E-14 |
| 52 | MYB | 30346 | 27 | 2368 | 2.11 | 19 | 1.00E-15 | 1.80E-14 |
| 53 | DAB2 | 30346 | 133 | 2368 | 10.38 | 42 | 1.40E-15 | 2.40E-14 |
| 54 | PWP1 | 30346 | 81 | 2368 | 6.32 | 32 | 2.40E-15 | 4.00E-14 |
| 55 | NAP1L4 | 30346 | 42 | 2368 | 3.28 | 23 | 3.10E-15 | 5.20E-14 |
| 56 | TRIP13 | 30346 | 77 | 2368 | 6.01 | 31 | 3.50E-15 | 5.60E-14 |
| 57 | BARD1 | 30346 | 73 | 2368 | 5.7 | 30 | 4.90E-15 | 7.90E-14 |
| 58 | MLXIPL | 30346 | 214 | 2368 | 16.7 | 54 | 6.60E-15 | 1.00E-13 |
| 59 | PAPOLA | 30346 | 106 | 2368 | 8.27 | 36 | 1.20E-14 | 1.80E-13 |
| 60 | PPARGC1A | 30346 | 48 | 2368 | 3.75 | 24 | 1.20E-14 | 1.80E-13 |
| 61 | HBP1 | 30346 | 123 | 2368 | 9.6 | 39 | 1.20E-14 | 1.80E-13 |
| 62 | KNTC1 | 30346 | 49 | 2368 | 3.82 | 24 | 2.20E-14 | 3.20E-13 |
| 63 | E2F2 | 30346 | 72 | 2368 | 5.62 | 29 | 2.60E-14 | 3.80E-13 |
| 64 | WWP1 | 30346 | 82 | 2368 | 6.4 | 31 | 2.70E-14 | 3.90E-13 |
| 65 | DPF2 | 30346 | 34 | 2368 | 2.65 | 20 | 3.10E-14 | 4.40E-13 |
| 66 | ELF3 | 30346 | 84 | 2368 | 6.55 | 31 | 5.90E-14 | 8.20E-13 |
| 67 | ZNF142 | 30346 | 85 | 2368 | 6.63 | 31 | 8.60E-14 | 1.20E-12 |
| 68 | DDIT3 | 30346 | 44 | 2368 | 3.43 | 22 | 1.50E-13 | 2.00E-12 |
| 69 | PHF21A | 30346 | 44 | 2368 | 3.43 | 22 | 1.50E-13 | 2.00E-12 |
| 70 | SOX13 | 30346 | 72 | 2368 | 5.62 | 28 | 2.10E-13 | 2.70E-12 |
| 71 | PIR | 30346 | 116 | 2368 | 9.05 | 36 | 2.60E-13 | 3.40E-12 |
| 72 | POLR1D | 30346 | 111 | 2368 | 8.66 | 35 | 3.30E-13 | 4.20E-12 |
| 73 | TAF1A | 30346 | 89 | 2368 | 6.94 | 31 | 3.60E-13 | 4.50E-12 |
| 74 | NR4A2 | 30346 | 30 | 2368 | 2.34 | 18 | 3.80E-13 | 4.60E-12 |
| 75 | SND1 | 30346 | 46 | 2368 | 3.59 | 22 | 4.90E-13 | 5.90E-12 |
| 76 | HDAC11 | 30346 | 34 | 2368 | 2.65 | 19 | 5.00E-13 | 6.00E-12 |
| 77 | RGS14 | 30346 | 60 | 2368 | 4.68 | 25 | 6.30E-13 | 7.50E-12 |
| 78 | SAFB2 | 30346 | 80 | 2368 | 6.24 | 29 | 6.50E-13 | 7.60E-12 |
| 79 | KLF15 | 30346 | 167 | 2368 | 13.03 | 43 | 1.70E-12 | 2.00E-11 |
| 80 | CTDSPL | 30346 | 53 | 2368 | 4.14 | 23 | 1.90E-12 | 2.10E-11 |
| 81 | SOX9 | 30346 | 94 | 2368 | 7.34 | 31 | 1.90E-12 | 2.20E-11 |
| 82 | BCL11A | 30346 | 118 | 2368 | 9.21 | 35 | 2.50E-12 | 2.70E-11 |
| 83 | SMARCB1 | 30346 | 79 | 2368 | 6.16 | 28 | 3.10E-12 | 3.40E-11 |
| 84 | EPAS1 | 30346 | 86 | 2368 | 6.71 | 29 | 5.30E-12 | 5.70E-11 |
| 85 | NFE2 | 30346 | 60 | 2368 | 4.68 | 24 | 5.30E-12 | 5.70E-11 |
| 86 | MSC | 30346 | 65 | 2368 | 5.07 | 25 | 5.40E-12 | 5.70E-11 |
| 87 | HSF2 | 30346 | 31 | 2368 | 2.42 | 17 | 1.30E-11 | 1.30E-10 |
| 88 | NR5A2 | 30346 | 107 | 2368 | 8.35 | 32 | 1.70E-11 | 1.70E-10 |
| 89 | KHSRP | 30346 | 68 | 2368 | 5.31 | 25 | 1.70E-11 | 1.80E-10 |
| 90 | REXO4 | 30346 | 32 | 2368 | 2.5 | 17 | 2.50E-11 | 2.60E-10 |
| 91 | GTF3C3 | 30346 | 60 | 2368 | 4.68 | 23 | 4.10E-11 | 4.10E-10 |
| 92 | TRIM31 | 30346 | 33 | 2368 | 2.58 | 17 | 4.80E-11 | 4.80E-10 |
| 93 | TAX1BP3 | 30346 | 130 | 2368 | 10.14 | 35 | 5.10E-11 | 5.00E-10 |
| 94 | GMEB2 | 30346 | 89 | 2368 | 6.94 | 28 | 8.00E-11 | 7.70E-10 |
| 95 | ZSCAN2 | 30346 | 47 | 2368 | 3.67 | 20 | 8.10E-11 | 7.70E-10 |
| 96 | COIL | 30346 | 35 | 2368 | 2.73 | 17 | 1.60E-10 | 1.50E-09 |
| 97 | RNF4 | 30346 | 49 | 2368 | 3.82 | 20 | 2.00E-10 | 1.90E-09 |
| 98 | RBMX | 30346 | 54 | 2368 | 4.21 | 21 | 2.10E-10 | 2.00E-09 |
| 99 | MXD3 | 30346 | 36 | 2368 | 2.81 | 17 | 2.80E-10 | 2.60E-09 |
| 100 | ELF4 | 30346 | 208 | 2368 | 16.23 | 45 | 3.00E-10 | 2.70E-09 |
| 101 | TIMELESS | 30346 | 60 | 2368 | 4.68 | 22 | 3.00E-10 | 2.70E-09 |
| 102 | SMAD6 | 30346 | 55 | 2368 | 4.29 | 21 | 3.10E-10 | 2.80E-09 |
| 103 | KHDRBS1 | 30346 | 106 | 2368 | 8.27 | 30 | 3.20E-10 | 2.80E-09 |
| 104 | SOX4 | 30346 | 100 | 2368 | 7.8 | 29 | 3.30E-10 | 2.90E-09 |
| 105 | E2F3 | 30346 | 127 | 2368 | 9.91 | 33 | 5.00E-10 | 4.30E-09 |
| 106 | MYCN | 30346 | 84 | 2368 | 6.55 | 26 | 5.60E-10 | 4.80E-09 |
| 107 | IKBKG | 30346 | 33 | 2368 | 2.58 | 16 | 5.80E-10 | 5.00E-09 |
| 108 | E2F5 | 30346 | 68 | 2368 | 5.31 | 23 | 7.60E-10 | 6.40E-09 |
| 109 | SSB | 30346 | 43 | 2368 | 3.36 | 18 | 9.90E-10 | 8.20E-09 |
| 110 | CEBPA | 30346 | 76 | 2368 | 5.93 | 24 | 1.60E-09 | 1.30E-08 |
| 111 | ZHX2 | 30346 | 40 | 2368 | 3.12 | 17 | 2.20E-09 | 1.80E-08 |
| 112 | ARNT2 | 30346 | 61 | 2368 | 4.76 | 21 | 2.90E-09 | 2.30E-08 |
| 113 | NFE2L3 | 30346 | 129 | 2368 | 10.07 | 32 | 3.20E-09 | 2.50E-08 |
| 114 | TSHZ2 | 30346 | 74 | 2368 | 5.77 | 23 | 5.00E-09 | 4.00E-08 |
| 115 | CBX4 | 30346 | 21 | 2368 | 1.64 | 12 | 7.50E-09 | 5.90E-08 |
| 116 | GTF3C5 | 30346 | 48 | 2368 | 3.75 | 18 | 8.10E-09 | 6.40E-08 |
| 117 | HDGF | 30346 | 25 | 2368 | 1.95 | 13 | 8.20E-09 | 6.40E-08 |
| 118 | MAFB | 30346 | 34 | 2368 | 2.65 | 15 | 1.00E-08 | 8.00E-08 |
| 119 | CEBPG | 30346 | 39 | 2368 | 3.04 | 16 | 1.20E-08 | 9.20E-08 |
| 120 | NR1H2 | 30346 | 109 | 2368 | 8.51 | 28 | 1.30E-08 | 9.90E-08 |
| 121 | YEATS2 | 30346 | 110 | 2368 | 8.58 | 28 | 1.60E-08 | 1.20E-07 |
| 122 | GON4L | 30346 | 15 | 2368 | 1.17 | 10 | 1.70E-08 | 1.30E-07 |
| 123 | EZH2 | 30346 | 36 | 2368 | 2.81 | 15 | 2.70E-08 | 2.00E-07 |
| 124 | CEBPD | 30346 | 27 | 2368 | 2.11 | 13 | 2.70E-08 | 2.00E-07 |
| 125 | GTF2E2 | 30346 | 75 | 2368 | 5.85 | 22 | 3.50E-08 | 2.50E-07 |
| 126 | TRRAP | 30346 | 32 | 2368 | 2.5 | 14 | 3.70E-08 | 2.70E-07 |
| 127 | GTF2A2 | 30346 | 274 | 2368 | 21.38 | 49 | 3.90E-08 | 2.80E-07 |
| 128 | HOXA10 | 30346 | 16 | 2368 | 1.25 | 10 | 4.20E-08 | 3.00E-07 |
| 129 | RXRA | 30346 | 70 | 2368 | 5.46 | 21 | 4.60E-08 | 3.20E-07 |
| 130 | MCM6 | 30346 | 20 | 2368 | 1.56 | 11 | 5.50E-08 | 3.90E-07 |
| 131 | POU2F1 | 30346 | 137 | 2368 | 10.69 | 31 | 5.60E-08 | 3.90E-07 |
| 132 | RBBP7 | 30346 | 123 | 2368 | 9.6 | 29 | 5.70E-08 | 3.90E-07 |
| 133 | STAT1 | 30346 | 96 | 2368 | 7.49 | 25 | 5.80E-08 | 4.00E-07 |
| 134 | BAZ1A | 30346 | 33 | 2368 | 2.58 | 14 | 5.90E-08 | 4.00E-07 |
| 135 | BBX | 30346 | 38 | 2368 | 2.97 | 15 | 6.40E-08 | 4.30E-07 |
| 136 | BAZ1B | 30346 | 17 | 2368 | 1.33 | 10 | 9.60E-08 | 6.40E-07 |
| 137 | PIAS4 | 30346 | 73 | 2368 | 5.7 | 21 | 1.00E-07 | 6.80E-07 |
| 138 | MED6 | 30346 | 50 | 2368 | 3.9 | 17 | 1.10E-07 | 7.50E-07 |
| 139 | CHD8 | 30346 | 226 | 2368 | 17.64 | 42 | 1.20E-07 | 7.80E-07 |
| 140 | ATF5 | 30346 | 45 | 2368 | 3.51 | 16 | 1.30E-07 | 8.50E-07 |
| 141 | KLF12 | 30346 | 62 | 2368 | 4.84 | 19 | 1.40E-07 | 8.80E-07 |
| 142 | RXRB | 30346 | 51 | 2368 | 3.98 | 17 | 1.60E-07 | 1.00E-06 |
| 143 | ONECUT2 | 30346 | 57 | 2368 | 4.45 | 18 | 1.70E-07 | 1.10E-06 |
| 144 | ZNF22 | 30346 | 57 | 2368 | 4.45 | 18 | 1.70E-07 | 1.10E-06 |
| 145 | CHAF1A | 30346 | 46 | 2368 | 3.59 | 16 | 1.90E-07 | 1.20E-06 |
| 146 | MEF2D | 30346 | 22 | 2368 | 1.72 | 11 | 2.00E-07 | 1.30E-06 |
| 147 | ATF7IP | 30346 | 41 | 2368 | 3.2 | 15 | 2.10E-07 | 1.30E-06 |
| 148 | ZNF518A | 30346 | 41 | 2368 | 3.2 | 15 | 2.10E-07 | 1.30E-06 |
| 149 | CNOT6 | 30346 | 36 | 2368 | 2.81 | 14 | 2.20E-07 | 1.30E-06 |
| 150 | DEK | 30346 | 110 | 2368 | 8.58 | 26 | 2.60E-07 | 1.60E-06 |
| 151 | RING1 | 30346 | 65 | 2368 | 5.07 | 19 | 3.10E-07 | 1.90E-06 |
| 152 | KLF6 | 30346 | 15 | 2368 | 1.17 | 9 | 3.40E-07 | 2.10E-06 |
| 153 | PLAGL2 | 30346 | 72 | 2368 | 5.62 | 20 | 3.90E-07 | 2.30E-06 |
| 154 | AHCTF1 | 30346 | 54 | 2368 | 4.21 | 17 | 4.00E-07 | 2.40E-06 |
| 155 | AEBP1 | 30346 | 180 | 2368 | 14.05 | 35 | 4.30E-07 | 2.50E-06 |
| 156 | SSX1 | 30346 | 92 | 2368 | 7.18 | 23 | 4.30E-07 | 2.50E-06 |
| 157 | YBX1 | 30346 | 143 | 2368 | 11.16 | 30 | 5.20E-07 | 3.00E-06 |
| 158 | OVOL2 | 30346 | 136 | 2368 | 10.61 | 29 | 5.60E-07 | 3.20E-06 |
| 159 | XRCC6 | 30346 | 24 | 2368 | 1.87 | 11 | 6.10E-07 | 3.50E-06 |
| 160 | SMARCA4 | 30346 | 50 | 2368 | 3.9 | 16 | 6.80E-07 | 3.90E-06 |
| 161 | SMAD3 | 30346 | 34 | 2368 | 2.65 | 13 | 7.50E-07 | 4.20E-06 |
| 162 | HMGB2 | 30346 | 34 | 2368 | 2.65 | 13 | 7.50E-07 | 4.20E-06 |
| 163 | SUB1 | 30346 | 82 | 2368 | 6.4 | 21 | 8.80E-07 | 4.90E-06 |
| 164 | ETV5 | 30346 | 51 | 2368 | 3.98 | 16 | 9.20E-07 | 5.10E-06 |
| 165 | FOS | 30346 | 40 | 2368 | 3.12 | 14 | 9.90E-07 | 5.50E-06 |
| 166 | ZNF593 | 30346 | 83 | 2368 | 6.48 | 21 | 1.10E-06 | 6.00E-06 |
| 167 | ZNF146 | 30346 | 104 | 2368 | 8.12 | 24 | 1.20E-06 | 6.40E-06 |
| 168 | CIC | 30346 | 58 | 2368 | 4.53 | 17 | 1.20E-06 | 6.70E-06 |
| 169 | TARBP1 | 30346 | 84 | 2368 | 6.55 | 21 | 1.30E-06 | 7.20E-06 |
| 170 | RORA | 30346 | 41 | 2368 | 3.2 | 14 | 1.40E-06 | 7.50E-06 |
| 171 | MCM3 | 30346 | 17 | 2368 | 1.33 | 9 | 1.40E-06 | 7.70E-06 |
| 172 | SMARCE1 | 30346 | 31 | 2368 | 2.42 | 12 | 1.70E-06 | 9.00E-06 |
| 173 | SSRP1 | 30346 | 31 | 2368 | 2.42 | 12 | 1.70E-06 | 9.00E-06 |
| 174 | FOXJ1 | 30346 | 37 | 2368 | 2.89 | 13 | 2.30E-06 | 1.20E-05 |
| 175 | DNMT1 | 30346 | 67 | 2368 | 5.23 | 18 | 2.50E-06 | 1.30E-05 |
| 176 | HSF1 | 30346 | 32 | 2368 | 2.5 | 12 | 2.60E-06 | 1.30E-05 |
| 177 | MSX1 | 30346 | 43 | 2368 | 3.36 | 14 | 2.70E-06 | 1.40E-05 |
| 178 | MED8 | 30346 | 43 | 2368 | 3.36 | 14 | 2.70E-06 | 1.40E-05 |
| 179 | IRF6 | 30346 | 49 | 2368 | 3.82 | 15 | 2.80E-06 | 1.40E-05 |
| 180 | HMGN1 | 30346 | 55 | 2368 | 4.29 | 16 | 2.80E-06 | 1.40E-05 |
| 181 | TARBP2 | 30346 | 49 | 2368 | 3.82 | 15 | 2.80E-06 | 1.40E-05 |
| 182 | NFIB | 30346 | 81 | 2368 | 6.32 | 20 | 2.90E-06 | 1.50E-05 |
| 183 | PHTF1 | 30346 | 95 | 2368 | 7.41 | 22 | 3.00E-06 | 1.50E-05 |
| 184 | EGR1 | 30346 | 38 | 2368 | 2.97 | 13 | 3.20E-06 | 1.60E-05 |
| 185 | FOXM1 | 30346 | 23 | 2368 | 1.79 | 10 | 3.60E-06 | 1.80E-05 |
| 186 | BATF | 30346 | 33 | 2368 | 2.58 | 12 | 3.70E-06 | 1.80E-05 |
| 187 | ETS2 | 30346 | 50 | 2368 | 3.9 | 15 | 3.80E-06 | 1.80E-05 |
| 188 | DMTF1 | 30346 | 19 | 2368 | 1.48 | 9 | 4.70E-06 | 2.30E-05 |
| 189 | HOXD1 | 30346 | 57 | 2368 | 4.45 | 16 | 4.80E-06 | 2.30E-05 |
| 190 | ZNF281 | 30346 | 45 | 2368 | 3.51 | 14 | 4.90E-06 | 2.40E-05 |
| 191 | CHD4 | 30346 | 64 | 2368 | 4.99 | 17 | 5.50E-06 | 2.60E-05 |
| 192 | PIAS1 | 30346 | 64 | 2368 | 4.99 | 17 | 5.50E-06 | 2.60E-05 |
| 193 | SUPT4H1 | 30346 | 78 | 2368 | 6.09 | 19 | 6.40E-06 | 3.00E-05 |
| 194 | PDLIM1 | 30346 | 46 | 2368 | 3.59 | 14 | 6.60E-06 | 3.10E-05 |
| 195 | SAFB | 30346 | 107 | 2368 | 8.35 | 23 | 7.00E-06 | 3.30E-05 |
| 196 | EEF1A1 | 30346 | 255 | 2368 | 19.9 | 41 | 7.80E-06 | 3.60E-05 |
| 197 | SUPT3H | 30346 | 30 | 2368 | 2.34 | 11 | 8.60E-06 | 4.00E-05 |
| 198 | ASB8 | 30346 | 66 | 2368 | 5.15 | 17 | 8.70E-06 | 4.00E-05 |
| 199 | SMARCA1 | 30346 | 73 | 2368 | 5.7 | 18 | 9.20E-06 | 4.20E-05 |
| 200 | HTATIP2 | 30346 | 42 | 2368 | 3.28 | 13 | 1.10E-05 | 5.20E-05 |
| 201 | HES1 | 30346 | 31 | 2368 | 2.42 | 11 | 1.20E-05 | 5.60E-05 |
| 202 | BOLA1 | 30346 | 31 | 2368 | 2.42 | 11 | 1.20E-05 | 5.60E-05 |
| 203 | BRPF1 | 30346 | 31 | 2368 | 2.42 | 11 | 1.20E-05 | 5.60E-05 |
| 204 | MTA2 | 30346 | 89 | 2368 | 6.94 | 20 | 1.40E-05 | 6.00E-05 |
| 205 | LMO4 | 30346 | 82 | 2368 | 6.4 | 19 | 1.40E-05 | 6.20E-05 |
| 206 | ZBTB5 | 30346 | 62 | 2368 | 4.84 | 16 | 1.50E-05 | 6.80E-05 |
| 207 | ZDHHC13 | 30346 | 120 | 2368 | 9.36 | 24 | 1.60E-05 | 7.10E-05 |
| 208 | HIC2 | 30346 | 76 | 2368 | 5.93 | 18 | 1.70E-05 | 7.40E-05 |
| 209 | YEATS4 | 30346 | 161 | 2368 | 12.56 | 29 | 1.90E-05 | 8.20E-05 |
| 210 | ID3 | 30346 | 107 | 2368 | 8.35 | 22 | 2.30E-05 | 9.90E-05 |
| 211 | HHEX | 30346 | 71 | 2368 | 5.54 | 17 | 2.50E-05 | 1.10E-04 |
| 212 | ZSCAN18 | 30346 | 39 | 2368 | 3.04 | 12 | 2.60E-05 | 1.10E-04 |
| 213 | BTAF1 | 30346 | 58 | 2368 | 4.53 | 15 | 2.80E-05 | 1.20E-04 |
| 214 | XPC | 30346 | 58 | 2368 | 4.53 | 15 | 2.80E-05 | 1.20E-04 |
| 215 | ATF6 | 30346 | 23 | 2368 | 1.79 | 9 | 3.10E-05 | 1.30E-04 |
| 216 | TSG101 | 30346 | 66 | 2368 | 5.15 | 16 | 3.60E-05 | 1.50E-04 |
| 217 | SLC2A4RG | 30346 | 73 | 2368 | 5.7 | 17 | 3.60E-05 | 1.50E-04 |
| 218 | TFPT | 30346 | 60 | 2368 | 4.68 | 15 | 4.20E-05 | 1.80E-04 |
| 219 | TOB1 | 30346 | 24 | 2368 | 1.87 | 9 | 4.70E-05 | 1.90E-04 |
| 220 | NCOA6 | 30346 | 19 | 2368 | 1.48 | 8 | 4.70E-05 | 1.90E-04 |
| 221 | CNOT2 | 30346 | 24 | 2368 | 1.87 | 9 | 4.70E-05 | 1.90E-04 |
| 222 | GTF2H4 | 30346 | 30 | 2368 | 2.34 | 10 | 5.80E-05 | 2.40E-04 |
| 223 | HEY2 | 30346 | 62 | 2368 | 4.84 | 15 | 6.40E-05 | 2.60E-04 |
| 224 | TBX21 | 30346 | 62 | 2368 | 4.84 | 15 | 6.40E-05 | 2.60E-04 |
| 225 | AES | 30346 | 123 | 2368 | 9.6 | 23 | 7.30E-05 | 2.90E-04 |
| 226 | ATF4 | 30346 | 70 | 2368 | 5.46 | 16 | 7.70E-05 | 3.10E-04 |
| 227 | POLR2L | 30346 | 50 | 2368 | 3.9 | 13 | 8.80E-05 | 3.50E-04 |
| 228 | BCL3 | 30346 | 57 | 2368 | 4.45 | 14 | 9.30E-05 | 3.70E-04 |
| 229 | ASCL1 | 30346 | 16 | 2368 | 1.25 | 7 | 1.10E-04 | 4.20E-04 |
| 230 | TFDP1 | 30346 | 38 | 2368 | 2.97 | 11 | 1.10E-04 | 4.20E-04 |
| 231 | PHF14 | 30346 | 58 | 2368 | 4.53 | 14 | 1.10E-04 | 4.50E-04 |
| 232 | ETV6 | 30346 | 95 | 2368 | 7.41 | 19 | 1.20E-04 | 4.70E-04 |
| 233 | RNF141 | 30346 | 52 | 2368 | 4.06 | 13 | 1.40E-04 | 5.30E-04 |
| 234 | H1FX | 30346 | 46 | 2368 | 3.59 | 12 | 1.60E-04 | 6.10E-04 |
| 235 | ANKRA2 | 30346 | 46 | 2368 | 3.59 | 12 | 1.60E-04 | 6.10E-04 |
| 236 | TOP2B | 30346 | 40 | 2368 | 3.12 | 11 | 1.80E-04 | 6.80E-04 |
| 237 | IVNS1ABP | 30346 | 28 | 2368 | 2.18 | 9 | 1.90E-04 | 7.20E-04 |
| 238 | TBC1D22A | 30346 | 34 | 2368 | 2.65 | 10 | 1.90E-04 | 7.20E-04 |
| 239 | DRAP1 | 30346 | 68 | 2368 | 5.31 | 15 | 2.00E-04 | 7.50E-04 |
| 240 | ZBTB20 | 30346 | 47 | 2368 | 3.67 | 12 | 2.00E-04 | 7.50E-04 |
| 241 | HEYL | 30346 | 69 | 2368 | 5.38 | 15 | 2.30E-04 | 8.70E-04 |
| 242 | POLR2D | 30346 | 69 | 2368 | 5.38 | 15 | 2.30E-04 | 8.70E-04 |
| 243 | PHB2 | 30346 | 35 | 2368 | 2.73 | 10 | 2.50E-04 | 9.20E-04 |
| 244 | ZNF442 | 30346 | 55 | 2368 | 4.29 | 13 | 2.50E-04 | 9.30E-04 |
| 245 | DBP | 30346 | 18 | 2368 | 1.4 | 7 | 2.60E-04 | 9.60E-04 |
| 246 | MTF1 | 30346 | 42 | 2368 | 3.28 | 11 | 2.80E-04 | 1.00E-03 |
| 247 | PPARG | 30346 | 49 | 2368 | 3.82 | 12 | 3.00E-04 | 1.10E-03 |
| 248 | PRDM9 | 30346 | 36 | 2368 | 2.81 | 10 | 3.20E-04 | 1.20E-03 |
| 249 | ELL2 | 30346 | 24 | 2368 | 1.87 | 8 | 3.20E-04 | 1.20E-03 |
| 250 | MYC | 30346 | 64 | 2368 | 4.99 | 14 | 3.50E-04 | 1.30E-03 |
| 251 | SP3 | 30346 | 87 | 2368 | 6.79 | 17 | 3.60E-04 | 1.30E-03 |
| 252 | ZBTB40 | 30346 | 50 | 2368 | 3.9 | 12 | 3.70E-04 | 1.30E-03 |
| 253 | ZNF143 | 30346 | 19 | 2368 | 1.48 | 7 | 3.80E-04 | 1.40E-03 |
| 254 | SIX5 | 30346 | 37 | 2368 | 2.89 | 10 | 4.00E-04 | 1.40E-03 |
| 255 | LMO2 | 30346 | 88 | 2368 | 6.87 | 17 | 4.10E-04 | 1.50E-03 |
| 256 | CBX8 | 30346 | 31 | 2368 | 2.42 | 9 | 4.40E-04 | 1.60E-03 |
| 257 | TAF1B | 30346 | 59 | 2368 | 4.6 | 13 | 5.20E-04 | 1.80E-03 |
| 258 | JUNB | 30346 | 90 | 2368 | 7.02 | 17 | 5.40E-04 | 1.90E-03 |
| 259 | CRABP2 | 30346 | 32 | 2368 | 2.5 | 9 | 5.70E-04 | 2.00E-03 |
| 260 | ZFP36 | 30346 | 32 | 2368 | 2.5 | 9 | 5.70E-04 | 2.00E-03 |
| 261 | ZNF586 | 30346 | 32 | 2368 | 2.5 | 9 | 5.70E-04 | 2.00E-03 |
| 262 | SALL2 | 30346 | 26 | 2368 | 2.03 | 8 | 5.90E-04 | 2.00E-03 |
| 263 | AR | 30346 | 26 | 2368 | 2.03 | 8 | 5.90E-04 | 2.00E-03 |
| 264 | HSF2BP | 30346 | 26 | 2368 | 2.03 | 8 | 5.90E-04 | 2.00E-03 |
| 265 | LEF1 | 30346 | 83 | 2368 | 6.48 | 16 | 6.10E-04 | 2.10E-03 |
| 266 | MTA1 | 30346 | 46 | 2368 | 3.59 | 11 | 6.60E-04 | 2.30E-03 |
| 267 | HAX1 | 30346 | 46 | 2368 | 3.59 | 11 | 6.60E-04 | 2.30E-03 |
| 268 | FHL2 | 30346 | 76 | 2368 | 5.93 | 15 | 6.90E-04 | 2.40E-03 |
| 269 | PBX1 | 30346 | 21 | 2368 | 1.64 | 7 | 7.60E-04 | 2.60E-03 |
| 270 | SIX3 | 30346 | 21 | 2368 | 1.64 | 7 | 7.60E-04 | 2.60E-03 |
| 271 | NFIL3 | 30346 | 27 | 2368 | 2.11 | 8 | 7.80E-04 | 2.60E-03 |
| 272 | POLR2F | 30346 | 27 | 2368 | 2.11 | 8 | 7.80E-04 | 2.60E-03 |
| 273 | NPAS1 | 30346 | 27 | 2368 | 2.11 | 8 | 7.80E-04 | 2.60E-03 |
| 274 | TRIM33 | 30346 | 27 | 2368 | 2.11 | 8 | 7.80E-04 | 2.60E-03 |
| 275 | LZTR1 | 30346 | 40 | 2368 | 3.12 | 10 | 7.90E-04 | 2.60E-03 |
| 276 | BTG1 | 30346 | 47 | 2368 | 3.67 | 11 | 8.00E-04 | 2.60E-03 |
| 277 | TSC22D1 | 30346 | 77 | 2368 | 6.01 | 15 | 8.00E-04 | 2.60E-03 |
| 278 | CREG1 | 30346 | 110 | 2368 | 8.58 | 19 | 8.30E-04 | 2.70E-03 |
| 279 | ELF1 | 30346 | 70 | 2368 | 5.46 | 14 | 9.00E-04 | 2.90E-03 |
| 280 | BTF3 | 30346 | 128 | 2368 | 9.99 | 21 | 9.10E-04 | 3.00E-03 |
| 281 | ZNF358 | 30346 | 55 | 2368 | 4.29 | 12 | 9.20E-04 | 3.00E-03 |
| 282 | CENPB | 30346 | 48 | 2368 | 3.75 | 11 | 9.70E-04 | 3.10E-03 |
| 283 | SMARCC2 | 30346 | 41 | 2368 | 3.2 | 10 | 9.70E-04 | 3.10E-03 |
| 284 | ZNF408 | 30346 | 22 | 2368 | 1.72 | 7 | 1.00E-03 | 3.40E-03 |
| 285 | NFKB2 | 30346 | 35 | 2368 | 2.73 | 9 | 1.20E-03 | 3.70E-03 |
| 286 | ZNF688 | 30346 | 64 | 2368 | 4.99 | 13 | 1.20E-03 | 3.70E-03 |
| 287 | FOXA1 | 30346 | 42 | 2368 | 3.28 | 10 | 1.20E-03 | 3.80E-03 |
| 288 | SIRT5 | 30346 | 42 | 2368 | 3.28 | 10 | 1.20E-03 | 3.80E-03 |
| 289 | TAF10 | 30346 | 17 | 2368 | 1.33 | 6 | 1.30E-03 | 4.10E-03 |
| 290 | GTF2F2 | 30346 | 29 | 2368 | 2.26 | 8 | 1.30E-03 | 4.10E-03 |
| 291 | USF2 | 30346 | 17 | 2368 | 1.33 | 6 | 1.30E-03 | 4.10E-03 |
| 292 | SCAND1 | 30346 | 29 | 2368 | 2.26 | 8 | 1.30E-03 | 4.10E-03 |
| 293 | MAF | 30346 | 23 | 2368 | 1.79 | 7 | 1.40E-03 | 4.40E-03 |
| 294 | HOXB5 | 30346 | 36 | 2368 | 2.81 | 9 | 1.40E-03 | 4.40E-03 |
| 295 | MKL1 | 30346 | 43 | 2368 | 3.36 | 10 | 1.40E-03 | 4.50E-03 |
| 296 | NR3C2 | 30346 | 82 | 2368 | 6.4 | 15 | 1.60E-03 | 4.80E-03 |
| 297 | SREBF1 | 30346 | 74 | 2368 | 5.77 | 14 | 1.60E-03 | 4.90E-03 |
| 298 | PAWR | 30346 | 30 | 2368 | 2.34 | 8 | 1.70E-03 | 5.10E-03 |
| 299 | PBXIP1 | 30346 | 44 | 2368 | 3.43 | 10 | 1.70E-03 | 5.30E-03 |
| 300 | ZNHIT3 | 30346 | 171 | 2368 | 13.34 | 25 | 1.70E-03 | 5.30E-03 |
| 301 | NFX1 | 30346 | 59 | 2368 | 4.6 | 12 | 1.80E-03 | 5.30E-03 |
| 302 | TRIP6 | 30346 | 37 | 2368 | 2.89 | 9 | 1.80E-03 | 5.30E-03 |
| 303 | SMAD7 | 30346 | 31 | 2368 | 2.42 | 8 | 2.10E-03 | 6.30E-03 |
| 304 | RBL2 | 30346 | 110 | 2368 | 8.58 | 18 | 2.10E-03 | 6.30E-03 |
| 305 | NFKBIA | 30346 | 38 | 2368 | 2.97 | 9 | 2.20E-03 | 6.40E-03 |
| 306 | PEG3 | 30346 | 61 | 2368 | 4.76 | 12 | 2.40E-03 | 7.10E-03 |
| 307 | DAXX | 30346 | 25 | 2368 | 1.95 | 7 | 2.40E-03 | 7.10E-03 |
| 308 | TSC22D3 | 30346 | 25 | 2368 | 1.95 | 7 | 2.40E-03 | 7.10E-03 |
| 309 | NKRF | 30346 | 19 | 2368 | 1.48 | 6 | 2.50E-03 | 7.40E-03 |
| 310 | TCF4 | 30346 | 32 | 2368 | 2.5 | 8 | 2.60E-03 | 7.70E-03 |
| 311 | FXR1 | 30346 | 32 | 2368 | 2.5 | 8 | 2.60E-03 | 7.70E-03 |
| 312 | PBX3 | 30346 | 62 | 2368 | 4.84 | 12 | 2.70E-03 | 8.00E-03 |
| 313 | ZKSCAN3 | 30346 | 26 | 2368 | 2.03 | 7 | 3.10E-03 | 8.90E-03 |
| 314 | EMX1 | 30346 | 26 | 2368 | 2.03 | 7 | 3.10E-03 | 8.90E-03 |
| 315 | ZFP36L1 | 30346 | 141 | 2368 | 11 | 21 | 3.10E-03 | 9.00E-03 |
| 316 | HCFC1 | 30346 | 63 | 2368 | 4.92 | 12 | 3.10E-03 | 9.10E-03 |
| 317 | NCOA3 | 30346 | 88 | 2368 | 6.87 | 15 | 3.20E-03 | 9.20E-03 |
| 318 | PBX2 | 30346 | 33 | 2368 | 2.58 | 8 | 3.20E-03 | 9.20E-03 |
| 319 | ZKSCAN1 | 30346 | 20 | 2368 | 1.56 | 6 | 3.30E-03 | 9.50E-03 |
| 320 | MYBL1 | 30346 | 20 | 2368 | 1.56 | 6 | 3.30E-03 | 9.50E-03 |
| 321 | HMGB3 | 30346 | 48 | 2368 | 3.75 | 10 | 3.40E-03 | 9.80E-03 |
| 322 | HIPK2 | 30346 | 56 | 2368 | 4.37 | 11 | 3.60E-03 | 1.00E-02 |
| 323 | ZNF337 | 30346 | 41 | 2368 | 3.2 | 9 | 3.70E-03 | 1.10E-02 |
| 324 | FOXD2 | 30346 | 41 | 2368 | 3.2 | 9 | 3.70E-03 | 1.10E-02 |
| 325 | TBP | 30346 | 27 | 2368 | 2.11 | 7 | 3.90E-03 | 1.10E-02 |
| 326 | FOXJ3 | 30346 | 27 | 2368 | 2.11 | 7 | 3.90E-03 | 1.10E-02 |
| 327 | ELL | 30346 | 21 | 2368 | 1.64 | 6 | 4.40E-03 | 1.20E-02 |
| 328 | BCL6 | 30346 | 21 | 2368 | 1.64 | 6 | 4.40E-03 | 1.20E-02 |
| 329 | RUVBL2 | 30346 | 21 | 2368 | 1.64 | 6 | 4.40E-03 | 1.20E-02 |
| 330 | RFX5 | 30346 | 42 | 2368 | 3.28 | 9 | 4.40E-03 | 1.20E-02 |
| 331 | ZNF444 | 30346 | 58 | 2368 | 4.53 | 11 | 4.70E-03 | 1.30E-02 |
| 332 | NR2F6 | 30346 | 28 | 2368 | 2.18 | 7 | 4.80E-03 | 1.30E-02 |
| 333 | ILF3 | 30346 | 215 | 2368 | 16.78 | 28 | 5.30E-03 | 1.40E-02 |
| 334 | HMG20B | 30346 | 51 | 2368 | 3.98 | 10 | 5.40E-03 | 1.50E-02 |
| 335 | DDX5 | 30346 | 51 | 2368 | 3.98 | 10 | 5.40E-03 | 1.50E-02 |
| 336 | E2F1 | 30346 | 59 | 2368 | 4.6 | 11 | 5.40E-03 | 1.50E-02 |
| 337 | CNOT3 | 30346 | 51 | 2368 | 3.98 | 10 | 5.40E-03 | 1.50E-02 |
| 338 | POLR3B | 30346 | 22 | 2368 | 1.72 | 6 | 5.60E-03 | 1.50E-02 |
| 339 | TAF6 | 30346 | 22 | 2368 | 1.72 | 6 | 5.60E-03 | 1.50E-02 |
| 340 | PPAN | 30346 | 22 | 2368 | 1.72 | 6 | 5.60E-03 | 1.50E-02 |
| 341 | EGR2 | 30346 | 112 | 2368 | 8.74 | 17 | 6.10E-03 | 1.60E-02 |
| 342 | STAT4 | 30346 | 16 | 2368 | 1.25 | 5 | 6.10E-03 | 1.60E-02 |
| 343 | NR3C1 | 30346 | 112 | 2368 | 8.74 | 17 | 6.10E-03 | 1.60E-02 |
| 344 | NAB1 | 30346 | 44 | 2368 | 3.43 | 9 | 6.10E-03 | 1.60E-02 |
| 345 | ECD | 30346 | 52 | 2368 | 4.06 | 10 | 6.30E-03 | 1.60E-02 |
| 346 | NFE2L1 | 30346 | 52 | 2368 | 4.06 | 10 | 6.30E-03 | 1.60E-02 |
| 347 | PRRX1 | 30346 | 52 | 2368 | 4.06 | 10 | 6.30E-03 | 1.60E-02 |
| 348 | NFIX | 30346 | 77 | 2368 | 6.01 | 13 | 6.30E-03 | 1.70E-02 |
| 349 | ZBTB33 | 30346 | 23 | 2368 | 1.79 | 6 | 7.10E-03 | 1.90E-02 |
| 350 | ASB9 | 30346 | 23 | 2368 | 1.79 | 6 | 7.10E-03 | 1.90E-02 |
| 351 | ASXL1 | 30346 | 30 | 2368 | 2.34 | 7 | 7.20E-03 | 1.90E-02 |
| 352 | ASCC2 | 30346 | 87 | 2368 | 6.79 | 14 | 7.30E-03 | 1.90E-02 |
| 353 | CHD1 | 30346 | 96 | 2368 | 7.49 | 15 | 7.40E-03 | 1.90E-02 |
| 354 | SSBP2 | 30346 | 31 | 2368 | 2.42 | 7 | 8.70E-03 | 2.20E-02 |
| 355 | PCBD1 | 30346 | 89 | 2368 | 6.94 | 14 | 8.90E-03 | 2.30E-02 |
| 356 | ZNF165 | 30346 | 63 | 2368 | 4.92 | 11 | 9.00E-03 | 2.30E-02 |
| 357 | ADNP | 30346 | 145 | 2368 | 11.31 | 20 | 9.10E-03 | 2.30E-02 |
| 358 | SP1 | 30346 | 136 | 2368 | 10.61 | 19 | 9.50E-03 | 2.40E-02 |
| 359 | CSRP2 | 30346 | 47 | 2368 | 3.67 | 9 | 9.60E-03 | 2.40E-02 |
| 360 | ZNF20 | 30346 | 47 | 2368 | 3.67 | 9 | 9.60E-03 | 2.40E-02 |
| 361 | CBFB | 30346 | 32 | 2368 | 2.5 | 7 | 1.00E-02 | 2.60E-02 |
| 362 | BTG2 | 30346 | 56 | 2368 | 4.37 | 10 | 1.10E-02 | 2.70E-02 |
| 363 | XRCC3 | 30346 | 25 | 2368 | 1.95 | 6 | 1.10E-02 | 2.70E-02 |
| 364 | ATF1 | 30346 | 25 | 2368 | 1.95 | 6 | 1.10E-02 | 2.70E-02 |
| 365 | ONECUT1 | 30346 | 65 | 2368 | 5.07 | 11 | 1.10E-02 | 2.80E-02 |
| 366 | TCF3 | 30346 | 57 | 2368 | 4.45 | 10 | 1.20E-02 | 3.00E-02 |
| 367 | NR1H3 | 30346 | 33 | 2368 | 2.58 | 7 | 1.20E-02 | 3.00E-02 |
| 368 | ZNF133 | 30346 | 33 | 2368 | 2.58 | 7 | 1.20E-02 | 3.00E-02 |
| 369 | SMAD4 | 30346 | 41 | 2368 | 3.2 | 8 | 1.30E-02 | 3.10E-02 |
| 370 | PITX1 | 30346 | 26 | 2368 | 2.03 | 6 | 1.30E-02 | 3.30E-02 |
| 371 | IRF9 | 30346 | 67 | 2368 | 5.23 | 11 | 1.40E-02 | 3.50E-02 |
| 372 | ZSCAN5A | 30346 | 34 | 2368 | 2.65 | 7 | 1.40E-02 | 3.50E-02 |
| 373 | EN2 | 30346 | 34 | 2368 | 2.65 | 7 | 1.40E-02 | 3.50E-02 |
| 374 | TBX3 | 30346 | 104 | 2368 | 8.12 | 15 | 1.50E-02 | 3.70E-02 |
| 375 | HABP4 | 30346 | 77 | 2368 | 6.01 | 12 | 1.60E-02 | 3.80E-02 |
| 376 | AIP | 30346 | 27 | 2368 | 2.11 | 6 | 1.60E-02 | 3.90E-02 |
| 377 | TRIM24 | 30346 | 20 | 2368 | 1.56 | 5 | 1.70E-02 | 4.00E-02 |
| 378 | RNPS1 | 30346 | 43 | 2368 | 3.36 | 8 | 1.70E-02 | 4.10E-02 |
| 379 | SMAD5 | 30346 | 35 | 2368 | 2.73 | 7 | 1.70E-02 | 4.10E-02 |
| 380 | GTF2E1 | 30346 | 78 | 2368 | 6.09 | 12 | 1.70E-02 | 4.20E-02 |
| 381 | CREBL2 | 30346 | 61 | 2368 | 4.76 | 10 | 1.90E-02 | 4.50E-02 |
| 382 | ARID5B | 30346 | 70 | 2368 | 5.46 | 11 | 1.90E-02 | 4.60E-02 |
| 383 | PMS1 | 30346 | 36 | 2368 | 2.81 | 7 | 2.00E-02 | 4.70E-02 |
| 384 | SETBP1 | 30346 | 53 | 2368 | 4.14 | 9 | 2.00E-02 | 4.90E-02 |
| 385 | ZNF76 | 30346 | 71 | 2368 | 5.54 | 11 | 2.10E-02 | 5.00E-02 |
| 386 | PIAS3 | 30346 | 71 | 2368 | 5.54 | 11 | 2.10E-02 | 5.00E-02 |
